# Evolution and patterns of global health financing 1995–2014: development assistance for health, and government, prepaid private, and out-of-pocket health spending in 184 countries

**DOI:** 10.1016/S0140-6736(17)30874-7

**Published:** 2017-05-20

**Authors:** Global Burden of Disease Health Financing Collaborator Network, Joseph Dieleman, Madeline Campbell, Abigail Chapin, Erika Eldrenkamp, Victoria Y Fan, Annie Haakenstad, Jennifer Kates, Yingying Liu, Taylor Matyasz, Angela Micah, Alex Reynolds, Nafis Sadat, Matthew T Schneider, Reed Sorensen, Tim Evans, David Evans, Christoph Kurowski, Ajay Tandon, Kaja M Abbas, Semaw Ferede Abera, Aliasghar Ahmad Kiadaliri, Kedir Yimam Ahmed, Muktar Beshir Ahmed, Khurshid Alam, Reza Alizadeh-Navaei, Ala'a Alkerwi, Erfan Amini, Walid Ammar, Stephen Marc Amrock, Carl Abelardo T Antonio, Tesfay Mehari Atey, Leticia Avila-Burgos, Ashish Awasthi, Aleksandra Barac, Oscar Alberto Bernal, Addisu Shunu Beyene, Tariku Jibat Beyene, Charles Birungi, Habtamu Mellie Bizuayehu, Nicholas J K Breitborde, Lucero Cahuana-Hurtado, Ruben Estanislao Castro, Ferran Catalia-Lopez, Koustuv Dalal, Lalit Dandona, Rakhi Dandona, Pieter de Jager, Samath D Dharmaratne, Manisha Dubey, Carla Sofia e Sa Farinha, Andre Faro, Andrea B Feigl, Florian Fischer, Joseph Robert Anderson Fitchett, Nataliya Foigt, Ababi Zergaw Giref, Rahul Gupta, Samer Hamidi, Hilda L Harb, Simon I Hay, Delia Hendrie, Masako Horino, Mikk Jürisson, Mihajlo B Jakovljevic, Mehdi Javanbakht, Denny John, Jost B Jonas, Seyed M. Karimi, Young-Ho Khang, Jagdish Khubchandani, Yun Jin Kim, Jonas M Kinge, Kristopher J Krohn, G Anil Kumar, Hassan Magdy Abd El Razek, Mohammed Magdy Abd El Razek, Azeem Majeed, Reza Malekzadeh, Felix Masiye, Toni Meier, Atte Meretoja, Ted R Miller, Erkin M Mirrakhimov, Shafiu Mohammed, Vinay Nangia, Stefano Olgiati, Abdalla Sidahmed Osman, Mayowa O Owolabi, Tejas Patel, Angel J Paternina Caicedo, David M Pereira, Julian Perelman, Suzanne Polinder, Anwar Rafay, Vafa Rahimi-Movaghar, Rajesh Kumar Rai, Usha Ram, Chhabi Lal Ranabhat, Hirbo Shore Roba, Joseph Salama, Miloje Savic, Sadaf G Sepanlou, Mark G Shrime, Roberto Tchio Talongwa, Braden J Te Ao, Fabrizio Tediosi, Azeb Gebresilassie Tesema, Alan J Thomson, Ruoyan Tobe-Gai, Roman Topor-Madry, Eduardo A Undurraga, Tommi Vasankari, Francesco S Violante, Andrea Werdecker, Tissa Wijeratne, Gelin Xu, Naohiro Yonemoto, Mustafa Z Younis, Chuanhua Yu, Zoubida Zaidi, Maysaa El Sayed Zaki, Christopher J L Murray

## Abstract

**Background:**

An adequate amount of prepaid resources for health is important to ensure access to health services and for the pursuit of universal health coverage. Previous studies on global health financing have described the relationship between economic development and health financing. In this study, we further explore global health financing trends and examine how the sources of funds used, types of services purchased, and development assistance for health disbursed change with economic development. We also identify countries that deviate from the trends.

**Methods:**

We estimated national health spending by type of care and by source, including development assistance for health, based on a diverse set of data including programme reports, budget data, national estimates, and 964 National Health Accounts. These data represent health spending for 184 countries from 1995 through 2014. We converted these data into a common inflation-adjusted and purchasing power-adjusted currency, and used non-linear regression methods to model the relationship between health financing, time, and economic development.

**Findings:**

Between 1995 and 2014, economic development was positively associated with total health spending and a shift away from a reliance on development assistance and out-of-pocket (OOP) towards government spending. The largest absolute increase in spending was in high-income countries, which increased to purchasing power-adjusted $5221 per capita based on an annual growth rate of 3·0%. The largest health spending growth rates were in upper-middle-income (5·9) and lower-middle-income groups (5·0), which both increased spending at more than 5% per year, and spent $914 and $267 per capita in 2014, respectively. Spending in low-income countries grew nearly as fast, at 4·6%, and health spending increased from $51 to $120 per capita. In 2014, 59·2% of all health spending was financed by the government, although in low-income and lower-middle-income countries, 29·1% and 58·0% of spending was OOP spending and 35·7% and 3·0% of spending was development assistance. Recent growth in development assistance for health has been tepid; between 2010 and 2016, it grew annually at 1·8%, and reached US$37·6 billion in 2016. Nonetheless, there is a great deal of variation revolving around these averages. 29 countries spend at least 50% more than expected per capita, based on their level of economic development alone, whereas 11 countries spend less than 50% their expected amount.

**Interpretation:**

Health spending remains disparate, with low-income and lower-middle-income countries increasing spending in absolute terms the least, and relying heavily on OOP spending and development assistance. Moreover, tremendous variation shows that neither time nor economic development guarantee adequate prepaid health resources, which are vital for the pursuit of universal health coverage.

**Funding:**

The Bill & Melinda Gates Foundation.

## Introduction

Substantial disparities characterise the amount of resources spent on health globally.^1^ In many low-income countries, per capita spending remains less than $100 per year, and inadequate resources prevent people from accessing quality health care. By contrast, in many high-income countries, annual health spending has ballooned to higher than $5000 per capita. These divergent contexts are reflected in the thrust of public policy, with calls to rein in health spending growth in high-income countries, and appeals to mobilise more resources for health in low-income and middle-income countries.^2^, ^3^, ^4^, ^5^, ^6^, ^7^, ^8^

Notwithstanding these disparities, certain trends underpin health spending worldwide.^9^, ^10^, ^11^ The health financing transition is defined by changes in the level and composition of health spending. These changes are associated with economic development. More specifically, the health financing transition has two key features, observed across countries and across time: (1) as countries experience economic development, they spend more per capita on health; and (2) less of that spending is out-of-pocket (OOP).^12^, ^13^, ^14^ It has been theorised that both features of the health financing transition are driven by per capita income growth, technological advances, maturation of health financing system, greater government fiscal capacity, introduction of social health insurance, and population ageing, all associated with socioeconomic development. The first feature—increasing spending on health per capita—is important because it leads to more resources for health, which can lead to improved access to higher quality health services. The second feature—the declining share of health spending that is OOP—is important because high OOP payments can deter use of health-care services, which can lead to poor health outcomes and medical impoverishment.^15^, ^16^, ^17^, ^18^, ^19^, ^20^, ^21^

**Research in context**

**Evidence before this study**

Understanding how national health spending in total, and disaggregated into government, prepaid private, out-of-pocket (OOP), and development assistance for health, is associated with economic development is important for assessing progress and determining fiscally appropriate financing targets. Deviation from these trends can be used to identify opportunities for increased focus and attention from development partners and domestic governments. Adequate health financing that is prepaid, rather than OOP, is crucial for the pursuit of universal health coverage.

Several studies have tracked national health spending patterns over time. Typically, these studies are descriptive (cross-sectional and time series) and focus on trends in growth in health spending and its determinants. A key piece of research on this topic was completed by Fan and Savedoff. They explored spending in 126 countries spanning 15 years and identified key patterns in how growth in health spending evolves over time, especially in relation to per capita income. They used the term “health financing transition” to characterise two observed patterns: as income rises, (1) health spending per capita tends to rise, and (2) the share of health payments that are OOP tends to decrease.

**Added value of this study**

This research expands what is known about the health financing transition and the availability of prepaid health resources in three distinct ways. First, this research provides a more comprehensive perspective on the health financing patterns associated with economic development. Previously our research on this topic has focused on development assistance for health. In this research we combine these data with domestic spending estimates to generate a panel of data spanning 20 years and 184 countries. Along with development assistance for health, this research measures government health spending that is domestically generated, prepaid private health spending, and out-of-pocket health spending. Second, we use non-linear methods to assess important non-linear variation across time and levels of economic development. These trends allow us to explore how health spending from development partners fit with the health financing transition framework. This addition is crucial to explore the effect that the transition away from a reliance on development assistance for health has on health-system financing. Relative to previous research, this work extends the analysis to use more comprehensive data that focuses on the source of the health spending, uses robust non-linear estimation methods, and highlights both trends and deviations from those trends for 184 countries. Third, we explore how the types of health-related goods and services purchased by each country evolve with economic development.

**Implications of all the available evidence**

The findings from this study expand the understanding of the health financing transition. We measure health financing trends associated with economic development: health spending increases exponentially, while the share of total health spending that is OOP declines, especially at the highest income levels. The share of financing from development assistance for health, on average, declines before government spending becomes the dominant funding source, leaving a period in the transition where countries often rely heavily on both OOP and government spending. The share of spending on each type of goods and service is relatively stable across the development spectrum with substantive emergence of long-term care spending at the highest income levels. Despite the health financing trends associated with economic development, tremendous variation exists and suggests that neither time nor economic development guarantee adequate prepaid health resources will be available. Deviations from the health spending trends can identify health financing opportunities. Furthermore, in view of the complexity of translating growth into health spending, proactive steps will be needed in some places to mobilise adequate prepaid resources for the pursuit of universal health coverage.

Changes in a country's reliance on development assistance for health is also related to economic development. Development assistance for health tends to be phased out as countries develop economically, especially when measured as a share of total health spending. Eligibility criteria set by donors, such as the Global Fund for AIDS, Tuberculosis, and Malaria (the Global Fund), the World Bank's International Development Association, and Gavi, the Vaccine Alliance (Gavi), are linked to gross national income thresholds.^22^ At a certain level of economic development, countries become ineligible to receive development assistance for health.

To explore the global health financing landscape and the patterns described by the health financing transition framework, this study examines spending on health in 184 countries over a 20-year period, 1995 to 2014. These data capture the distinct health spending trends of low-income, middle-income, and high-income countries. We track total health spending and health spending disaggregated by the source of the funds—spending originating from governments, development partners, OOP, and prepaid private pools, such as private insurance. To highlight changes in the mix of goods and services purchased, health spending has also been disaggregated by type of goods and services purchased, such as inpatient, outpatient, and pharmaceutical spending. Our analyses focus on patterns associated with economic development and, importantly, highlight deviations from observed trends. We also assess how development assistance for health is disbursed globally and discuss how development assistance for health is a part of and affects the health financing transition. These data span from 1990 to 2016, and highlight the sources and development agencies that provide development assistance for health. Primary health focus areas and recipient regions targeted by the development assistance is also tracked to show who is receiving development assistance for health and what focus areas are being prioritised.

## Methods

### Total health spending and government, prepaid private, and out-of-pocket health spending

Health spending stems from four sources: the government, which includes general government budgets and social health insurance; prepaid private spending, which includes private insurance and non-governmental organisation spending; OOP payments; and development assistance for health. The sum of these sources make up total health-care spending. Government, prepaid private, and OOP spending data were extracted from the WHO Global Health Observatory. These data measure the sum of all outlays for health maintenance, restoration, or enhancement paid for in cash or supplied in kind.^23^ This excludes indirect health spending, such as lost wages due to illness or transportation costs; spending on informal care, such as care provided by a family member; spending on traditional healers; and illegal so-called black market or under the table transactions, such as bribes. Spending estimates were extracted in national currency units and divided by gross domestic product (GDP), also reported in national currency units and reported by WHO. This fraction was multiplied by GDP per capita reported in inflation-adjusted 2015 PPP $.^24^

WHO data tracks spending by agent, such that it is unclear if government and prepaid private spending were sourced domestically. To differentiate between domestically and internationally financed spending, development assistance for health provided to the government was removed from WHO's government spending estimates and development assistance for health provided to non-government providers or organisations was removed from WHO's prepaid private estimates.^25^

For the 184 countries, between 1995 and 2014, 1·7%, 14·8%, and 1·7% of the government, prepaid private, and OOP health spending estimates were missing, respectively. These estimates were imputed in *R* using Amelia II: A program for missing data (version 1·7·4), and more information about these methods is provided in the appendix (p 19).^26^ The result of this approach is four mutually exclusive, collectively exhaustive spending estimates by source and time—government as source, prepaid private (excluding donor financing), OOP health spending, and development assistance for health. These four series were summed to form annual estimates of total health spending for each of the 184 countries, from 1995 through 2014.

### Development assistance for health

Development assistance for health is the financial and in-kind resources transferred from development agencies to low-income and middle-income countries with the primary purpose of maintaining or improving health.^27^ Many of the methods used to estimate development assistance for health have been used and published previously, although the input data and some methods have been updated and improved for this study.^25^, ^27^, ^28^ These estimates are based on data from all publicly available databases tracking development assistance, including project-level records from the Organisation for Economic Co-operation and Development (OECD) and other development agencies, such as the World Bank, the Global Fund, Gavi, and the Bill & Melinda Gates Foundation. In addition, audited budget statements and annual reports are used to estimate development assistance for health through 2016. When disbursement data were not available, commitment data are adjusted to reflect disbursements. Disbursements are tracked comprehensively from source to disbursing agency, also known as the disbursing channel, to recipient to avoid double counting associated with development agencies transferring resources among themselves. These data are disaggregated based on the source of development assistance for health, disbursing channel, health focus area, and country recipient. Development assistance for health is disaggregated by health focus area—newborn and child health, maternal health, HIV/AIDS, malaria, tuberculosis, non-communicable diseases, other infectious diseases, health system strengthening, which includes discretionary grants to the health sector, and other and unallocable—and tracks disbursements from 1990 to 2016. Other includes projects that are for health projects that do not fit into any of the other health focus areas, while unallocable development assistance for health is that that does not have sufficient data to be disaggregated by health focus area. A more thorough presentation of these methods and explanation on how these methods defer from past research is provided in the appendix (p 41).

The Institute for Health Metrics and Evaluation's (IHME) development assistance for health database further disaggregates funds based on whether they are expected to be channelled to the recipient country's government, or are provided to non-governmental providers or organisations.^29^ Development assistance for health estimates are reported in 2015 US$, and were converted into 2015 purchasing-power-adjusted US$ to reflect the purchasing power of development assistance for health in the recipient country. Purchasing power parity exchange rates were based on data from the International Monetary Fund (IMF), World Bank, and WHO.

### Health spending by types of goods and services

Total health spending was also disaggregated by type of goods and services, such as inpatient or outpatient care. For this purpose, we collected all available National Health Account (NHA) reports. NHAs track health spending using an agreed upon accounting framework developed by the OECD, WHO, and Eurostat. The standards were first codified in 2001, but then updated in 2011 to what is now known as the System of Health Accounts (SHA) 2011.^30^, ^31^ A systematic review located, extracted, and published data from 872 reports spanning 1996 to 2010.^32^ We added to this review by searching WHO, OECD, and Eurostat databases, and Google for search terms “National Health Account” and “System of Health Account”, which identified 178 additional NHAs. The newly collected reports are primarily from more recent years.

We mapped estimates based on NHA 2001 standards to SHA 2011 with methods described in the appendix (p 20). Because the NHA 2001 and SHA 2011 standards for tracking spending on some preventive health services were irreconcilable, we include only the spending on immunisations and early disease detection. Excluded categories contain spending on education and counselling programmes, epidemiological surveillance, and disaster preparedness.

Of the 1050 NHA reports identified for this research, only 964 NHA reports included the necessary data (specific information about the exclusion criterion used to determine the set of used NHAs outlined in the appendix [p 20]). These 964 reports span 108 countries and range from 1995 to 2014. From these, we extracted total and government health spending by type of goods and services, and aggregated spending into eight categories: inpatient curative and rehabilitative care; day and outpatient curative and rehabilitative care; long-term care; ancillary services; medical goods, which includes pharmaceuticals; governance and health-system and financing administration; immunisation and early disease detection programmes; and other care. Estimates by type of service were generated as a share of both total health spending and total government health spending. Other care is a residual category that includes all health spending not included in the other categories.

### Gross domestic product data

GDP data spanning 1995 to 2015 were based on data collected from the International Monetary Fund, World Bank, the UN, the Maddison Project, and Penn World Tables database.^33^, ^34^, ^35^, ^36^ These data were combined using regression methods and previously developed for producing a complete GDP time series.^24^ GDP data were reported in inflation-adjusted 2015 PPP $.

### Spending by income groups and geographic regions

We report estimates aggregated by FY2016 World Bank income groups and Global Burden of Disease (GBD) super regions.^37^, ^38^ World Bank income groups are four mutually exclusive categories assigned by the World Bank and based on gross national income. GBD super regions are seven mutually exclusive categories based on geography and cause of death patterns. Spending estimates were constructed to reflect the group or region as a whole. For example, the group's health spending per capita is the group's total spending divided by total population. Similarly, the group's government health spending as a share of total spending was the group's total government spending divided by the group's total health spending.

### Statistical analyses

We completed three primary analyses. For all three analyses, we used multivariate penalised spline regression to allow for flexible and non-linear model fit across all 184 countries and 20 years of data.^39^ First, we regressed the natural log of total health spending per capita on the natural log of GDP per capita and time. Second, we regressed four health spending by source fractions—development assistance for health and government, prepaid private, and OOP health spending—on GDP per capita and time. Third, we regressed the eight health spending by type of goods and services fractions—inpatient curative and rehabilitative care, day and outpatient curative and rehabilitative care, long-term care, ancillary services, medical goods, governance and health-system and financing administration, immunisation and early disease detection programmes, and other care—on GDP per capita and time. For the second and third analyses, the spending fractions (by source and by type) were each measured as a share of total health spending and centre log-ratio transformed, while GDP per capita was natural log transformed.^40^ To estimate uncertainty, the underlying data was bootstrapped 1000 times, and all regressions were completed independently on each of the 1000 bootstrap samples.^41^, ^42^ Robustness checks included in the appendix (p 29) reinforce our qualitative conclusions, and use subsets of our data and also rely on the Socio-demographic Index and the Human Development Index, both of which track additional dimensions related to socioeconomic development, rather than simply GDP per capita which tracks economic development.

To measure countries' 2014 health spending relative to the expected value as determined by the fitted trend, we extracted the estimated country-year and year-specific residual (or error) from the regression analyses. The residuals measure the difference between the actual 2014 spending levels and the expected 2014 spending levels predicted by the model relative only to the country's GDP per capita. The residual measures the effect of country characteristics not included in the model, such as health burden, health-system policies, prices, and society's willingness to spend on health.

In addition to this, we tested whether health spending per capita grew exponentially with economic development. To test these we used ordinary least squares to regress the natural log of health spending per capita on the natural log of GDP per capita. More details on all estimation are included in the appendix (p 4).

### Role of the funding source

The funder of this study had no role in the study design, data collection, data analysis, data interpretation, or writing of the report. All authors had full access to the data in the study and JLD and CJLM had final responsibility for the decision to submit for publication.

## Results

### Health spending in 2014

Table 1 shows that in 2014 health spending per capita across all countries was $1279. This spending was concentrated in high-income countries, and ranged from $33 in Somalia to $9237 in the USA. These extremes, which are reported using 2015 PPP $ to account for inflation and different prices across countries, highlight the tremendous variation in how much is spent on health around the world. Disparate spending levels also exist within World Bank income groups. In 2014, health spending across low-income countries was $120 per capita, but range from $33 (Somalia) to $347 (Uganda). Spending per capita across lower-middle-income countries was $267, but ranged from $92 (Bangladesh) to $791 (Tunisia), while spending per capita in upper-middle-income countries was $914, but ranged from $228 (Angola) to $1980 (Maldives). Finally, health spending per capita was $5221 in high-income countries, and ranged from $853 (Seychelles) to $9237 (USA). Geographic variation is also present when examined using GBD super regions.

**Table 1:** Health spending by source, 2014

	**Total health spending per capita ($)**	**Total health expenditure per gross domestic product (%)**	**Domestic government health spending per total health spending (%)**	**Prepaid private spending per total health spending (%)**	**Out-of-pocket spending per total health spending (%)**	**Development assistance for health per total health spending (%)**	**Annualised rate of change in total health spending per capita, 1995–2014 (%)**
**Global**							
Total	1279 (33 to 9237)	8·3% (1·9 to 39·3)	59·2% (0·0 to 95·5)	17·4% (0·0 to 64·8)	22·8% (2·4 to 76·6)	0·6% (0·0 to 92·3)	3·3% (−3·0 to 19·7)
**Income group**							
High income	5221 (853 to 9237)	11·7% (2·2 to 16·6)	63·4% (42·4 to 93·9)	22·7% (0·0 to 38·8)	13·9% (2·4 to 55·7)	0·0% (0·0 to 0·1)	3·0% (−1·1 to 7·6)
Upper-middle income	914 (228 to 1980)	5·9% (2·3 to 17·2)	57·2% (19·4 to 95·5)	8·7% (0·0 to 44·2)	33·8% (4·4 to 74·2)	0·3% (0·0 to 23·2)	5·9% (−3·0 to 17·0)
Lower-middle income	267 (92 to 791)	4·3% (1·9 to 16·1)	35·9% (0·0 to 87·2)	3·1% (0·0 to 10·2)	58·0% (2·8 to 76·6)	3·0% (0·2 to 92·3)	5·0% (−1·4 to 9·4)
Low income	120 (33 to 347)	7·3% (3·6 to 39·3)	18·0% (0·0 to 48·5)	17·2% (0·0 to 64·8)	29·1% (7·8 to 54·1)	35·7% (12·9 to 92·2)	4·6% (−3·0 to 19·7)
**GBD super region**							
Central Europe, eastern Europe, and central Asia	1364 (200 to 2845)	6·7% (2·3 to 10·3)	58·5% (19·4 to 84·8)	2·8% (0·0 to 18·9)	38·5% (11·2 to 74·2)	0·3% (0·0 to 13·7)	4·9% (1·4–9·6)
Global Burden of Disease high income	5460 (1322 to 9237)	12·3% (2·6 to 16·6)	62·8 %(42·4 to 93·9)	23·4% (0·0 to 38·8)	13·8% (5·3 to 55·7)	0·0% (0·0 to 0·0)	2·9%(−0·6 to 7·6)
Latin America and Caribbean	1082 (154 to 1996)	7·3% (4·3 to 11·1)	51·6% (0·0 to 95·5)	16·1% (0·0 to 29·6)	31·7% (4·4 to 64·3)	0·7% (0·0 to 40·8)	3·3% (−1·8 to 8·5)
North Africa and Middle East	870 (159 to 2663)	5·2% (2·2 to 9·7)	60·1% (14·3 to 91·8)	4·3% (0·0 to 14·9)	34·9% (5·9 to 76·6)	0·7% (0·0 to 30·9)	4·9% (−1·4 to 9·0)
South Asia	223 (92 to 279)	4·2% (2·7 to 5·8)	31·0% (22·7 to 70·7)	2·6% (0·0 to 6·1)	64·7% (25·1 to 65·6)	1·7% (0·7 to 17·8)	5·8% (2·0 to 6·4)
Southeast Asia, east Asia, and Oceania	588 (105 to 1980)	4·8% (1·9 to 17·2)	58·6% (0·0 to 93·6)	5·2% (0·0 to 10·2)	35·7% (2·4 to 65·4)	0·5% (0·0 to 92·3)	8·9% (−3·0 to 11·0)
Sub-Saharan Africa	218 (33 to 1411)	5·9% (3·0 to 39·3)	33·5% (0·0 to 80·7)	20·8% (0·0 to 64·8)	29·2% (5·1 to 70·1)	16·6% (0·1 to 92·2)	3·2% (−3·0 to 19·7)
**Country**							
Afghanistan	159	9·7%	15·0%	0·0%	54·1%	30·9%	5·5%
Albania	642	5·9%	48·3%	0·0%	49·8%	1·9%	4·2%
Algeria	1004	7·2%	72·7%	0·7%	26·5%	0·0%	6·0%
Andorra	5723	8·1%	78·0%	6·0%	15·9%	0·0%	2·5%
Angola	228	3·0%	70·0%	0·0%	26·6%	3·4%	1·5%%
Antigua and Barbuda	1213	5·5%	68·3%	8·0%	23·7%	0·0%	3·0%
Argentina	1322	4·8%	55·8%	13·2%	30·9%	0·0%	–0·6%
Armenia	395	4·5%	40·6%	3·0%	52·8%	3·6%	4·9%
Australia	4032	9·0%	70·4%	9·9%	19·7%	0·0%	3·3%
Austria	5471	11·2%	78·0%	5·8%	16·2%	0·0%	2·6%
Azerbaijan	1030	5·9%	20·9%	4·3%	74·2%	0·6%	9·6%
Bahrain	2258	4·8%	65·3%	10·6%	24·1%	0·0%	2·2%
Bangladesh	92	2·9%	22·7%	0·0%	65·6%	11·7%	3·2%
Barbados	1116	7·5%	63·5%	6·6%	29·9%	0·0%	2·0%
Belarus	1093	5·6%	66·9%	0·1%	32·6%	0·4%	4·0%
Belgium	4751	10·6%	77·9%	4·3%	17·8%	0·0%	3·3%
Belize	503	5·8%	64·7%	9·5%	23·0%	2·9%	2·8%
Benin	105	5·1%	35·0%	0·0%	35·5%	29·6%	2·0%
Bhutan	279	3·6%	70·7%	0·0%	25·1%	4·2%	4·0%
Bolivia	404	6·3%	70·2%	3·4%	23·1%	3·3%	5·2%
Bosnia and Herzegovina	992	9·5%	70·0%	0·0%	28·0%	2·0%	8·8%
Botswana	903	5·5%	49·9%	35·0%	5·1%	10·0%	5·1%
Brazil	1357	8·3%	45·9%	28·5%	25·5%	0·1%	3·3%
Brunei	1811	2·6%	93·9%	0·1%	6·0%	0·0%	–0·4%
Bulgaria	1490	8·4%	54·7%	0·9%	44·3%	0·2%	6·3%
Burkina Faso	83	5·0%	35·8%	0·0%	38·6%	25·6%	2·8%
Burundi	65	8·3%	23·7%	0·0%	19·1%	57·2%	3·2%
Cambodia	209	6·4%	14·2%	0·0%	65·4%	20·4%	4·1%
Cameroon	116	4·0%	17·0%	3·5%	68·5%	10·9%	1·3%
Canada	4576	10·3%	72·1%	14·1%	13·8%	0·0%	2·4%
Cape Verde	318	4·8%	58·4%	0·1%	22·2%	19·2%	4·1%
Central African Republic	35	5·7%	9·0%	0·0%	34·2%	56·7%	–1·3%
Chad	89	3·8%	48·5%	1·3%	37·2%	12·9%	0·6%
Chile	1780	7·8%	49·5%	19·0%	31·5%	0·0%	4·1%
China	697	5·1%	60·3%	5·0%	34·6%	0·0%	10·4%
Colombia	975	7·2%	71·9%	9·5%	15·3%	3·2%	2·7%
Comoros	111	7·1%	22·1%	20·1%	42·8%	14·9%	–1·2%
Costa Rica	1418	9·3%	73·1%	1·8%	25·0%	0·0%	4·6%
Côte d'Ivoire	179	5·3%	22·1%	8·2%	54·6%	15·1%	0·2%
Croatia	1734	7·8%	81·9%	6·9%	11·2%	0·0%	3·5%
Cuba	1706	11·1%	95·5%	0·0%	4·4%	0·2%	8·5%
Cyprus	2019	7·2%	46·0%	4·4%	49·6%	0·0%	3·0%
Czech Republic	2384	7·4%	84·8%	0·8%	14·4%	0·0%	2·8%
DR Congo	46	4·5%	21·3%	0·0%	37·4%	41·3%	2·9%
Denmark	5075	10·8%	84·8%	1·9%	13·4%	0·0%	2·8%
Djibouti	357	10·9%	58·3%	0·0%	34·6%	7·1%	5·3%
Dominica	599	5·5%	68·7%	3·0%	28·3%	0·0%	1·1%
Dominican Republic	601	4·4%	63·4%	11·4%	21·0%	4·2%	3·0%
Ecuador	1071	9·2%	48·8%	2·2%	48·5%	0·5%	8·0%
Egypt	581	5·4%	39·9%	1·5%	58·3%	0·2%	5·5%
El Salvador	567	6·8%	64·7%	4·9%	28·8%	1·6%	3·3%
Equatorial Guinea	1411	3·7%	79·2%	0·0%	20·7%	0·1%	17·0%
Eritrea	59	5·1%	23·4%	0·0%	35·2%	41·4%	–1·1%
Estonia	1830	6·4%	79·0%	0·3%	20·8%	0·0%	5·1%
Ethiopia	85	5·5%	26·9%	0·0%	28·4%	44·7%	7·6%
Federated States of Micronesia	490	16·1%	0·0%	0·0%	7·7%	92·3%	2·9%
Fiji	399	4·5%	63·8%	7·5%	23·0%	5·7%	3·3%
Finland	3935	9·3%	78·0%	3·1%	18·9%	0·0%	3·1%
France	4589	11·3%	79·9%	13·6%	6·5%	0·0%	2·0%
Gabon	612	3·4%	67·4%	8·8%	22·0%	1·8%	0·0%
Georgia	700	7·3%	19·4%	18·9%	59·1%	2·6%	9·3%
Germany	5356	11·2%	77·3%	9·4%	13·3%	0·0%	2·6%
Ghana	146	3·5%	52·8%	3·1%	27·1%	17·0%	4·3%
Greece	2170	8·1%	61·7%	3·4%	34·9%	0·0%	0·9%
Grenada	737	6·1%	46·6%	2·0%	51·2%	0·2%	2·0%
Guatemala	466	6·2%	36·9%	8·2%	52·1%	2·8%	3·9%
Guinea	101	7·4%	20·4%	0·0%	34·5%	45·1%	3·5%
Guinea-Bissau	77	5·3%	6·0%	0·0%	52·1%	41·9%	–3·0%
Guyana	438	5·4%	53·5%	2·9%	36·5%	7·1%	3·2%
Haiti	154	8·9%	0·0%	29·6%	29·6%	40·8%	–1·8%
Honduras	420	8·8%	47·2%	5·0%	43·3%	4·6%	4·4%
Hungary	1855	7·2%	68·1%	4·4%	27·5%	0·0%	2·5%
Iceland	3959	8·7%	82·3%	0·0%	17·7%	0·0%	2·3%
India	253	4·5%	31·3%	2·4%	65·6%	0·7%	6·4%
Indonesia	265	2·5%	42·7%	2·7%	53·5%	1·1%	5·2%
Iran	1073	6·5%	43·8%	5·3%	50·8%	0·0%	6·2%
Iraq	828	5·7%	58·2%	3·0%	38·4%	0·5%	7·5%
Ireland	4006	7·6%	67·6%	14·3%	18·1%	0·0%	4·6%
Israel	2722	7·7%	61·5%	11·2%	27·3%	0·0%	1·9%
Italy	3311	9·0%	77·4%	0·9%	21·7%	0·0%	1·8%
Jamaica	477	5·4%	50·5%	19·4%	27·8%	2·3%	1·7%
Japan	3816	10·2%	83·6%	2·4%	13·9%	0·0%	3·0%
Jordan	839	7·4%	66·8%	8·0%	21·1%	4·1%	2·7%
Kazakhstan	1143	4·3%	54·4%	0·0%	45·3%	0·3%	5·8%
Kenya	197	6·4%	37·8%	3·8%	23·4%	35·0%	3·4%
Kiribati	168	9·6%	79·3%	0·0%	2·8%	17·9%	0·3%
Kuwait	2075	3·0%	85·9%	1·3%	12·7%	0·0%	–1·1%
Kyrgyzstan	236	6·9%	47·7%	1·3%	37·3%	13·7%	3·1%
Laos	113	2·0%	28·3%	0·4%	36·6%	34·7%	1·8%
Latvia	1427	5·9%	63·2%	1·7%	35·1%	0·0%	5·5%
Lebanon	1060	6·4%	47·6%	14·9%	36·4%	1·1%	–1·2%
Lesotho	319	11·6%	63·4%	0·3%	15·0%	21·3%	6·1%
Liberia	345	39·3%	0·0%	0·0%	7·8%	92·2%	19·7%
Libya	751	5·0%	73·5%	0·0%	26·5%	0·0%	0·0%
Lithuania	1830	6·5%	67·9%	0·8%	31·3%	0·0%	7·2%
Luxembourg	7105	6·9%	83·9%	5·5%	10·6%	0·0%	3·2%
Macedonia	887	6·5%	63·1%	0·0%	36·6%	0·3%	1·4%
Madagascar	52	3·7%	29·5%	0·0%	34·3%	36·2%	–0·4%
Malawi	148	12·9%	33·5%	14·0%	9·3%	43·1%	5·5%
Malaysia	1047	4·1%	56·0%	8·1%	35·8%	0·0%	5·1%
Maldives	1980	13·5%	79·4%	2·0%	18·5%	0·0%	11·0%
Mali	162	7·4%	22·0%	10·9%	43·6%	23·5%	4·0%
Malta	3058	9·7%	69·2%	2·0%	28·9%	0·0%	5·5%
Marshall Islands	599	17·2%	62·9%	2·1%	11·8%	23·2%	–3·0%
Mauritania	153	3·7%	44·5%	1·4%	44·7%	9·3%	0·7%
Mauritius	880	4·6%	50·8%	0·7%	48·0%	0·4%	4·8%
Mexico	1088	6·3%	51·7%	4·2%	44·0%	0·1%	2·9%
Moldova	527	10·3%	47·2%	8·2%	38·3%	6·3%	3·4%
Mongolia	575	4·7%	51·4%	0·9%	41·9%	5·8%	8·4%
Montenegro	1015	6·6%	55·3%	2·7%	41·4%	0·6%	3·4%
Morocco	505	5·9%	33·1%	7·6%	58·4%	0·9%	5·6%
Mozambique	92	7·8%	10·6%	0·6%	8·5%	80·2%	7·4%
Myanmar	121	2·5%	36·2%	0·0%	45·6%	18·2%	9·4%
Namibia	936	9·3%	53·5%	31·2%	6·9%	8·4%	5·2%
Nepal	138	5·8%	28·6%	5·9%	47·7%	17·8%	3·1%
Netherlands	5234	10·7%	88·4%	6·3%	5·3%	0·0%	3·9%
New Zealand	4050	11·0%	82·3%	6·6%	11·0%	0·0%	4·2%
Nicaragua	450	9·1%	50·9%	3·8%	37·3%	8·0%	3·6%
Niger	66	6·7%	26·3%	0·0%	49·5%	24·2%	0·6%
Nigeria	225	3·7%	22·1%	0·8%	70·1%	7·0%	7·5%
Norway	6537	10·0%	83·1%	3·7%	13·2%	0·0%	3·3%
Oman	1467	3·5%	91·8%	2·3%	5·9%	0·0%	1·6%
Pakistan	132	2·7%	32·1%	6·1%	55·4%	6·4%	2·0%
Panama	1743	8·0%	72·5%	4·5%	22·3%	0·8%	4·8%
Papua New Guinea	108	4·4%	60·1%	3·9%	10·1%	25·9%	3·5%
Paraguay	863	9·8%	45·6%	4·6%	49·3%	0·5%	4·7%
Peru	626	5·2%	63·3%	6·3%	30·0%	0·4%	4·4%
Philippines	330	4·7%	33·6%	10·2%	54·3%	1·9%	4·4%
Poland	1629	6·3%	71·4%	5·0%	23·6%	0·0%	5·0%
Portugal	2697	9·3%	66·6%	5·9%	27·6%	0·0%	3·3%
Qatar	2663	2·2%	85·7%	7·4%	6·9%	0·0%	0·7%
Congo (Brazzaville)	312	5·2%	80·7%	0·3%	17·4%	1·7%	4·5%
Romania	1077	5·5%	79·1%	0·4%	18·9%	1·6%	7·2%
Russia	1877	7·1%	51·8%	2·8%	45·5%	0·0%	5·4%
Rwanda	158	9·4%	0·0%	22·4%	22·6%	55·0%	7·9%
Saint Lucia	755	6·7%	49·2%	0·8%	45·6%	4·4%	1·4%
Saint Vincent and the Grenadines	917	8·8%	46·1%	2·0%	48·2%	3·6%	3·4%
Samoa	365	7·2%	87·2%	0·0%	5·9%	6·9%	4·5%
São Tomé and Príncipe	251	7·9%	31·1%	8·0%	11·9%	49·0%	1·9%
Saudi Arabia	2320	4·4%	78·7%	6·2%	15·1%	0·0%	5·0%
Senegal	121	5·2%	39·4%	0·0%	33·8%	26·9%	2·1%
Serbia	1392	10·3%	62·5%	0·3%	37·0%	0·1%	6·9%
Seychelles	853	3·3%	93·6%	4·0%	2·4%	0·1%	–0·2%
Sierra Leone	255	13·5%	5·1%	9·2%	50·1%	35·6%	3·0%
Singapore	3981	4·8%	42·4%	1·9%	55·7%	0·0%	6·5%
Slovakia	2203	7·7%	76·3%	0·0%	23·7%	0·0%	5·1%
Slovenia	2845	9·1%	73·2%	14·5%	12·3%	0·0%	3·5%
Solomon Islands	107	5·8%	67·0%	0·0%	4·0%	29·1%	2·7%
Somalia	33	6·9%	25·0%	1·2%	28·5%	45·2%	1·9%
South Africa	1172	8·9%	47·0%	44·2%	6·4%	2·4%	2·1%
South Korea	2507	7·1%	56·0%	6·6%	37·4%	0·0%	7·6%
South Sudan	94	3·6%	21·0%	0·0%	40·7%	38·3%	1·8%
Spain	3096	9·0%	71·1%	4·8%	24·1%	0·0%	2·6%
Sri Lanka	402	3·5%	54·5%	1·0%	42·3%	2·1%	5·2%
Sudan	334	8·3%	20·4%	0·9%	76·6%	2·2%	9·0%
Suriname	731	4·3%	67·6%	15·5%	15·2%	1·7%	2·0%
Swaziland	745	9·5%	66·6%	8·4%	10·0%	15·0%	4·7%
Sweden	5446	11·8%	85·1%	0·6%	14·2%	0·0%	4·0%
Switzerland	7831	12·8%	60·3%	15·2%	24·5%	0·0%	3·2%
Syria	562	3·4%	44·5%	3·3%	51·6%	0·6%	–1·4%
Tajikistan	200	7·3%	22·9%	8·7%	57·9%	10·6%	7·3%
Tanzania	166	6·4%	20·3%	17·1%	20·2%	42·4%	7·2%
Thailand	633	4·1%	78·7%	8·6%	12·1%	0·7%	3·7%
The Bahamas	1996	7·7%	45·9%	24·9%	29·2%	0·0%	1·2%
The Gambia	151	9·2%	47·4%	0·0%	13·6%	39·0%	5·8%
Timor-Leste	105	1·9%	51·6%	0·0%	7·4%	41·0%	8·3%
Togo	81	5·5%	29·7%	7·8%	44·3%	18·3%	1·9%
Tonga	253	5·3%	69·5%	0·4%	11·7%	18·5%	1·7%
Trinidad and Tobago	1823	5·8%	54·5%	6·8%	38·7%	0·0%	6·2%
Tunisia	791	6·9%	57·2%	4·5%	38·1%	0·2%	3·5%
Turkey	1040	5·3%	78·4%	3·5%	18·0%	0·1%	6·6%
Turkmenistan	396	2·3%	59·2%	8·7%	31·6%	0·6%	5·3%
Uganda	347	18·1%	0·9%	64·8%	16·4%	18·0%	9·3%
Ukraine	659	7·0%	51·3%	0·9%	46·8%	0·9%	2·9%
United Arab Emirates	2561	3·6%	72·3%	9·9%	17·8%	0·0%	–0·4%
UK	3749	9·1%	83·1%	7·1%	9·7%	0·0%	3·4%
USA	9237	16·6%	49·8%	38·8%	11·4%	0·0%	2·9%
Uruguay	1837	8·6%	71·2%	13·2%	15·6%	0·0%	2·9%
Uzbekistan	397	5·9%	51·9%	2·6%	43·7%	1·7%	3·6%
Vanuatu	149	5·4%	56·7%	0·0%	5·4%	37·9%	4·1%
Venezuela	1010	5·3%	29·3%	6·3%	64·3%	0·0%	1·0%
Vietnam	398	7·0%	53·0%	6·9%	37·4%	2·7%	7·0%
Yemen	233	5·8%	14·3%	1·7%	74·7%	9·3%	3·0%
Zambia	216	5·4%	32·6%	0·0%	27·7%	39·7%	4·8%

Data in parenthesis are range within aggregate. Currency are 2015 purchasing power parity US$. GBD=Global Burden of Disease.

Table 1 also highlights the sources of health financing in 2014. Globally, governments provided 59·2% of health spending, while 17·4% of total health financing was prepaid private, 22·8% was OOP, and 0·6% was development assistance for health. In low-income countries, health spending was predominately financed by development assistance for health and OOP, constituting 35·7% and 29·1% of total health spending, respectively. However, the share of health financing sourced from OOP is largest in lower-middle-income countries, at 58·0%, where development assistance for health makes up a smaller share of total health spending than in low-income countries. Governments in upper-middle-income and high-income countries, by contrast, finance the bulk of health care and related activities, at 57·2% and 63·4%, respectively. Across all income groups, prepaid private health spending remained quite low. Within income groups, and also GBD super regions, the composition of sources financing the health system varied dramatically.

### Health spending trends across time

**Figure 1:**
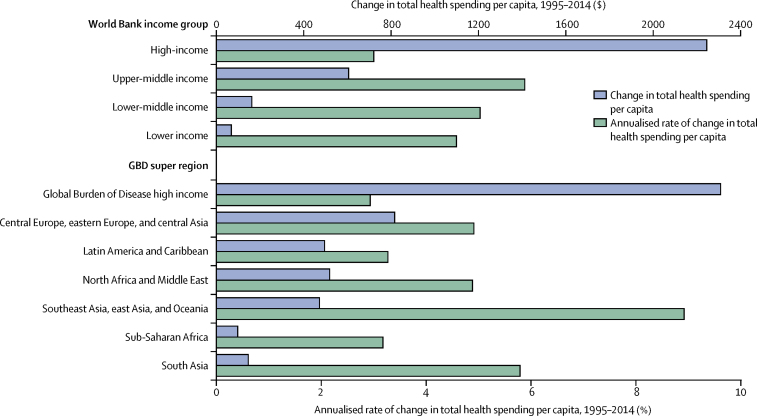
Changes in health spending by income group and Global Burden of Disease (GBD) super region, 1995–2014 Currency reported in 2015 purchasing power parity adjusted $.

Figure 1 highlights how total health spending per capita has changed between 1995 and 2014. Upper-middle and lower-middle-income country groups have increased per capita health spending the fastest, with annualised growth rates of 5·9% and 5·0%, respectively. Over the course of 20 years, this has led to a near tripling of health spending per capita in upper-middle-income countries, from $309 to $914 per capita. Spending in low-income countries grew at 4·6%, while the slowest growth was observed in high-income countries, which grew collectively at 3·0% per year. Despite this slower rate, the largest health spending increases in terms of dollar per capita increase was in high-income countries, which added $2244 per capita in spending. Upper-income and lower-income countries added $605 and $162 per capita respectively. Low-income countries, which spent very little in 1995, increased health spending by $69 per capita between 1995 and 2014. Liberia, Equatorial Guinea, and Maldives had the fastest per capita growth rates between 1995 and 2014 (table 1).

For high-income and middle-income countries, the growth in spending was driven by increases in government spending. For example, in high-income countries, 64·5% of the $2244 increase was due to increases in government spending. Conversely, the growth in low-income countries was driven by increases in development assistance for health (51·0% of absolute increase).

Although we observed increases in spending per capita between 1995 and 2014 in 170 of 184 countries, the multivariate penalised spline analysis shows that when controlling on GDP per capita, there was essentially no change in health spending across time at the median health spending per capita value (appendix p 36 illustrates this null finding).

### Health spending trends and economic development

**Figure 2:**
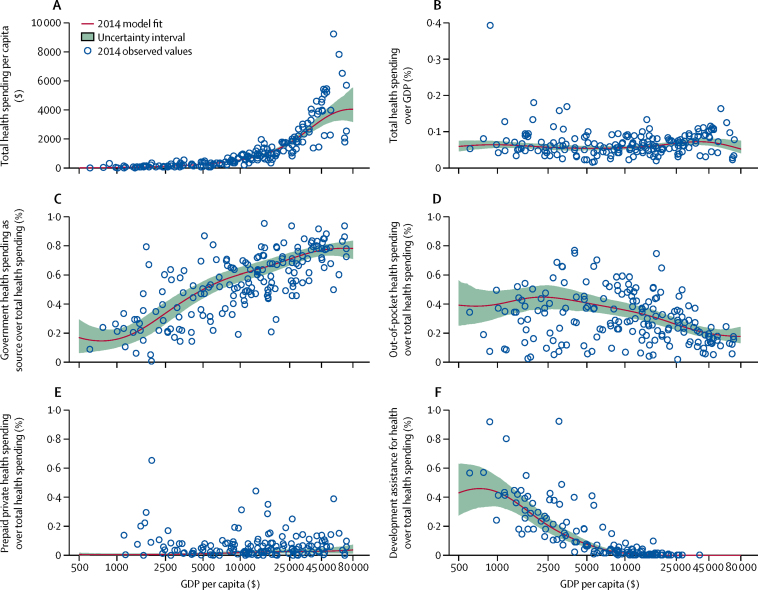
Health spending by source (total health spending [A, B], government health spending [C]; out-of-pocket health spending [D]; prepaid private health spending [E], and development assistance for health [F]), 2014 Health spending relative to gross domestic product (GDP) per capita (reported in 2015 purchasing-power-parity [PPP] adjusted $). Although all countries and years of data were used for this analysis, the trend line reflects the 2014 model fit.

Although time does not seem to be associated with health spending per capita, our trend analyses confirm the relationship between economic development and health financing—total health spending increases with economic development, while the share of OOP financing decreases. These associations are represented in figure 2. Across countries, growth in GDP per capita was associated with exponential growth in total health spending per capita (figure 2A; appendix p 40; p<0·000).

Figure 2B shows that when measured as a share of GDP, there is not a robust relationship connecting economic development and spending. The estimated fit is nearly flat and there are countries with similar GDP per capita with disparate spending levels.

Figure 2C and 2D show that the share of health spending from the government increases with economic development, while the share that is OOP decreases. Prepaid private spending as a share of total spending is very small, on average, across all levels of economic development (figure 2E), while the share of health spending that is development assistance for health increases at the very lowest levels of GDP per capita and peaks at GDP per capita of $801 (figure 2F).

**Figure 3:**
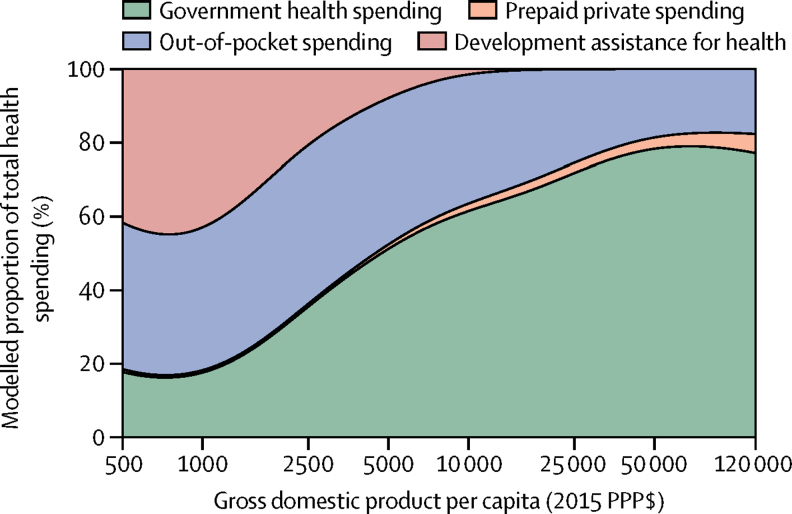
Composition of health-care spending by source, 2014 PPP=purchasing-power-parity.

Figure 3 shows how the fraction of total health spending from governments, prepaid private, OOP, and development assistance for health evolve, on average, with economic development. On average, the proportion of total health spending sourced from governments rise as GDP per capita increases. At the 80th wealth percentile (GDP per capita of $27 617), trend analysis estimates that health spending is financed by the governments (72·2%), with only 24·9% sourced from OOP. At the 20th wealth percentile (GDP per capita of $2267), trend analysis estimates that OOP financing is 44·8% of total spending, while development assistance for health is 23·1%.

### Deviation from the health financing trends

Table 2 measures health spending relative to the modelled amount, which is based on country-specific and year-specific GDP per capita. The first column reports total health spending for 2014, which, as figure 2A shows, increases exponentially with GDP per capita. Countries such as Afghanistan, Brazil, France, Liberia, Uganda, and the USA spent more in 2014 than the modelled average, based on each country's GDP per capita. On the other hand, countries such as Timor-Leste, Laos, and Turkmenistan spend 33·0%, 34·7%, and 35·6% of the expected amount.

**Table 2:** Health spending relative to expected health spending, 2014

	**Total health spending relative to modelled total health spending (%)**	**Share of total health spending that is from the government, relative to the modelled share (%)**	**Share of total health spending that is prepaid private, relative to the modelled share (%)**	**Share of total health spending that is out-of-pocket, relative to the modelled share (%)**	**Share of total health spending that is development assistance, relative to the modelled share (%)**
Afghanistan	153·8%	64·0%	0·3%	123·7%	96·3%
Albania	99·5%	78·6%	0·6%	141·5%	170·3%
Algeria	118·0%	113·8%	30·3%	80·0%	8·8%
Andorra	141·7%	99·6%	167·6%	88·2%	0·0%
Angola	52·0%	122·2%	0·0%	70·3%	109·7%
Antigua and Barbuda	81·1%	98·7%	293·5%	84·8%	9·6%
Argentina	65·2%	77·2%	475·9%	124·3%	30·8%
Armenia	78·7%	69·0%	158·5%	142·9%	162·4%
Australia	121·3%	91·4%	333·2%	98·6%	0·0%
Austria	154·6%	100·6%	191·4%	83·2%	0·0%
Azerbaijan	91·3%	31·4%	164·4%	242·1%	263·9%
Bahrain	66·0%	84·5%	354·1%	121·9%	0·0%
Bangladesh	49·2%	56·7%	0·0%	149·9%	76·7%
Barbados	120·6%	98·2%	264·0%	92·2%	0·0%
Belarus	84·3%	98·7%	5·5%	111·0%	232·0%
Belgium	142·9%	101·2%	144·0%	88·9%	0·0%
Belize	100·1%	109·7%	498·3%	62·2%	131·1%
Benin	82·8%	120·8%	0·0%	79·3%	115·9%
Bhutan	62·9%	123·3%	0·0%	66·3%	137·8%
Bolivia	110·7%	128·8%	225·1%	59·0%	68·2%
Bosnia and Herzegovina	162·4%	114·9%	0·0%	78·6%	154·6%
Botswana	86·4%	75·9%	1352·6%	16·4%	3595·7%
Brazil	131·4%	70·0%	1104·9%	81·0%	33·1%
Brunei	45·0%	119·9%	2·8%	33·1%	0·0%
Bulgaria	130·6%	82·1%	33·2%	145·0%	72·0%
Burkina Faso	80·1%	152·7%	0·0%	88·2%	80·0%
Burundi	119·3%	161·9%	0·0%	49·3%	124·5%
Cambodia	109·2%	35·0%	0·0%	149·9%	137·0%
Cameroon	67·3%	45·1%	404·5%	155·1%	63·6%
Canada	138·7%	93·8%	475·0%	68·8%	0·0%
Cape Verde	83·0%	105·6%	9·4%	57·3%	446·0%
Central African Republic	77·4%	59·1%	0·0%	88·4%	125·3%
Chad	63·1%	151·3%	168·8%	83·1%	57·9%
Chile	111·6%	70·8%	693·3%	115·4%	3·3%
China	83·9%	94·7%	208·7%	103·9%	5·9%
Colombia	118·8%	113·1%	394·1%	46·0%	586·2%
Comoros	111·9%	99·1%	2797·1%	98·6%	44·5%
Congo	90·7%	150·8%	17·6%	44·0%	31·0%
Costa Rica	147·8%	112·5%	71·1%	78·0%	8·3%
Côte d'Ivoire	90·7%	53·6%	869·3%	125·5%	105·6%
Croatia	113·0%	117·9%	253·3%	40·5%	0·0%
Cuba	177·8%	146·9%	0·0%	13·7%	44·9%
Cyprus	98·4%	63·6%	157·4%	200·1%	0·0%
Czech Republic	98·4%	114·6%	28·1%	62·2%	0·0%
Congo (Brazzaville)	66·9%	134·1%	0·0%	93·5%	95·1%
Denmark	147·3%	109·7%	62·3%	67·9%	0·0%
Djibouti	186·7%	143·9%	0·0%	79·4%	47·5%
Dominica	92·9%	111·7%	135·8%	80·5%	3·5%
Dominican Republic	72·4%	99·4%	472·1%	63·1%	789·2%
Ecuador	153·4%	78·5%	96·8%	139·9%	59·0%
Egypt	91·0%	65·1%	68·6%	165·3%	20·8%
El Salvador	117·8%	110·7%	262·7%	77·3%	66·3%
Equatorial Guinea	48·7%	104·6%	0·0%	96·9%	480·8%
Eritrea	78·2%	136·8%	0·0%	86·0%	100·3%
Estonia	86·0%	108·6%	9·6%	85·0%	0·0%
Ethiopia	87·6%	122·7%	0·0%	65·6%	131·3%
Federated States of Micronesia	273·0%	0·0%	0·0%	17·5%	566·3%
Fiji	77·8%	107·8%	390·3%	62·4%	277·3%
Finland	124·2%	101·9%	105·6%	92·2%	0·0%
France	150·0%	104·9%	467·6%	31·1%	0·0%
Gabon	52·9%	101·1%	332·0%	72·4%	853·0%
Georgia	126·5%	32·3%	941·1%	162·8%	151·8%
Germany	154·4%	100·0%	311·0%	67·6%	0·0%
Ghana	61·0%	114·5%	286·3%	64·4%	159·6%
Greece	111·0%	85·8%	124·1%	138·0%	0·0%
Grenada	102·0%	74·5%	88·4%	148·8%	27·3%
Guatemala	108·6%	64·7%	477·2%	137·2%	85·3%
Guinea	115·4%	103·8%	0·0%	81·5%	120·9%
Guinea-Bissau	83·7%	29·2%	0·0%	121·8%	116·5%
Guyana	92·9%	92·0%	155·8%	97·7%	275·7%
Haiti	142·4%	0·0%	4032·0%	67·1%	133·3%
Honduras	153·3%	96·0%	413·0%	105·1%	54·1%
Hungary	99·8%	95·4%	159·5%	106·5%	0·0%
Iceland	118·2%	106·8%	0·0%	88·9%	0·0%
India	77·6%	59·5%	175·1%	164·5%	10·8%
Indonesia	42·5%	69·8%	124·6%	151·0%	93·9%
Iran	102·6%	66·6%	204·4%	162·2%	15·6%
Iraq	92·8%	90·4%	121·4%	117·3%	105·0%
Ireland	108·8%	86·9%	457·8%	94·9%	0·0%
Israel	101·9%	82·1%	393·6%	123·1%	0·0%
Italy	119·0%	102·7%	32·4%	99·7%	0·0%
Jamaica	92·9%	85·4%	1005·4%	75·5%	112·7%
Japan	134·4%	110·7%	84·7%	64·6%	0·0%
Jordan	124·4%	107·9%	358·9%	60·6%	422·4%
Kazakhstan	60·0%	75·9%	1·2%	177·3%	649·7%
Kenya	108·6%	96·8%	428·7%	53·2%	217·6%
Kiribati	154·9%	323·3%	0·0%	6·4%	58·3%
Kuwait	51·6%	109·7%	37·6%	70·3%	0·0%
Kyrgyzstan	117·6%	114·4%	139·3%	86·0%	98·0%
Laos	34·7%	53·7%	30·4%	91·8%	570·8%
Latvia	82·8%	89·5%	61·3%	132·4%	0·0%
Lebanon	100·1%	72·2%	574·1%	116·6%	422·6%
Lesotho	194·8%	175·0%	30·2%	33·8%	115·3%
Liberia	575·9%	0·0%	0·0%	20·0%	203·9%
Libya	80·2%	113·5%	0·2%	81·9%	8·3%
Lithuania	89·1%	93·8%	28·9%	126·3%	0·0%
Luxembourg	182·2%	108·5%	116·6%	59·1%	0·0%
Macedonia	106·8%	99·0%	0·0%	109·8%	63·2%
Madagascar	57·3%	146·3%	0·0%	80·4%	99·2%
Malawi	197·6%	197·2%	2043·1%	22·9%	104·1%
Malaysia	57·0%	78·6%	293·6%	138·4%	59·1%
Maldives	219·3%	123·2%	81·5%	56·8%	2·9%
Mali	121·0%	72·1%	1399·1%	97·2%	98·4%
Malta	130·7%	93·9%	70·4%	123·0%	0·0%
Marshall Islands	293·5%	149·1%	220·6%	27·2%	170·7%
Mauritania	64·0%	96·6%	128·7%	106·2%	87·6%
Mauritius	70·5%	75·4%	27·0%	161·6%	242·8%
Mexico	97·7%	77·9%	161·4%	142·9%	45·1%
Moldova	179·8%	93·3%	641·7%	94·2%	84·3%
Mongolia	78·7%	82·1%	37·5%	122·3%	752·2%
Montenegro	105·8%	85·0%	105·8%	129·1%	177·0%
Morocco	102·8%	56·4%	405·2%	157·4%	37·8%
Mozambique	119·0%	61·0%	93·7%	20·7%	196·6%
Myanmar	44·2%	73·7%	0·0%	110·7%	215·1%
Namibia	158·4%	88·2%	1495·1%	19·2%	600·0%
Nepal	95·9%	87·8%	732·5%	106·5%	81·7%
Netherlands	147·9%	114·1%	206·3%	27·3%	0·0%
New Zealand	145·5%	109·3%	231·1%	50·8%	0·0%
Nicaragua	157·9%	101·8%	306·9%	91·3%	101·0%
Niger	99·0%	168·1%	0·0%	124·4%	55·2%
Nigeria	65·6%	41·3%	53·1%	177·4%	127·5%
Norway	163·8%	106·1%	107·2%	72·6%	0·0%
Oman	46·3%	120·0%	76·9%	28·9%	0·0%
Pakistan	46·3%	64·2%	486·2%	135·6%	81·2%
Panama	116·5%	104·8%	163·9%	79·6%	758·5%
Papua New Guinea	73·5%	181·5%	488·4%	22·5%	121·6%
Paraguay	169·9%	77·2%	239·1%	133·7%	25·0%
Peru	87·6%	101·4%	276·0%	87·0%	48·7%
Philippines	80·9%	59·8%	614·9%	141·5%	50·7%
Poland	87·6%	100·0%	180·6%	91·6%	0·0%
Portugal	125·1%	91·4%	210·2%	113·5%	0·0%
Qatar	72·6%	112·2%	137·1%	37·8%	0·0%
Romania	84·1%	116·9%	15·1%	64·2%	1012·3%
Russia	98·5%	72·3%	100·0%	178·1%	0·0%
Rwanda	149·8%	0·0%	3072·2%	51·5%	174·6%
Saint Lucia	113·2%	79·7%	37·3%	130·3%	441·0%
Saint Vincent and the Grenadines	150·1%	75·7%	95·9%	135·3%	284·7%
Samoa	125·8%	173·2%	0·0%	14·5%	90·4%
Sao Tome and Principe	133·8%	77·6%	874·5%	27·2%	320·8%
Saudi Arabia	63·0%	101·2%	197·0%	79·4%	0·0%
Senegal	85·6%	122·7%	0·0%	75·3%	120·5%
Serbia	167·5%	98·1%	13·4%	111·2%	23·9%
Seychelles	46·5%	131·3%	143·3%	9·2%	181·7%
Sierra Leone	218·9%	19·2%	1235·0%	112·7%	125·6%
Singapore	98·1%	54·3%	47·7%	311·5%	0·0%
Slovakia	103·5%	104·9%	0·0%	97·1%	0·0%
Slovenia	120·2%	99·2%	516·1%	52·7%	0·0%
Solomon Islands	94·2%	260·3%	0·0%	9·0%	99·6%
Somalia	87·6%	142·7%	164·2%	72·2%	107·2%
South Africa	146·2%	74·0%	1850·3%	19·2%	411·3%
South Korea	93·9%	74·7%	233·0%	168·7%	0·0%
South Sudan	61·1%	61·1%	0·0%	91·1%	190·8%
Spain	118·4%	95·1%	170·8%	107·6%	0·0%
Sri Lanka	58·9%	88·0%	45·8%	121·5%	229·0%
Sudan	143·6%	44·8%	80·1%	180·9%	19·7%
Suriname	66·5%	102·1%	592·7%	49·1%	716·5%
Swaziland	164·8%	115·5%	473·5%	26·6%	519·4%
Sweden	160·3%	110·3%	20·8%	71·9%	0·0%
Switzerland	199·2%	77·1%	452·0%	133·2%	0·0%
Syria	53·1%	67·6%	127·1%	165·3%	216·1%
Tajikistan	123·6%	63·6%	1024·3%	130·3%	56·5%
Tanzania	107·3%	58·5%	2069·5%	45·2%	213·9%
Thailand	65·2%	120·9%	337·4%	37·7%	195·2%
The Bahamas	107·4%	64·3%	899·4%	113·4%	0·0%
The Gambia	145·7%	202·4%	0·0%	31·1%	121·6%
Timor-Leste	33·0%	99·1%	0·0%	18·6%	634·8%
Togo	86·0%	140·6%	1091·4%	103·0%	51·9%
Tonga	92·3%	141·4%	32·8%	28·4%	217·7%
Trinidad and Tobago	77·9%	74·0%	242·1%	164·9%	0·0%
Tunisia	117·2%	92·4%	202·5%	109·3%	18·0%
Turkey	80·2%	115·7%	130·9%	61·3%	35·7%
Turkmenistan	35·6%	89·2%	331·7%	102·5%	238·5%
Uganda	293·2%	3·3%	8624·8%	36·7%	64·8%
Ukraine	120·5%	85·8%	45·9%	128·7%	53·6%
United Arab Emirates	63·4%	92·4%	273·9%	98·6%	0·0%
UK	121·4%	109·0%	244·6%	46·9%	0·0%
USA	243·7%	63·8%	1211·9%	60·7%	0·0%
Uruguay	126·1%	103·3%	485·1%	55·2%	6·8%
Uzbekistan	102·4%	93·5%	165·1%	113·0%	41·6%
Vanuatu	90·0%	155·2%	0·0%	12·2%	208·0%
Venezuela	79·9%	43·4%	236·0%	217·4%	1·5%
Vietnam	121·9%	100·8%	494·6%	93·7%	44·3%
Yemen	100·2%	31·5%	161·1%	176·4%	83·5%
Zambia	93·7%	72·1%	0·0%	65·3%	351·9%

Columns 2 to 5 of table 2 report the share of health spending financed by different sources relative to the expected amount. The results show that some countries deviate substantially from average trends. For government health spending, Rwanda, Liberia, and Federated States of Micronesia stand out as countries that finance a smaller share than expected relative to respective GDP per capita. On the other hand, Kiribati, Solomon Islands, and The Gambia have governments that finance a greater share than expected. By contrast with the government health financing share, the OOP share of total health spending declines with economic development. Surrounding the modelled average, however, is a great deal of variation. Countries such as Afghanistan, Sudan, and Nigeria, spend more through OOP than expected. Conversely, countries such as Congo (Brazzaville), Algeria, South Africa, and France have a disproportionally smaller OOP share of total health spending.

Column 5 of table 2 reports the share of health spending that is from development partners, in this case measured relative to expected spending. As shown in figure 2F, development assistance for health as a share of total health spending actually increases with GDP per capita for the countries with the smallest GDP per capita; a number of countries, such as Niger, which has a very low GDP per capita, receive very little development assistance for health. Relative to the expected amount, countries such as Botswana, Romania, and Gabon finance a greater share of their health spending using development assistance funds than would otherwise be expected. On the other hand, countries such as Cameroon and Sudan finance their health spending with less development assistance funds than would be expected based on their GDP per capita.

**Figure 4:**
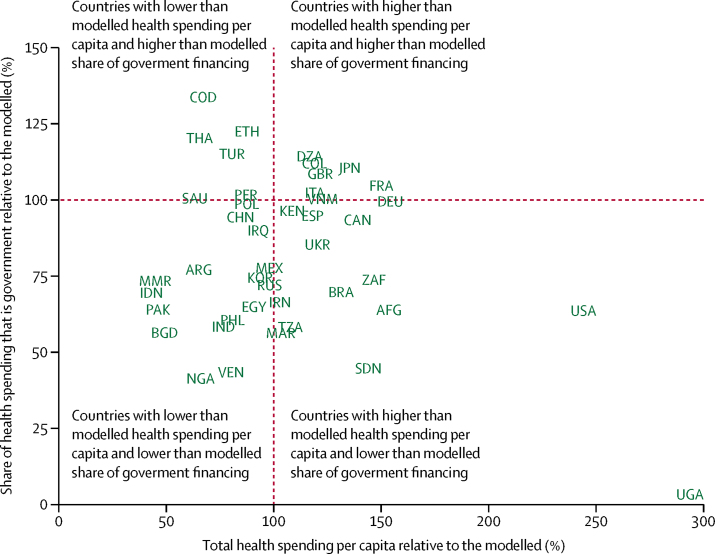
Observed government and total health spending relative to modelled spending, 2014 The figure shows the total health spending relative to modelled spending, and the share of health spending that is from the government relative to the modelled share. The vertical and horizontal red lines indicate where the observed spending is equal to the modelled spending (100%). Although all countries and years of data were used for this analysis, the deviations reflects the 2014 model fit. Only countries with a population higher than 30 million and 2014 data are included as points to avoid too many markers. A version with all countries included a dots is included in the appendix (p 68). AFG=Afghanistan. ARG=Argentina. BGD=Bangladesh. BRA=Brazil. CAN=Canada. CHN=China. COD=Democratic Republic of the Congo. COL=Colombia. DEU=Germany. DZA=Algeria. EGY=Egypt. ESP=Spain. ETH=Ethiopia. FRA=France. GBR=UK. IDN=Indonesia. IND=India. IRN=Iran. IRQ=Iraq. ITA=Italy. JPN=Japan. KEN=Kenya. KOR=South Korea. MAR=Morocco. MEX=Mexico. MMR=Myanmar. NGA=Nigeria. PAK=Pakistan. PER=Peru. PHL=Philippines. POL=Poland. RUS=Russia. SAU=Saudi Arabia. SDN=Sudan. THA=Thailand. TUR=Turkey. TZA=Tanzania. UGA=Uganda. UKR=Ukraine. USA=USA. VEN=Venezuela. VNM=Vietnam. ZAF=South Africa.

Figure 4 shows total health spending and the share that is government health spending, both relative to the modelled amount, based solely on GDP per capita. Countries such as Algeria and Japan have greater than modelled health spending per capita and greater than modelled share of financing from government. Countries such as Uganda and the USA have greater than modelled health spending per capita, but less than modelled share of financing from government. Conversely, countries such as Ethiopia and Thailand, have less health spending per capita than modelled, but higher than modelled share of financing from government, and countries such as India, Indonesia, and Philippines have less health spending per capita than modelled and lower than modelled share of financing from government.

### Trends in development assistance for health

**Figure 5:**
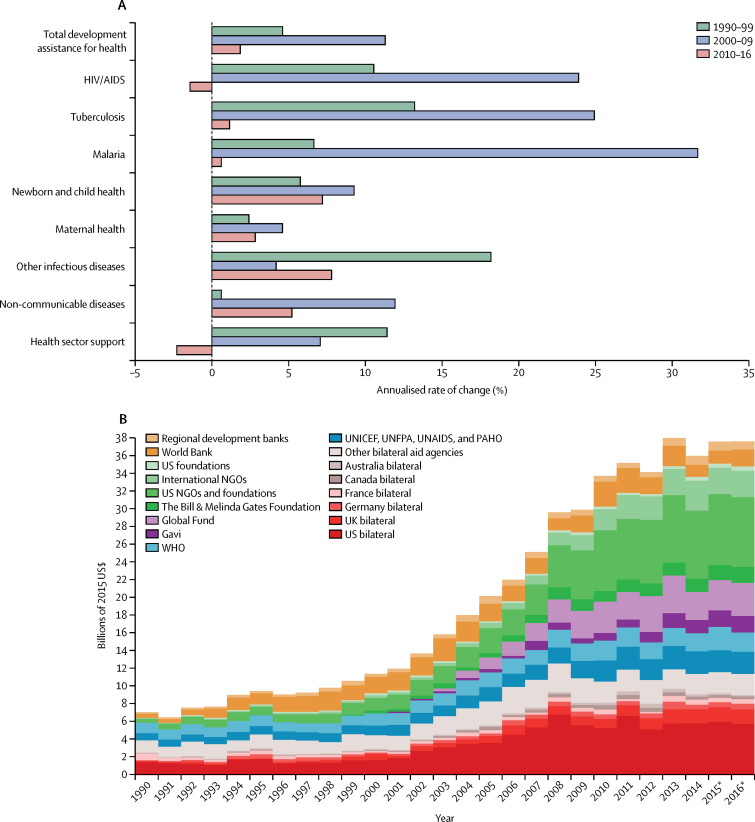
Changes in development assistance for health disbursements, 1990–2016 Development assistance for health as annualised growth rates (A) and disaggregated by channel (B). (A) Growth rates are shown for 1990–99, 2000–09, and 2010–16. (B) Estimates are shown from 1990 to 2016, all in billions of 2015 US$. World Bank includes the International Development Association and the International Bank for Reconstruction and Development; and regional development banks include the Inter-American Development Bank, the African Development Bank, and the Asian Development Bank. NGOs=non-governmental organisations. Global Fund=The Global Fund to Fight AIDS, Tuberculosis and Malaria. Gavi=Gavi, the Vaccine Alliance. UNICEF=United Nations Children's Fund. UNFPA=United Nations Population Fund. UNAIDS=Joint United Nations Programme on HIV/AIDS. PAHO=Pan American Health Organization. *Data for 2015 and 2016 are preliminary estimates based on budget data and estimation.

**Figure 6:**
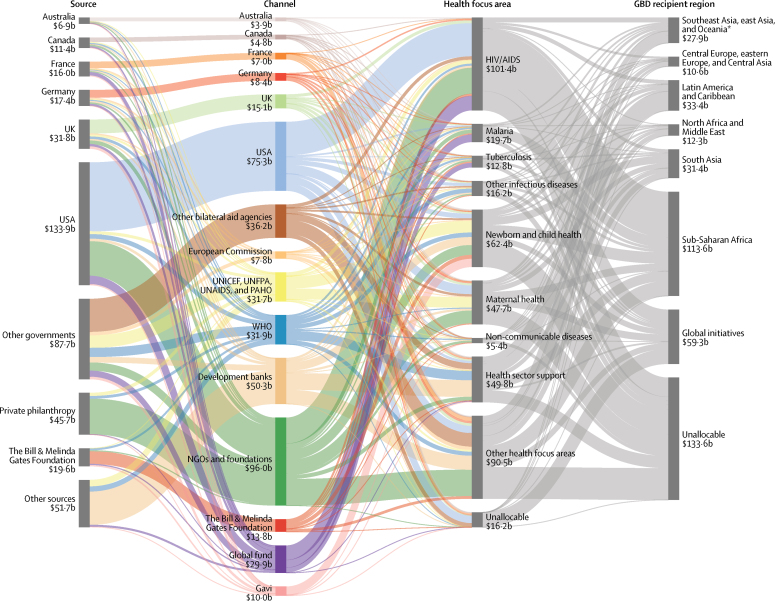
Flow of developmental assistance for health for all funds dispersed, 1995–2014 The figures shows the flow of development assistance for health from sources, through intermediary channels, to health focus areas and the region where the development assistance was ultimately received. Data are cumulative developmental assistance for health from 1995 to the end of 2014 in billions of 2015 US$. Each column disaggregates the total developmental assistance for health disbursed from 1995 through to 2014, which was US$423·0 billion. Funding sources are shown on the left, channels in the middle left, health focus areas on the middle right, and Global Burden of Disease (GBD) recipient super-regions are on the right. Private philanthropy includes corporate donations among other private philanthropy. Other sources include debt repayments and funds whose sources are unallocable. NGOs and foundations include non-governmental organisations and US foundations. UN Agencies include the UN Children's Fund, UN Population Fund, Joint UN Programme on HIV/AIDS, Pan American Health Organization, and WHO. Development banks include the World Bank International Development Association, the World Bank International Bank for Reconstruction and Development, the Inter-American Development Bank, the African Development Bank, and the Asian Development Bank. Other health focus areas correspond to developmental assistance for health for which we have project-level information but which is not identified as funding any of the health focus areas we tracked. Unallocable in terms of health focus area corresponds to developmental assistance for health for which we do not have project-level information and cannot parse across health focus areas. Latin America and the Caribbean includes Argentina, Chile, and Uruguay, which are now high-income countries when they were each middle-income countries. Southeast Asia, east Asia, and Oceania includes South Korea, which is also now a high-income country when it was a middle-income country. Unallocable in recipient region also corresponds to development assistance for health for which we do not have project-level information and thus, cannot parse across recipients. UNICEF=United Nations Children's Fund. UNFPA=United Nations Population Fund. UNAIDS=Joint United Nations Programme on HIV/AIDS. PAHO=Pan American Health Organization. Global Fund=The Global Fund to Fight AIDS, Tuberculosis and Malaria. Gavi=Gavi, the Vaccine Alliance.

Figure 5, Figure 6 highlight important trends related to development assistance for health, reported in 2015 US $. In 2016, total development assistance for health amounted to US$37·6 billion. Figure 5 shows the most recent growth rates in the disbursement of development assistance for health (figure 5A), and recent disbursements disaggregated by channel of assistance (figure 5B). In the 1990s, total development assistance for health grew at an annualised rate of 4·6%. During the first decade of the millennium, development assistance for health grew at an annualised rate of 11·3%, with especially large annualised growth for target areas associated with the Millennium Development Goals. Annualised growth between 2000 and 2009 was the highest for malaria, tuberculosis, and HIV/AIDS. Since 2010, total development assistance for health has grown at an annualised growth rate of 1·8%, with reductions in spending (−1·4%) for the largest health focus area, which is HIV/AIDS.

Between 1995 and 2014, $423·0 billion of development assistance for health was disbursed to low-income and middle-income countries (figure 6). 27·0% of development assistance for health went to sub-Saharan Africa, whereas 7·9% flowed to Latin America and the Caribbean. Across health focus areas, 24·0% of development assistance for health focused on HIV/AIDS, while child and maternal health projects received 14·8% and 11·3% of funding, respectively. The majority of the resources were provided by national treasuries, including the USA at 34·0% and the UK at 10·9%, of total development assistance for health in 2016. Major shares of development assistance for health also flowed through multilateral development agencies, such as the World Bank and WHO, which disbursed 5·1% and 5·8%, respectively, and private-public partnerships, such as the Global Fund and Gavi, who disbursed 9·9% and 4·9%, respectively, in 2016.

### Health spending by type of goods and service

**Figure 7:**
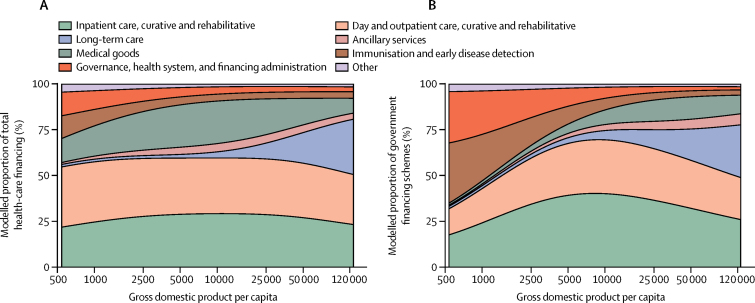
Composition of health spending by type of goods and services, 2014 The modelled proportion of total (A) and government (B) health spending across gross domestic product per capita by types of goods and services. Other health spending includes all other health spending that is not otherwise classified in this taxonomy. Spending on education and counselling programs, epidemiological surveillance, and disaster preparedness was excluded.

Figure 7 shows that spending by type of goods and service was relatively constant across different levels of economic development. At the median GDP per capita ($8346), 29·0% of total spending was on inpatient curative and rehabilitative care, 30·6% is on day and outpatient curative and rehabilitative care, and 23·5% is on medical goods, which include pharmaceuticals, in 2014 (figure 7A). These values drop marginally at the highest values of GDP per capita, where long-term care is more prominent.

Government spending by type of goods and service is very similar to total spending (figure 7B), although the government spends less on medical goods, in particular above the median GDP capita, and slightly more on immunisations and early disease detection at the very lowest levels of GDP per capita.

## Discussion

This study identifies health spending trends and country-specific deviations for 184 nations from 1995 to 2014. The findings show that, on average, economic development is associated with increases in health spending per person and decreases in the share of spending that is financed OOP. In addition to this, we show that development assistance for health also fits neatly into the framework outlined in the health financing transition—like OOP health spending, the share of health financing from development partners decreases precipitously with economic development.

Figure 3 highlights three distinct health financing stages that emerge along the spectrum of economic development. In the first stage, health financing is dominated by development assistance for health and OOP. In the second stage, development assistance for health subsides, and the primary sources of health-care financing are OOP and domestic government spending. Finally, the third stage includes countries with the highest GDP per capita, which tend to finance health care using government spending. While these stages are conceptually clear, and are neatly shown in figure 3, it is important to note the great deal of variation that exists around these trends. While the empirical evidence supporting the health financing transition is robust on average, many counter-examples exists. Some upper-middle-income countries such as Angola and Turkmenistan spent only $228 and $396, respectively, per capita on health in 2014. Similarly, some lower-middle-income countries such as Sudan and Federated States of Micronesia rely heavily on OOP and development assistance for health. Despite the clear and intuitive trends articulated by the health financing transition, an important conclusion of this research is that country-specific characteristics are central to health financing, and that time and economic development do not guarantee a transition towards adequate prepaid spending needed to support universal health coverage. The trends shown here are encouraging, although substantive deviation from the trends are in some cases worrisome.

At the nexus of economic development and health financing are concerns related to what some have called the missing middle. This missing middle concept, which has been debated, asserts that in the process of transitioning from low-income to middle-income status, countries receive less development assistance for health although they are not yet able to domestically raise sufficient government resources to replace the lost external resources.^43^, ^44^ Identifying, understanding, and managing this potential is of crucial importance because more than 70% of the world's poor live in middle-income-countries.^45^ A result of a premature transition from development assistance for health would be a reduction in prepaid, pooled resources, and an increase in OOP financing. Our estimates provide some evidence of this occurrence, because lower-middle-income countries finance more of their health spending OOP than any other income group.

Trends in development assistance for health disbursement draw even more attention to the missing middle concern, because the growth rates of development assistance for health disbursement have slowed substantially since 2010, causing some donors to re-evaluate their own development assistance for health allocation policies.^46^ Along with this, some key health focus areas have recently experienced tepid growth, and funds for HIV/AIDS, which for the past 15 years has been the largest development assistance health focus area, have contracted since 2010. In some cases this comes after major gains have been made.^47^, ^48^, ^49^ For many low-income countries and some missing middle-income countries, these trends could have crucial impacts on the provision of essential health services, unless the trends reverse or domestic funds are identified.

Development assistance for health disbursements grew substantially between 2000 and 2010. Although this period, referred to as the so-called golden age of development assistance for health growth, saw annual disbursement more than triple, how these disbursements affected total health spending in low-income and middle-income countries remains unclear.^50^ In some cases, this disbursement might prop up domestic spending and hide the true amount of spending that is being financed domestically. In other cases, development assistance could have crowded out or replaced government spending that would have otherwise exsisted.^51^, ^52^ Because development assistance for health is not always predicable and sustainable, ongoing maturation of domestic prepaid financing is important.

While a shift from a reliance on development assistance for health can be externally imposed upon some countries, the shift from a reliance on OOP must be driven by internal, institutional forces. Political efforts and health-system reform have encouraged movement away from OOP financing in countries such as Mexico and Thailand. While these countries reduced the share of health spending that was OOP as middle-income countries, this transition occurs, on average, later in the process of economic development. Our estimates (Figure 2, Figure 3) show that while the share of health spending from OOP peaks at GDP per capita of $2456, it remains a major source of spending for many countries beyond this point. The modelled trends do not show an accelerated decline in spending from OOP till above GDP per capita of $20 000. This threshold is crucial because OOP financing has been linked to less access to prescribed medicines, less access to care, more adverse health outcomes, and impoverishment.^15^, ^16^, ^20^, ^53^

In addition to tracking total health spending and health spending by source, this study also highlights how the type of health-care services purchased is relatively stable across the spectrum of economic development. While these trends are quite stable in relative terms, the exponential growth of total health spending means that in absolute terms, much more is being spent on each type of goods and service in high-income countries than in low-income countries. In relative terms, trends do show that countries with the lower GDP per capita spent more proportionally on medical goods, which includes pharmaceuticals, and their governments spend relatively more on immunisation and early disease detection. Most clear is that spending on long-term care is minimal in low-income countries, whereas it grows to 20% in high-income countries. In many low-income countries, long-term care remains informal and relies on volunteers and community care.^54^

Long-term care is only one type of service associated with the epidemiological transition and the shift from a health burden dominated by infectious and childhood diseases to a health burden dominated non-communicable, chronic conditions. While many middle-income countries are dealing with this double burden simultaneously, planning within low-income countries should revolve around ensuring progress along this epidemiological transition, while also anticipating the additional financing challenges associated with this transition.^55^ In many cases, these challenges include caring for an older population with chronic conditions and in many cases complex comorbidities. Recent US research shows that in the USA, 70% of personal health spending is on non-communicable diseases.^56^ This research shows that a dramatic shift in how resources are allocated across types of goods and services is not the norm, although, in most cases, considerably more resources are used to care for these populations.

Sufficient supply of prepaid, pooled health resources, such as government spending, is crucial in the pursuit of universal health coverage.^57^ If, like the health financing transition describes, this health spending is encouraged by economic development, then economic growth is a catalyst for progress towards universal health coverage. This research affirms these trends on average, but also highlights substantial deviations from these trends. The reality is that many factors determine a country's health spending level, including factors related to the population's age pattern and health burden, the supply and demand of health care, prices, adaptation of new medical technology, and many health-system characteristics, including health-system efficiency.^58^, ^59^, ^60^ These same factors might also be integral in transitioning from reliance on OOP financing to prepaid financing. While national income can constrain health spending, these shifts in financing might be more connected to comprehensive socioeconomic development. Moreover, health-system reform is generally a political process that is affected, but not purely determined, by any one of these additional factors. Ongoing assessment of these factors is crucial for informed pursuit of universal health coverage.

This research has several limitations that revolve around the data availability and data quality. The WHO Global Health Observatory is the only curator of some of the estimates used in this study: government, prepaid private, and OOP health spending estimates that span worldwide. These data are intended to be comparable, although estimates across time and release versions can vary substantially. The estimates of private spending, including OOP and prepaid private spending, are particularly concerning because the underlying input data are sparse and vary in quality. In addition, these data are reported as spending agent, rather than as the source of the financing. Spending estimates fluctuate drastically across time for many countries and might hint at inconsistency in tracking across time rather than dramatic spending shifts. Moving forward, more effort is needed to evaluate and validate these health financing data.

An additional data limitation is that NHA reports are sparse and contain gaps.^61^ These issues prevent analysis at a more granular level. Although the framework for completing NHAs is standardised, the actual methods used to complete an NHA report vary across countries and time. This is particularly the case in many low-income and middle-income settings, where generation of an NHA relies less on routinely collected data and more heavily on approximation. Like the data from the WHO Global Health Observatory, these data are tremendously powerful, although scarcity and quality suggest additional assessment, validation, and correcting of any discovered biases is necessary.

Furthermore, like the spending by source data from the WHO and the spending by type data from the NHAs, IHME's development assistance for health data relies on estimation and approximation to fill in incomplete data. In particular, estimates of development assistance for health rely on modelling of disbursement data when only commitment data are available and keyword searches of project level data when health focus areas are not clearly identified. In addition, these data capture development assistance for health from the bilateral aid agencies from high-income countries only, missing any so-called South–South cooperation in the health sector. In addition to these, some development assistance for health is not allocable to a specific country because the development agency does not report project level information. When development assistance for health can be allocated to a specific country, it might remain unclear if all the development assistance for health was actually disbursed within that country. Because country-specific development assistance for health estimates are used to estimate domestic government and private health spending, development assistance for health measurement error might cause measurement error in these other metrics. Measurement error and misalignment of government spending and development assistance for health even lead to domestic government spending estimates for some countries falling below zero, after development assistance for health provided to the government is removed. More transparency and complete reporting of development assistance for health, including precise information about where resources are spent and who was the primary recipient, would lead to improved development assistance for health tracking.

Two final data limitations are that many of the estimates reported in this study are reported using PPP $ and none of the data sources used for this study provide subnational spending estimates. The empirical basis for some purchasing power parity adjustments is weak, despite ongoing efforts to improve them.^62^ In addition, the absence of subnational data prevented the examination of within-country heterogeneity in health financing. GDP per capita does not capture income equality, and health spending within many countries varies substantially, and many cases substantial health spending inequalities exist.^63^, ^64^ Spending levels and the share prepaid can vary greatly, particularly in countries with a small government share of health spending. Many key dimensions, such as income, wealth, race, geography, age, sex, and economic status, are glossed over when estimates are reported in national, per capita terms only. Because this study focuses on national spending, it abstracts from these critical variations. Future analyses should aim to assess the health financing transition within countries, while globally more efforts need to be made to collect subnational health financing data.

In addition to these data concerns, it is also important to recognise that this research has not tracked health spending in relation to health outcomes. More spending on health does not guarantee better health outcomes. A recent report from the OECD have shown that a substantial share of health spending in high-income countries is wasted.^65^ Coupled with efforts to ensure adequate prepaid resources for health must be efforts to ensure that health services are provided efficiently and equitably.

In conclusion, the availability of prepaid resources for health, such as government spending, is one of many determinants of access to health care, and can lead to population health gains. Economic development is associated with an increase in spending and specifically an increase in prepaid resources. This is at the core of the pursuit for universal health coverage. This research also points to countries that deviate from the trends, spending more or less than expected, based on their level of economic development. This information is valuable to planners assessing funding gaps and financing opportunities, and can be used to provide insight into what future health financing challenges are likely. Tracking changes in health financing patterns across time and benchmarking against global trends is vital to addressing missed opportunities, ensuring access to medicines and high quality services, and the pursuit of universal health coverage.

Correspondence to: Dr Joseph L Dieleman, 2301 Fifth Avenue, Suite 600, Seattle, WA 98121, USA **dieleman@uw.edu**

For **WHO global health expenditure database** see http://apps.who.int/nha/database

For **WHO Global Health Observatory** see http://www.who.int/gho/en/

For the **International Monetary Fund world economic outlook database** see https://www.imf.org/external/pubs/ft/weo/2016/02/weodata/index.aspx

For the **World Bank Databank** see http://databank.worldbank.org/data/home.aspx

**This online publication has been corrected. The corrected version first appeared at thelancet.com on May 18, 2017**
