# Supplementary material for: Evolution and patterns of global health financing 1995–2014: development assistance for health, and government, prepaid private, and out-of-pocket health spending in 184 countries
Source: Lancet. 2017 May 20;389(10083):1981–2004. doi: 10.1016/S0140-6736(17)30874-7 (PMC5440770; doi:10.1016/S0140-6736(17)30874-7)
Supplement: Supplementary appendix [file mmc1.pdf]

THE LANCET

Supplementary appendix

This appendix formed part of the original submission and has been peer reviewed. We post it as supplied by the authors.

Supplement to: Global Burden of Disease Health Financing Collaborator Network. Evolution and patterns of global health financing 1995–2014: development assistance for health, and government, prepaid private, and out-of-pocket health spending in 184 countries. *Lancet* 2017; published online April 19. http://dx.doi.org/10.1016/S0140-6736(17)30874-7.

SUPPLEMENTARY METHODS ANNEX

State of Global Health Financing

Version: April 6, 2017

Global Burden of Disease Health Financing Collaborator Network – full author list in final pages of appendix

Table of Contents

SECTION 1. INTRODUCTION 3

SECTION 2. DATA 4

Data Sources and Databases 4

Institute for Health Metrics and Evaluation’s Development Assistance for Health Database 4

World Health Organization's Global Health Observatory 19

IMF, UN, PWT, and World Bank Data 19

National Health Accounts 20

Institute for Health Metrics and Evaluation’s GDP series 25

Descriptive Statistics 25

SECTION 3. STATISTICAL MODEL 27

By Source 27

By Type 28

Robustness Analyses 29

SECTION 4. SUPPLEMENTARY TABLES & FIGURES 41

REFERENCES 69

SECTION 1. INTRODUCTION

The objective of this study is to describe the state of global health finance. In order to achieve this objective, we conducted three types of analyses. First, we tested the two main propositions of the health financing transition which characterize the trends in health financing as countries achieve social and economic development: (i) increases in health spending per capita and (ii) decreases in the share of health spending that is out-of-pocket. Second, we extend the literature on the characterization of trends associated with the health financing transition by examining how expenditure on different types of care changes as countries develop. Third, we identify countries whose paths are different from what is posited by the health financing transition based on their level of development.

This type of analyses is both timely and relevant in light of the global push for universal health coverage (UHC) for citizens around the world. According to the propositions of the health financing transition, as countries become more developed, the proportion of total health expenditure that is government-financed increases. This trend suggests that development will be supportive of the attainment of UHC.

The purpose of this appendix is to describe in detail the methodology used in our analyses. Subsequent sections contain information on all data sources, model estimation parameters, and assumptions utilized in this analysis. Section 2 describes how the datasets used for the analyses were created. Section 3 details how the data were used in our analyses.

SECTION 2. DATA

We used data from eight sources for the analyses: (i) WHO's Global Health Observatory (WHO); ii) Institute for Health Metrics and Evaluation's Development Assistance for Health Database (IHME); iii) National Health Accounts (NHA); iv) International Monetary Fund's World Economic Outlook Database (IMF); v) World Bank's World Development Indicators Database (WB); vi) United Nations' National Accounts Main Aggregates Database (UN); vii) Penn World Tables (PWT); and viii) IHME GDP series. Specifically, data on health spending by source that are comparable across countries and complete for most countries were extracted from the WHO Global Health Expenditure Database and IHME's 2016 Development Assistance for Health Database, while data on health spending by type was extracted from National Health Accounts. The UN, PWT, IMF, and World Bank databases provided exchange rate and deflator series. Table 1 below presents the definitions for the various health expenditure sources.

Data sources and databases

Table 1. Definitions of health expenditure sources

Health expenditure type	Definition
**Development assistance for health**	Financial and in-kind contributions from global health channels that aim to improve or maintain health in low- or middle-income countries.
**Government health expenditure as source**	Government health expenditure as source only includes domestically financed government expenditure on health.
**Out-of-pocket expenditure**	Payment by individuals for health services, considered catastrophic if exceeding 40% of a household's annual income.
**Prepaid private health expenditure**	Private risk pooling against catastrophic health expenditure; includes private insurance and non-governmental organizations.

Institute for Health Metrics and Evaluation's Development Assistance for Health Database

Development assistance for health (DAH) estimates were obtained from the Institute for Health Metrics and Evaluation's Development Assistance for Health Database. We summarize the original methodology as well as updates for this year's estimates below. A more detailed description of the original methodology used to obtain the estimates in the database can be found in Dieleman et al.[1] All known, systematically reported, available data on health-related disbursements and expenditures were extracted, as well as income and revenue from existing project databases, annual reports, and audited financial statements. The channels included and the corresponding data sources are summarized in Table 2. Data sources obtained via personal correspondence are summarized in Table 3.

DAH for bilateral agencies included all health-related disbursements from bilateral donor agencies, excluding funds that they transferred to any of the other channels we tracked in order to avoid double-counting. This information was extracted from the Creditor Reporting System (CRS) and Development Assistance Committee (DAC) databases of the Development Assistance Committee of the Organisation for Economic Co-operation and Development (OECD-DAC). In some cases, donor agencies did not report disbursement data to the CRS. A method for predicting disbursements from commitment data was implemented to address this challenge. For detailed description of this method see Dieleman et al.[1]

For other grant- and loan-making institutions, annual disbursements on health grants and loans were similarly included, excluding transfers to any other channels and ignoring any repayments on outstanding debts. For a more detailed description of this process see Dieleman et al.[1] The annual disbursements for grant- and loan-making institutions only reflect the financial transfers made by these agencies. Therefore, in-kind transfers from these

institutions in the form of staff time for providing technical assistance and the costs of managing programs were estimated separately.[1]

Estimates of DAH for the United Nations (UN) agencies included annual expenditures on health both from their core budgets and from voluntary contributions. Calculating DAH for the United Nations Children's Fund (UNICEF) involved estimating the fraction of its total expenditure spent on health prior to 2001.[1]

Non-governmental organizations' (NGOs) DAH estimates utilized data from US government sources and a survey of health expenditure for a sample of NGOs to estimate DAH from US-based and internationally based NGOs receiving support from the US government. We were unable to include other NGOs due to the lack of audited and comparable data.

The database also included an analysis of the composition of health funding by recipient country, as well as by health focus area. Although our methods for this year's estimates did not change significantly, we made two key changes – improvement to preliminary estimation for some bilateral donors and improvements to our health focus area keyword search terms – that are detailed in Table 3 and in the section below titled "Disaggregation by health focus area," respectively.

For many channels, reporting-time lags prevent primary disbursement data for the most recent year(s) from being available. For those years, the values of DAH were predicted, using channel-specific time trends. The methods employed to obtain these predictions are summarized in Table 4. In general, these methods depend on data availability. The estimates are based on channel-specific budget, commitment, and appropriations data, and in many cases assume the most recent disbursement patterns persist. Due to the lack of more detailed disaggregated data, estimates are not provided by recipient.

All results are presented in real 2015 US dollars. All disbursement sequences were converted into real 2015 US dollars by taking disbursements in nominal US dollars in the year of disbursement and adjusting these sequences into real 2015 US dollars using US gross domestic product (GDP) deflators. Analyses were conducted in the following programs: Stata (version 13.1), Amelia (version 1.7), and R (version 3.3.2).

Table 2. Summary of primary data sources and databases

Channel	Source
**Bilateral agencies**	OECD-DAC and CRS databases[2]
**European Commission**	OECD-DAC and CRS databases[3]
**Joint United Nations Programme on HIV/AIDS (UNAIDS)**	Financial reports and audited financial statements[4]
**United Nations Children's Fund (UNICEF)**	Financial reports and audited financial statements[5–7]
**United Nations Population Fund (UNFPA)**	Financial reports and audited financial statements[8]
**Pan American Health Organization (PAHO)**	Financial reports and audited financial statements[9]
**World Health Organization (WHO)**	Financial reports and audited financial statements[10]
**World Bank**	Online project database and correspondence[11,12]
**Asian Development Bank (ADB)**	Online project database[13]
**African Development Bank (AfDB)**	Online project database and compendium of statistics[14,15]
**Inter-American Development Bank (IDB)**	Online project database and correspondence[16,17]
**Gavi, the Vaccine Alliance**	Online project database, cash received database, International Finance Facility for Immunisation (IFFIm) annual reports, Advance Market Commitment for Pneumococcal Vaccines (AMC) annual reports, and annual reports[18–21]
**The Global Fund to Fight AIDS, Tuberculosis and Malaria (Global Fund)**	Online grant database, contributions report and annual reports[22–24]
**NGOs registered in the US**	United States Agency for International Development (USAID) Report of Voluntary Agencies (VolAg), tax

	filings, annual reports, financial statements, RED BOOK Expanded Database, and WHO's Model List of Essential Medicines[25–28]
**Bill & Melinda Gates Foundation (BMGF)**	Online grant database, IRS 990 tax forms, and correspondence[29,30]
**Other private US foundations**	Foundation Center's grants database[31]

Table 3. Data sources received via personal correspondence

Channel	Data received
**World Bank**	Health project-level disbursement data, 1990–September 2016[12]
**BMGF**	Health disbursement data, 2015[30]
**IDB**	Health project-level loan disbursement data, 2016[17]

Table 4. Additional data sources, databases and model choices used for preliminary estimates of DAH

Channel	Data source	Variables used	Years of budget data used for modeling*	Years underlying DAH data not available; thus modeled*	Model used
**National agencies**					
**Australia**	Australia's International Development Assistance (2008–2016); Australia's Overseas Aid Program (1998–2008)[32,33]	Health official development assistance (ODA): International development assistance budget	1998–2016	2015–2016	Weighted average of actual DAH/budgeted DAH
**Austria**	Austria Federal Ministry of Finance budget[34]	General ODA: Federal ODA budget	2007–2016	2015–2016	Weighted average of DAH/budgeted ODA
**Belgium**	Project Budget General – general expenses[35]	General ODA: Foreign affairs, foreign trade development and cooperation	2000–2016	2015–2016	Weighted average of DAH/budgeted ODA
**Canada**	Canadian International Development Agency – Report on Plans and Priorities[36]	General ODA: Financial summary – planned spending	1996–2016	2015–2016	Weighted average of DAH/budgeted ODA
**Denmark**	Danish Ministry of Foreign Affairs Budget[37]	General ODA: Budgeted expenditures on overseas development assistance	2000–2016	2015–2016	Weighted average of DAH/budgeted ODA
**European Commission**	General budget[38]	Data not used as they were inconsistent with disbursements	–	2015–2016	Based on weighted average of trends in member countries
**Finland**	Document Assembly in budget years 1998–2016[39]	General ODA: Ministry of Foreign Affairs' administrative appropriations, international development	2002–2016	2015–2016	Weighted average of DAH/budgeted ODA
**France**	Budget and Financial documents[40,41]	General ODA: aggregated project data; Total ODA	2009–2016	2015–2016	Weighted average of DAH/budgeted ODA
**Germany**	Plan of the Federal Budget[42]	General ODA: Development expenditure	2001–2016	2015–2016	Weighted average of DAH/budgeted ODA

Channel	Data source	Variables used	Years of budget data used for modeling*	Years underlying DAH data not available; thus modeled*	Model used
**Greece**	Ministry of Finance Budget (2013–2016); OECD Data (1996–2012)[2,43]	General ODA; ODA commitments	1996–2013	2015–2016	Weighted average of DAH/budgeted ODA
**Ireland**	Department of Finance – budget 2000–2004; Estimates for Public Services and Summary Public Capital Programme, 2005–2016[44]	General ODA: Summary of adjustments to gross current estimates – international co-operation	2002–2016	2015–2016	Weighted average of DAH/budgeted ODA
**Italy**	The Italian Agency for Development Cooperation[45]	General ODA: Net development corporation	2007–2016	2015–2016	Weighted average of DAH/budgeted ODA
**Japan**	Highlights of the Budget for FY1999–2016[46]	General ODA: Major budget expenditures	2003–2016	2015–2016	Weighted average of DAH/budgeted ODA
**Korea, South**	ODA Korea comprehensive implementation plan[47]	General ODA: Plan for international development cooperation	2008–2016	2015–2016	Weighted average of DAH/budgeted ODA
**Luxembourg**	State Budget[48]	General ODA: Ministry of Foreign Affairs – budgeted international development cooperation and humanitarian aid	2001–2016	2015–2016	Weighted average of DAH/budgeted ODA
**Netherlands**	Netherlands International Cooperation Budget (2001–2016)	General ODA: Total annual official development assistance expenditure	2001–2016	2015–2016	Weighted average of DAH/budgeted ODA
**New Zealand**	Vote Foreign Affairs and Trade (1998–2001); VOTE Official Development Assistance (2002–2016)[49]	General ODA: Total annual official development assistance expenditure	1998–2016	2015–2016	Weighted average of DAH/budgeted ODA

Channel	Data source	Variables used	Years of budget data used for modeling*	Years underlying DAH data not available; thus modeled*	Model used
**Norway**	Norwegian Ministry of Finance National Budget (2014–2016); Correspondence (2000–2013)[50,51]	General ODA: ODA budget	2000–2016	2015–2016	Weighted average of DAH/budgeted ODA
**Portugal**	Ministry of Finance and Public Administration State Budget 2003–2016[52]	General ODA: Integrated service expenditure – external cooperation budget	2003–2016	2015–2016	Weighted average of DAH/budgeted ODA
**Spain**	Annual Plans of Spanish International Cooperation[53]	General ODA: Spanish total development cooperation	2003–2016	2015–2016	Weighted average of DAH/budgeted ODA
**Sweden**	Correspondence (2000–2010); Ministry of Foreign Affairs Budget (2010–2016)[54]	General ODA: Ministry for Foreign Affairs budgets for expenditure – international development cooperation	2000–2016	2015–2016	Weighted average of DAH/budgeted ODA
**Switzerland**	Foreign Affairs (2000–2006); Budget – Further Explanations and Statistics (2007–2016)	General ODA: Direction of development and cooperation (2000–2006); foreign affairs – international cooperation, development aid (in the South and East) (2007–2016)	2000–2016	2015–2016	Weighted average of DAH/budgeted ODA
**United Kingdom**	IATA (Department for International Development (DFID))[55,56]	General ODA: assistance for international development; Sum (revised) – aggregated project data	1998–2016	2015–2016	Weighted average of DAH/budgeted ODA
**United States**	Foreign Assistance Dashboard (2006–2016); Budget of the US Government (2005–2016)[57,58]	Global health ODA: Planned foreign assistance for health; Department of Health and Human Services global health budget	2005–2016	2015–2016	Weighted average of actual DAH/budgeted DAH
**UN agencies**					
**WHO**	Programme budget[59]	DAH budget: Programme budget	2002–2016	2015–2016	Weighted average of DAH/budget

Channel	Data source	Variables used	Years of budget data used for modeling*	Years underlying DAH data not available; thus modeled*	Model used
**UNAIDS**	Unified Budget and Workplan, bienniums 2002–2016[60,61]	DAH budget: Unified Budget and Workplan	2002–2016	2015–2016	Weighted average of DAH/Core Budget
**UNICEF**	Financial report and audited financial statements; correspondence[7,62,63]	Total expenditure; Total health expenditure	2001–2015	2015–2016	Weighted average of DAH/budget
**UNFPA**	Audited Financial report and contributions report [64,65]	Total health expenditure	2002–2015	2015–2016	Weighted average of DAH/budget
**PAHO**	Proposed program budget[9]	Total regular budget, estimated voluntary contributions	2000–2016	2015–2016	Weighted average of DAH/budget
**NGOs**	VolAg (1990–2011), GuideStar (2014), sample of top NGOs (2011–2012) [25,26]	Revenue breakdowns for: US public, non-US public, private, in-kind, BMGF; total overseas expenditures	1990–2013	2014–2016	Regression on DAH, US GDP, and USAID and private voluntary organization (PVO) revenue

* Years of budget data used for modeling versus years underlying DAH data unavailable thus modeled: The data used to estimate DAH by channel vary across channels. Table 2 reports our primary data used for each channel. Due to reporting lags there are some years we need to estimate disbursement using additional data sources. These additional data sources, the years in which the primary data are modeled, the years the additional data are available, and the methods for this estimating these modeled years are reported in Table 4. Years of budget data used for modeling are the years of additional data available to us. We rely on historic trends to inform our estimates so we rely on many years of additional data despite only modeling a few years of primary data. Years underlying DAH data unavailable thus modeled are the years the primary data are incomplete and thus estimated using additional data. See example below for more details for Australia.

Box 1. EXAMPLE – Australia's primary and additional data sources

Project-level data for health-related projects funded by Australia's bilateral aid agencies are available from the OECD's CRS database through 2014. This is the primary data source used to estimate DAH channeled by Australian aid agencies, as described in Table 2. 2015–2016 are incomplete because of lags in reporting. To estimate DAH disbursed for 2015 and 2016, additional data are available from Australia's International Development Assistance budget (2008–2016) and Australia's Overseas Aid Program budget (1998–2008), as described in Table 4. These sources provide health-specific official development assistance (ODA) budgeted by Australia, 1998–2016. To estimate DAH disbursed 2015–2016, we calculated the ratio of disbursed DAH (from the CRS database) relative to budgeted DAH (from the International Development Assistance and Overseas Aid Program budgets) for 1998–2014. We combine the most recent three ratios into a single estimate by taking a weighted average, weighting substantially higher the most recent year. We multiply this ratio – the estimated disbursed DAH to budgeted DAH – by the 2015 and 2016 budgeted DAH to estimate disbursed DAH in those years. These methods are described more fully in Dieleman et al.[1]

DISAGGREGATING BY HEALTH FOCUS AREA

We improved our analysis of the disaggregation of health funding by health focus areas by augmenting our keyword search terms. In particular, we added health system strengthening as a category under each health focus area. Similar to our previous work, the analysis of health focus areas included assessments of development assistance for HIV/AIDS; tuberculosis (TB); malaria; maternal health; newborn and child health; other infectious diseases; non-communicable diseases; and SWAps and health sector support, using keyword searches within descriptive fields. These were chosen as the areas of focus because of their relevance to current policy debates about global health financing and data availability.

In effect, DAH was disaggregated into eight health focus areas: HIV/AIDS; tuberculosis; malaria; maternal, newborn, and child health; non-communicable diseases; SWAps/health sector support; other infectious diseases; and other. For most data sources, project-level data were available only through 2014. Methods to estimate health focus area allocations for 2015 and 2016 are described in more detail below. Keyword searches were performed for a subset of global health channels that provide project-level data with project titles or descriptions. These sources include the bilateral development assistance agencies from 23 DAC member countries, the EC, Global Fund, the World Bank, ADB, AfDB, IDB, BMGF, NGOs, and US foundations. The keywords used are outlined in Table 5 below. Descriptive fields were adjusted so that they were in all capitalized letters, and search terms with multiple words were put between quotation marks. All keywords were translated into nine major languages (English, Spanish, French, Portuguese, Italian, Dutch, German, Norwegian, and Swedish) used in the OECD CRS, checked for double meanings across all languages, and adjusted accordingly.

Total DAH was split across the health focus areas using weighted averages based on the number of keywords present in each project's descriptive variables. If, for example, three keywords suggested the project focused on HIV/AIDS and two keywords related to tuberculosis were also tagged, three-fifths of the project's total DAH was allocated to HIV/AIDS and two-fifths was allocated to tuberculosis. To account for the sensitivity of this method, several checks were implemented after the keyword searches to ensure the project was accurately categorized. First, projects that were tagged as child and newborn vaccines and other infectious diseases were categorized as child and newborn vaccines only. Second, projects that were tagged as one of the three major infectious diseases (HIV/AIDS, tuberculosis, or malaria) and other infectious diseases were categorized under only HIV/AIDS, tuberculosis, or malaria.

Box 2. EXAMPLE. Post-keyword search weighting

A project in the CRS database had a value of $1,000 of DAH. A keyword search conducted on this project's title and description tagged 5 keywords: 3 keywords related to HIV/AIDSs and 2 keywords related to tuberculosis. Therefore, $600, or 3/5 of total DAH, was allocated to HIV/AIDS, while $400, or 2/5 of total DAH, was allocated to tuberculosis.

In addition to keyword searches, funds were allocated to health focus areas based on characteristics of the channel or additional channel variables. For the bilateral agencies and the EC, purpose codes from the CRS were used to supplement keyword searches. For the World Bank-IDA and -IBRD, health focus areas were also determined by the project sector codes and theme codes, which included percentages of health funds that targeted each theme. All funds from Gavi were allocated to child and newborn vaccines, health sector support, and non-communicable diseases, and all funds from UNICEF to maternal, newborn, and child health, unspecified. Funds from the Global Fund were distributed to malaria, HIV, TB, and health sector support based on disease components. Within each disease component, keyword searches on programmatic budget data and project descriptions were conducted to distribute among program areas. Funds from UNAIDS were allocated to HIV/AIDS, and specific program areas were determined by budget information. UNFPA and WHO funds were allocated to specific health focus areas based on project expenditure data from their annual reports and annual financial reports. For all channels, projects listed as HIV/TB were distributed evenly between the two health focus categories. See Table 6 below for more details on these categorizations.

Table 5. Terms for keyword searches

Health focus area level I	Health focus area level II	Keywords
**HIV/AIDS**	HIV envelope/unidentified	" HUMANIMMUNODEFVIRUS " " SIDA " " OVC " " H I V " " HIV " " AIDS " " HUMAN IMMUNODEFICIENCY " " REVERSE TRANSCRIPTASE INHIBITOR " " ACQUIRED IMMUNE DEFICIENCY SYNDROME " " ACQUIRED IMMUNNODEFICIENCY " " RETROVIRAL " " VCT " " MALE CIRCUMCISION " " ART " " ARV " " CD4 COUNT " " HAART " " PMTCT " " MOTHER TO CHILD TRANSMISSION" " MOTHER TO CHILD AIDS TRANSMISSION" " PARENT TO CHILD TRANSMISSION" " PRESIDENT'S EMERGENCY PLAN FOR AIDS RELIEF " " PEPFAR " " THREE DISEASE FUND " " 3 DISEASE FUND "
	Care and support	" CAREANDSUPPORT " " CARE ACTIVIT" " PAIN RELIEF " " SYMPTOM RELIEF " " SOCIAL SUPPORT " " CHRONICALLY ILL " " CLINICAL MONITORING " " CARE AND SUPPORT " " PSYCHOLOGICAL SERVICE" " PSYCHOLOGICAL SUPPORT " " PSYCHOSOCIAL SUPPORT " " PSYCHOSOCIAL SERVICE" " MATERIAL SUPPORT "
	Counseling and testing	" COUNSELING " " TESTING " " VCT " " COUNSELLING "
	Orphans and vulnerable children	" VULNUERABLECHILD" " OVC " " ORPHAN" " VULNERABLE CHILD" " INFECTED CHILD" " VULNERABLE GROUP" " MOST AT RISK "
	Prevention of mother-to-child transmission (PMTCT)	" MOTHERTOCHILD" " MOTHER TO CHILD" " PARENT TO CHILD" " PMTCT "
	Prevention	" CONDOM" " PREVENT" " HIV EDUCATION " " AIDS EDUCATION " " REDUCING THE TRANSMISSION OF HIV " " REDUCE THE TRANSMISSION OF HIV " " MALE CIRCUMCISION" " SAFE BLOOD SUPPL" " SAFE INJECTION" " ABSTINENCE " " AWARENESS " " BLOOD SAFETY " " MICROBICIDE"
	Treatment	" RETROVIRAL " " TREAT" " ART " " ARV " " CD4 COUNT " " HAART " " VIRAL LOAD " " VIRAL BURDEN " " VIRAL TITER " " ESSENTIAL SERVICE" " DRUG REGIMENS " " IMPACT REDUCTION " " REDUCE IMPACT "
Tuberculosis		" TUBERCULOS" " TB " " TUBERCULAR" " DOTS " " DIRECTLY OBSERVED TREATMENT " " RIFAMPICIN " " ISONIAZID " " MULTI DRUG RESISTANT " " THREE DISEASE FUND " " 3 DISEASE FUND "
**Malaria**	Malaria envelope/unidentified	" MALARIA " " PLASMODIUM FALCIPARUM " " ANOPHELES " " ARTEMISININ " " PRIMAQUINE " " INDOOR RESIDUAL SPRAY" " INDOORRESIDUALSPRAY" " IRS " " PLASMODIUM VIVAX " " BEDNETS " " BED NETS " " SMITN " " ITN " " LLIN " " INSECTICIDAL NET" " INSECTICIDE TREATED NET" " THREE DISEASES FUND " " 3 DISEASES FUND "
	Diagnosis	" DIAGNOSIS " " DIAGNOSTIC " " CASE DETECTION " " MICROSCOPY " " BLOOD SURVEY " " RAPID DIAGNOSTIC TESTING " " MOBILE MALARIA CLINIC " " BIOLOGICAL TESTING " " LABORATORY SERVICES " " EDT " " LAMP " " RDT "

	Community outreach	" COMMUNITYOUTREACH " " COMMUNITY OUTREACH " " COMMUNITY MOBILIZATION" " AWARE" " COMMUNICATION STRATEGY " " SOCIAL COMMUNICATION " " HEALTH EDUCATION " " PARTNERSHIP" " PUBLIC SECTOR" " ACTIVITIES NEAR COMMUNITIES "
	Vector control: bed nets	" BEDNET" " BED NET" " SMITN " " ITN " " LLIN " " INSECTICIDAL NETS " " INSECTICIDE TREATED NET" " INSECTICIDE TREAT"
	Vector control: IRS	" INDOORRESIDUALSPRAY" " IRS " " REDUCE THE PARASITE RESERVOIR " " FOGGING " " COILS " " LARVICID" " LARVACID" " VECTOR CONTROL" "RESIDUAL SPRAY " " RESIDUALSSPRAY " "INDOOR SPRAY" " INDOORSPRAY "
	Vector control: other than bed nets and IRS	" PREVENT"
	Treatment	" ARTEMISININ " " PRIMAQUINE " " ACT " " DRUG ADMINISTRATION " " TREAT " " TREATMENT " " CASE MANAGEMENT " " COMBINATION THERAPY " " ANTI MALARIAL " " ANTIMALARIAL "
Maternal, newborn, and child health	envelope/unidentified	" FERTILITY " " FAMILY PLANNING " " FP " " BIRTH" " WOMEN HEALTH " " WOMEN S HEALTH " " WOMENS HEALTH " " CONTRACEP" " IPPF " " INTERNATIONAL PLANNED PARENTHOOD FOUNDATION " " ABORTION" " UNFPA " " POSTPARTUM " " POST PARTUM " " MATERNAL " " MATERNITY " " MOTHERHOOD " " SBA " " ANTENATAL " " PRENATAL " " NEONATAL " " PERINATAL " " POSTNATAL " " FETUS" " FETAL" " IPTP " " REPRODUCTIVE HEALTH " " OBSTETRIC" " PREGNANCY " " RH " " REPRODUCTIVE HEALTH " " REPROD " " RHCS " " SEXUAL HEALTH " " SYPHILIS " " FISTULA " " SEPSIS " " ANEMI" " ANAEMI" " FOETUS" " FOETAL " " FGM " " FEMALE GENITAL MUTILATION " " FEMALE GENITAL CUTTING " " FEMALE CIRCUMCISION " " SBAS " " NUTRITION " " VITAMIN A " " BREAST FE" " BREASTFE" " FEEDING " " MICRONUTRIENT" " ZINC " " FORTIFICATION " " STUNT" " WASTING " " BABY FRIENDLY HOSPITAL INITIATIVE " " BREASTMILK " " BREAST MILK " " IODINE " " IODIZED " " IODIZATION " " VAD " " LACTAT" " FOLIC ACID " " FOLAT" " VACCINE" " VACCINATION" " IMMUNIZ" " DIPHTHERIA " " TETANUS " " PERTUSSIS " " DTP " " HIB " " ROTAVIRUS " " MEASLES " " IMMUNIS" " HEPB MONO " " HIB MONO " " INJECTION SAFETY " " RUBELLA " " MENINGITIS " " PENTA " " PNEUMO " " TETRA " " GAVI " " CHILDHEALTH " " CHILD HEALTH " " INFANT HEALTH " " NEWBORN HEALTH " " CHILD MORTALITY " " INFANT MORTALITY " " UNDER FIVE MORTALITY " " CHILD SURVIVAL " " INFANT SURVIVAL " " CHILDHOOD ILLNESS" " LRI " " RESPIRATORY INFECTION" " DIARRHEA" " DIARRHOEA" " ORAL REHYDRATION " " ORT " " ORS " " UNICEF " " MNCH" " RNCH " " RCH " " RNH " " MNH " " MCH " " EMAS " " MCNH "
	Maternal health, family planning	" FERTILITY " " FAMILY PLANNING " " FP " " BIRTH SPACING " " CONTRACEPT" " FAMILY SIZE" " IPPF " " INTERNATIONAL PLANNED PARENTHOOD FOUNDATION " " ABORTION" " REDUCED FERTILITY " " UNFPA " " REDUCE FERTILITY " " BIRTH CONTROL "

	Maternal health, unspecified	" POSTPARTUM " " POST PARTUM " " MATERNAL HEALTH " " MATERNAL MORTALITY " " MATERNAL DEATH " " SAFE MOTHERHOOD " " BIRTH ATTENDANT" " SBA " " MATERNAL AND INFANT HEALTH " " ANTENATAL " " PRENATAL " " NEONATAL " " PERINATAL " " POSTNATAL " " FETUS" " FETAL" " IPTP " " REPRODUCTIVE HEALTH " " MATERNITY " " OBSTETRIC" " PREGNANCY " " RH " " REPRODUCTIVE HEALTH " " REPROD " " RHCS " " STD " " STI " " SEXUAL HEALTH " " SEXUALLY TRANSMITTED " " SYPHILIS " " FISTULA " " WOMEN S HEALTH " " WOMENS HEALTH " " SEPSIS " " SEPTICEMIA " " ANEMI" " ANAEMI" " FOETUS" " FOETAL " " FGM " " FEMALE GENITAL MUTILATION " " FEMALE GENITAL CUTTING " " FEMALE CIRCUMCISION " " SBAS "
	Child/newborn nutrition	" NUTRITION " " BIRTH WEIGHT " " BIRTHWEIGHT " " VITAMIN A " " BREAST FE" " BREASTFE" " FEEDING " " MICRONUTRIENT" " ZINC " " FORTIFICATION " " STUNT" " WASTING " " UNDERWEIGHT " " BABY FRIENDLY HOSPITAL INITIATIVE " " BREASTMILK " " BREAST MILK " " IODINE " " IODIZED " " IODIZATION " " VAD " " LACTAT" " FOLIC ACID " " FOLAT" " IRON "
	Child/newborn vaccines	" POLIO " " VACCINE" " VACCINATION" " IMMUNIZ" " DIPHTHERIA " " TETANUS " " PERTUSSIS " " DTP " " HIB " " ROTAVIRUS " " MEASLES " " IMMUNIS" " HEPB MONO " " HIB MONO " " INJECTION SAFETY " " RUBELLA " " MENINGITIS " " PENTA " " PNEUMO " " TETRA " " GAVI "
	Child/newborn other	" CHILDHEALTH " " CHILD HEALTH " " INFANT HEALTH " " NEWBORN HEALTH " " CHILD MORTALITY " " INFANT MORTALITY " " UNDER FIVE MORTALITY " " CHILD SURVIVAL " " INFANT SURVIVAL " " CHILDHOOD ILLNESS" " LRI " " RESPIRATORY INFECTION" " DIARRHEA" " DIARRHOEA" " ORAL REHYDRATION " " ORT " " ORS " " UNICEF "
Non-communicable diseases	Tobacco	" TOBACCO" " SMOK"
	Mental health	" SCHIZOPHRENIA " " MENTAL HEALTH " " NEUROTIC " " NEUROS " " PSYCHOLOG" " PSYCHIATRIC" " EMOTIONAL " " PTSD " " POST TRAUMATIC " " POSTTRAUMATIC " " ALCOHOL " " ADDICTION " " DOWN SYNDROME " " DOWN S SYNDROME " " DOWNS SYNDROME " " BEHAVIORAL " " DEPENDANCE " " DRUG ABUSE " " SUBSTANCE ABUSE " " OPIOID " " COCAINE " " AMPHETAMIN " " CANNABIS " " DEPRESSIVE DISORDER " " DEPRESSION " " DYSTHYMIA " " BIPOLAR " " ANXIETY " " EATING DISORDER " " AUTISM " " ASPERGER " " DEVELOPMENTAL DISORDER " " CONDUCT DISORDER " " INTELLECTUAL DISABILITY " " PHOBIA " " MENTAL DISABILITY " " MENTAL RETARDATION " " DEPENDENCE "

	Non-communicable diseases, unspecified	" CANCER" " CHEMOTHERAPY " " RADIATION " " NEOPLAS " " TUMOR " " DIABET" " INSULIN " " ENDOCRINE " " RHEUMATI" " ISCHAEMIC " " ISCHEMIC " " CIRCULATORY " " CEREBROVASCULAR " " CIRRHOSIS " " DIGESTIVE DISEASE" " OTHER DIGESTIVE " " GENITOURINARY " " UROGENITAL " " MUSCULOSKELETAL " " CONGENITAL " " OBESITY " " OVERWEIGHT " " GLAUCOMA " " HYPERTENSI" " HERNIA " " ARTHRITIS " " CLEFT LIP" " CLEFT PALATE" " PHENYLKETONURIA " " PKU " " SICKLE CELL" " DREPANOCYTOSIS " " HEMOPHILIA " " HAEMOPHILIA " " THALASSEMIA " " GENETIC" " HEART DISEASE" " CARDIOVASCULAR " " CHRONIC RESPIRATORY " " NONCOMMUNICABLE " " NON COMMUNICABLE " " COPD " " STROKE " " CATARACT " " CHRONIC OBSTRUCTIVE PULMONARY DISEASE" " ASTHMA " " SKIN DISEASE" " PHYSICAL DISABILITY " " DENTAL " " ORAL HEALTH " " CVD " " IHD " " CKD " " KIDNEY DISEASE" " MSK "
**SWAps/Health sector support**		" SWAP" " TRAINING " " CAPACITY " " DATA SYSTEM" " SECTOR WIDE APPROACH" " HEALTH SYSTEM " " SECTOR WIDE APPROACH" " SECTOR PROGRAM" " BUDGET SUPPORT" " SECTOR SUPPORT " " HSS " " TRACKING PROGRESS " " SKILLED HEALTH WORKERS " " SKILLED STAFF " " ADEQUATE FACILITIES " " ESSENTIAL MEDICINES " " HEALTH INFORMATION SYSTEM" " POLICY DEVELOPMENT" " EARLY WARNING ALERT AND RESPONSE SYSTEM" " MEDICAL EQUIPMENT " " SURGICAL EQUIPMENT " " HEALTH SECTOR PROGRAM" " HEALTH SECTOR SUPPORT " " SECTOR SUPPORT PROGRAM" " HEALTH INFRASTRUCTURE " " HEALTH INSTITUTIONAL STRENGTHENING " " HSPSP " " M&E "" MONITORING" " SURVEILLANCE " " GOVERNANCE " " HUMAN RESOURCE" " HUMAN CAPITAL " " IMPROVED CAPACITIES " " SCALING UP " " REALLOCATE RESOURCES " " STRATEGIES AND PROGRAME" " HIV STRATEGIES " " PROGRAM IN COUNTRY ACTIVITIES " " STRATEGIC INFORMATION " " PROCUREMENT " " EVIDENCE BASED " " CASE REPORTING " " OUTBREAK PREPAREDNESS " " RAPID RESPONSE STRATEGY " " MEDICAL WORKERS " " HEALTH CARE PERSONNEL " " OPERATIONAL RESEARCH " " SUPPORTIVE ENVIRONMENT " " INFORMATION SYSTEM" " INSECT " " WORKFORCE " " INFRASTRUCTUR " " ADMINISTRATIVE "
Other infectious diseases		" INFECTIOUS " " TRICHURIASIS " " YELLOW FEVER " " WHIPWORM " " TRACHOMA " " SCHISTOSOMIASIS " " SNAIL FEVER " " KAYAYAMA FEVER " " RABIES " " ONCHOCERCIASIS " " RIVER BLINDNESS " " ROBLES DISEASE" " LYMPHATIC FILARIASIS " " ELEPHANTIASIS " " LEISHMANIASIS " " LEISHMANIOSIS " " HOOKWORM " " FOOD BORNE TREMATOD " " FOODBORNE TREMATOD " " FOOD BORNE TREMATOD " " ECHINOCOCCOSIS " " HYDATID DISEASE" " HYDATIDOSIS " " DENGUE " " CYSTICERCOSIS " " CHAGAS " " TRYPANOSOMIASIS " " ASCARIASIS " " TROPICAL DISEASE" " AVIAN " " CHOLERA " " DYSENTERY " " PARASITE DISEASE" " INFLUENZA " " PANDEMIC" " EPIDEMIC" " COMMUNICABLE " " AVIAN

	INFLUENZA " " AVIAN FLU " " FAO " " NEGLECTED TROPICAL DISEASE "

Table 6. Additional health focus area categorizations

Channel	Allocation criteria	Health focus area
**Bilaterals and the EC**	CRS purpose code 13030, family planning	Family planning
	CRS purpose code 13020, reproductive health care	Maternal health, non-family planning
	CRS purpose code 12240, basic nutrition	Child and newborn nutrition
	CRS purpose code 12250, infectious disease control and the keywords "child" or "vaccine" present in descriptive variables	Child and newborn vaccines
	CRS purpose code 13040, STD control including HIV/AIDS	HIV/AIDS
	CRS purpose code 12262, malaria control	Malaria, unspecified
	CRS purpose code 12250, infectious disease control and no other keywords present in the descriptive variables	Other infectious diseases
	CRS purpose code 12263, tuberculosis control	Tuberculosis
**World Bank IDA and IBRD**	Theme code population and reproductive health	Maternal, newborn, and child health, unspecified
	Theme code tuberculosis	Tuberculosis
	Theme code child health	Child and newborn health, unspecified
	Theme code HIV/AIDS	HIV/AIDS
	Theme code malaria	Malaria, unspecified
	Theme code injuries and non-communicable diseases	Non-communicable diseases, unspecified
	Theme code nutrition and food security	Child and newborn nutrition
	Theme code other communicable diseases	Other infectious diseases
	Theme code health system performance	SWAps/health system strengthening
	Theme code social analysis and monitoring	SWAps/health system strengthening
**UNFPA**	Family planning, gender equality, population and development	Family planning
	Reproductive health, sexual health, maternal and newborn health, STI prevention	Maternal health, unspecified
	Data analysis, mobilization, program coordination, monitoring and evaluation, advocacy	Family planning and Maternal health, unspecified, according to proportions between the two.
UNICEF	All DAH	Child and newborn health, unspecified
**UNAIDS**	The keyword search was run on budget information for years 2008–2015<br>Program components in budget documents from 1998 to 2007	All health focus area level two categories under HIV/AIDS
Gavi	All DAH	Child and newborn vaccines
**GLOBAL FUND**	Disease components for Malaria, HIV/AIDS, TB, TB/HIV, and Other (health system strengthening)	All health focus area level two categories under Malaria and HIV and health focus area level one categories for TB and SWAps/HSS

	**Keyword search on program service delivery areas**	
WHO	Reproductive, maternal, newborn, child, and adolescent health (divided by 2); Research in human reproduction	Maternal health, unspecified
	Nutrition	Child and newborn nutrition
	Vaccine-preventable diseases	Child and newborn vaccines
	Reproductive, maternal, newborn, child and adolescent health (divided by 2)	Child and newborn health, unspecified
	Aging and health; gender, equity and human rights mainstreaming	Maternal, newborn, and child health, unspecified
	HIV/AIDS	HIV/AIDS
	Malaria	Malaria
	Tuberculosis	Tuberculosis
	Mental health and substance abuse	Non-communicable diseases, mental health
	Disabilities and rehabilitation; Non-communicable diseases; Violence and injuries	Non-communicable diseases, unspecified
	Neglected tropical diseases; Tropical disease research; Epidemic- and pandemic-prone diseases	Other infectious diseases
	Health system information and evidence; Integrated people-centered health services; National health policies, strategies and plans; Access to medicines and health technologies and strengthening regulatory capacity; Alert and response capacities	SWAps/health system strengthening

Disaggregating preliminary estimates by health focus area

Estimates by health focus area for years in which descriptive data were not available (usually 2016 and in many cases 2015 as well) were obtained by modeling channel-specific DAH per health focus area as a function of time. Out-of-sample validation was used to test the predictive accuracy of a large suite of models, estimating the models using 1990–2010 data and predicting 2011 and 2012. The potential models included fractional multinomial logit regression, OLS regression, autoregressive integrated moving average (ARIMA) models, Epanechnikov kernel-weighted local polynomial smoothing, and multivariable fractional polynomial models. For each model, time was modeled linearly, with splines, and by including lag-dependent variables. Other methodologies considered included modeling health-focus-area-specific DAH as a dollar amount and as a fraction of the channel-specific total DAH. Lastly, models that involved transforming the dependent variable in natural log and logit transformed space were considered. In order to accommodate zero values in the logit transformation, the transformation described in Smithson and Verkuilen were applied.[66] Over 40 models and specifications were evaluated in total.

Each of the potential models and specifications described above were estimated using data from 1990 through 2010, and then the estimated model was used to predict DAH by health focus area for 2011 and 2012. Since we have DAH estimates for 2011 and 2012, we compared the modeled estimates and the observed estimates and calculated average percent deviation and average total absolute deviation for each model and specification across all the channels and health focus areas. A variant of the Epanechnikov kernel-weighted local polynomial smoothing had the smallest average percent deviations and average total absolute error. In this model and specification, health focus area-specific DAH fractions were independently estimated at the channel level after they were logit transformed. Time was the only independent variable included in the model. The health focus area-specific DAH estimates were adjusted so the sum of the channel's health focus area disbursements totaled the channel-specific DAH envelope. Our preferred model, the Epanechnikov kernel-weighted local polynomial smoothing, minimized both the average

percent deviation and the total absolute error out of sample, predicting two years ahead. See Dieleman et al. for a table that demonstrates the performance of four models, each with their optimal specification (as determined by the out-of-sample average percent deviation and total absolute error).[1]

World Health Organization's Global Health Observatory

WHO estimates health spending by source for 184 countries from 1995 to 2014. This database is updated annually and draws on publicly available documents from countries and international organizations such as National Health Accounts (NHAs), Ministry reports, and estimates from the World Bank and International Monetary Fund.

Data on government health spending as agent (GGHE/GHEA), prepaid private health spending (PPP), out-of-pocket (OOP), and gross domestic product (GDP) series were downloaded from the WHO database in current national currency units (NCUs) for all years and countries for which it was available. These datasets were formatted and merged with the IHME development assistance for health (DAH) data, extracted from the IHME *Financing Global Health* report and reported in 2015 USD, and IHME GDP per capita data, reported in 2015 purchasing-power parity and also 2015 US dollars. In addition, we extracted IMF deflator and exchange rate series whose imputation is described below. Each of the WHO health expenditure variables were divided by the WHO GDP series, and multiplied by the IHME GDP data reported in 2015 purchasing power parity dollars. The IHME DAH series was converted into 2015 purchasing-power parity dollars and then split into four variables – all DAH, DAH to governments, DAH to non-government entities, and unallocable DAH. The sum of DAH to governments, DAH to non-governments, DAH unallocable is all DAH.

In order to isolate domestically financed government health spending (GHES), DAH to governments that could be traced to a specific country was subtracted from the estimates of GHEA, and DAH to non-governments was subtracted from the estimates of PPP. The DAH estimates include general health sector support but do not capture un-earmarked all-sector development assistance that may have been spent to benefit the health sector. Data available from Open Aid show that between 2000 and 2013, less than five percent (4.57%) of official development assistance went toward general budget support.[67] Given that general budget support funds are further split across the various government sectors, it is unlikely that this gap in the DAH data has a substantive impact on our final health estimates.

Finally, lead and lag versions of each variable were generated and all the health expenditure variables were logit transformed. Prior to imputation, missingness in the health expenditure variables was approximately 2.3%. We use the Amelia package in R to impute missing values, which improves on mean imputation and single imputation and is specifically designed for cross-section longitudinal data such as our own.[68,69] The imputation was run for 100 iterations and included eight variables – country, year, OOP per GDP, all DAH per GDP, GHES per GDP, PPP per GDP, the natural log of GDP per capita, and logit transformed GGE per GDP – along with their lags and leads.

After imputation the data were once again aggregated, cleaned, transformed back to linear space, and multiplied by GDP. There was no missingness in this final dataset.

IMF, UN, PWT, and World Bank Data

We extracted deflator time-series data for 191 countries from 1980 to 2016 from the IMF World Economic Outlook database. For the years and countries of interest, the percent of missingness from this dataset was 2.4%. From the World Bank, deflator time-series data for 217 countries from 1960 to 2016 was extracted from the World Development Indicators database. The percent missingness for the years and countries of interest was 7.1%. From the UN National Accounts Main Aggregates database (UN), the IMF exchange rate series was extracted for the years 1970 to 2014 for 221 countries. Missingness among the years and countries of interest was 0.5%. From the PWT database, exchange rate series was extracted for 182 countries and 65 years – from 1950 through 2014. For the years and countries of interest, missingness was approximately 10.2%.

Deflator & exchange rate series

We downloaded deflator and exchange rate series from the WHO, WB, and IMF for all years and countries that were available. These data were formatted and combined with an IHME-generated GDP per capita series for 225 countries. Missingness in the data prior to imputation was approximately 14.7%. Leads and lags were generated for each of the variables before all the data were transformed into logit space. These 16 transformed variables – country, year, exchange rate from the World Bank in USD, exchange rate from the IMF in USD, exchange rate from the IMF in adjusted USD, exchange rate from PWT in USD, exchange rate from WHO in USD, exchange rate from the World Bank in purchasing-power parity dollars, exchange rate from the IMF in purchasing-power parity dollars, exchange rate from WHO in purchasing-power parity dollars, WHO price index, UN deflator, IMF deflator, World Bank deflator, GDP in 2010 purchasing-power parity dollars, GDP in 2010 USD – along with their leads and lags – were imputed using the R package Amelia. We converted the fractions to be imputed in logit space in order to ensure that the reverse transformation is between 0 and 1, and included them in three degrees of lags and leads each. The imputation was run for 50 iterations, after which the data were merged, cleaned, and transformed back into linear space. There was no missingness in the final series. The imputed IMF series for both the deflator and exchange rate were selected to be used exclusively in the rest of our analysis.

National Health Accounts

We used National Health Account (NHA) reports to track health spending by source (HF) and type (HC). The NHA data are reported in a standardized format, the System of Health Accounts (SHA) framework, created by the Organisation for Economic Co-operation and Development (OECD), World Health Organization (WHO), and Eurostat. Two iterations of the SHA framework have been developed, the first in 2001 and a second, updated version in 2011, referred to respectively as SHA 1.0 and SHA 2011. The transition between SHA 1.0 and SHA 2011 resulted in changed category classifications. Table 7 illustrates how we mapped the two frameworks so that we could compare country-years across SHA formats.

Table 7. SHA 1.0 to SHA 2011 mapping, for source (HF) and type (HC)

SHA 2011	Category	SHA 1.0
HF.1	GHES	HF.1
HF.2	PPP	HF.2.1 + HF.2.2 + HF.2.4 + HF.2.5
HF.3	OOP	HF.2.3
HF.4	DAH	HF.3
HC.1.1 + HC.2.1 + HC.3.1	Inpatient care, curative (HC.1.1), rehabilitative (HC.2.1) and long-term (HC.3.1)	HC.1.1 + HC.2.1 + HC.3.1
HC.1.2 + HC.1.3 + HC.2.2 + HC.2.3 + HC.3.2 + HC.3.3	Day and outpatient care, curative (HC.1.2, HC.1.3), rehabilitative (HC.2.2, HC.2.3), and long-term (HC.3.2, HC.3.3)	HC.1.2 + HC.1.3 + HC.2.2 + HC.2.3 + HC.3.2 + HC.3.3
HC.1.4 + HC.2.4 + HC.3.4	Home-based care, curative (HC.1.4), rehabilitative (HC.2.4), and long-term (HC.3.4)	HC.1.4 + HC.2.4 + HC.3.4
HC.4	Ancillary services	HC.4
HC.5	Medical goods	HC.5
HC.6.2 + HC.6.3	Immunization and early disease detection programs	HC.6.3 + HC.6.4
HC.7	Governance and health system and financing administration	HC.7
HC.9	Other	HC.nsk

We collected available NHA reports from WHO, OECD, Eurostat, and Global Health Data Exchange (GHDx) databases, as well as from a previous systematic review of NHA data by Bui et al. (2015).[70] Specifically, we extracted health spending reported by source and type of care. The source categories included government, prepaid private (PPP), out-of-pocket (OOP), and development assistance for health (DAH) spending. The type categories included inpatient care, day and outpatient care, ancillary services, medical goods, immunization and early disease-detection programs, governance and health system and financing administration, and other care. Inpatient and day and outpatient categories were aggregated across curative, rehabilitative, and long-term care. Box 1 below provides definitions for each type of care.[71–73]

Box 3. Definitions of type of care from SHA 1.0 and SHA 2011
**Inpatient care, curative & rehabilitative:** The treatment and/or care provided in a health care facility to patients formally admitted and requiring an overnight stay.<br>*SHA 1.0:* An in-patient is a patient who is formally admitted to an institution for treatment and/or care and stays for a minimum of one night in the hospital or other institution providing in-patient care. In-patient care is mainly delivered in hospitals, but partially also in nursing and residential care facilities or in establishments that are classified according to their focus of care under the ambulatory-care industry but perform in-patient care as a secondary activity.<br>*SHA 2011:* An inpatient contact comprises a formal admission into a health care facility for treatment and/or care that is expected to constitute an overnight stay. The classification as inpatient care is irrespective of the type of provider. Emergency cases and urgent admissions should be included only when they result in an overnight stay and formal admission to an inpatient facility, but are otherwise considered as outpatient cases.<br>**Outpatient care, curative & rehabilitative:** The medical and ancillary services delivered in a healthcare facility to a patient who is not formally admitted and does not stay overnight.<br>*SHA 1.0:* Out-patient care comprises medical and paramedical services delivered to out-patients. An out-patient is not formally admitted to the facility and does not stay overnight. An out-patient is thus a person who goes to a health care facility for a consultation/treatment, and who leaves the facility within several hours of the start of the consultation without being "admitted" to the facility as a patient. All visitors to ambulatory care facilities that are not day cases or over-the-night cases are considered out-patients.<br>*SHA 2011:* Outpatient care comprises medical and ancillary services delivered to a patient who is not formally admitted to a facility and does not stay overnight. An outpatient is thus a person who goes to a health care facility for a consultation or treatment, and who leaves the facility within hours of the start of the consultation without being "admitted" to the facility as a patient.<br>**Long-term care:** A range of medical and personal care services that are consumed with the primary goal of alleviating pain and suffering and reducing or managing the deterioration in health status in patients with a degree of long-term dependency.<br>*SHA 1.0:* Long-term health care comprises ongoing health and nursing care given to in-patients who need assistance on a continuing basis due to chronic impairments and a reduced degree of independence and activities of daily living. In-patient long-term care is provided in institutions or community facilities. Long-term care is typically a mix of medical (including nursing care) and social services. Only the former is recorded in the SHA under health expenditure.<br>*SHA 2011:* Long-term care consists of a range of medical and personal care services that are consumed with the primary goal of alleviating pain and suffering and reducing or managing the deterioration in health status in patients with a degree of long-term dependency. Long-term care includes medical or nursing care and personal care services. Social care services are excluded.<br>**Ancillary services:** The healthcare or long-term care related services nonspecified by function and nonspecified by mode of provision, which the patient consumes directly, in particular during an independent contact with the health system and that are not an integral part of a care service package, such as laboratory or imaging services or patient transportation and emergency rescue.

SHA 1.0: This item comprises a variety of services, mainly performed by paramedical or medical technical personnel with or without the direct supervision of a medical doctor, such as laboratory, diagnosis imaging, and patient transport.
SHA 2011: Ancillary services to health encompass a variety of services, mainly performed by paramedical or medical technical personnel with or without the direct supervision of a medical doctor. The only ancillary services to be reported separately are those that are directly requested by patients and not intermediate services. Diagnostic services within outpatient departments are usually part of the bundle of activities of treatment and are therefore not to be excluded.
Medical goods: Pharmaceutical products and non-durable medical goods intended for use in the diagnosis, cure, mitigation, or treatment of disease, including prescribed medicines and over-the-counter drugs, where the function and mode of provision are not specified.
SHA 1.0: Only the consumption of pharmaceuticals received from pharmacies or dispensing providers (including general retailers or mail-order) should be reported under the medical goods function; pharmaceuticals consumed in the course of a treatment, like surgery performed in an institutional or ambulatory setting, would not be included under medical goods.
SHA 2011: Includes medical goods acquired by the beneficiary either as a result of prescription following a health system contact or as a result of self-prescription; excludes medical goods consumed or delivered during a health care contact that are prescribed by a health care professional.
Immunization and early disease detection (EDD): Immunization includes both compulsory and voluntary immunizations/vaccinations, and can involve consumption by specific individuals in a campaign or in continued program operations; EDD can involve screening, diagnostic tests, and medical examinations to diagnose any communicable and non-communicable diseases.
SHA 1.0: No further definitions provided.
SHA 2011: For immunization, the expenditure involved in the consultation, both for the time and skills of the personnel and the purchase of the vaccine itself, should be accounted for; only disease detection before a diagnosis is made will be included in EDD, and self-examinations are not accounted for.
Governance, health system, and financing administration: Services that focus on the health system rather than direct health care, direct and support health system functioning, and are considered to be collective, as they are not allocated to specific individuals but benefit all health system users.
SHA 1.0: Health administration and health insurance are activities performed by private insurers and by central, regional, and local authorities including social security funds. They include the planning, management, regulation, and collection of funds and handling of claims of the delivery system. This excludes the administration of health care providers which is included in the valuation of the service functions.
SHA 2011: These expenditures direct and support health system functioning, and are incurred mostly but not exclusively by governments. Included are the formulation and administration of government policy; the setting of standards; the regulation, licensing or supervision of producers; management of the fund collection; and the administration, monitoring and evaluation of such resources, etc. However, some of these services are also provided by private entities, including by civil society (NGOs) and private medical insurance.
Other: Any other health care services not classified in the above function categories.

We identified 1,050 NHAs, both individually and from larger datasets. Of these 1,050 NHAs, health spending data by function and source was extracted from 964 across 112 countries and 26 years (1990–2015) (Table 8). The remaining 86 NHAs did not have data for any category but were included as country-years in the larger datasets. If we had selectively picked every country-year that had any health spending

data by function and source, these 86 would not have been included. The reason they did end up being included was because we imported large datasets rather than importing each specific country-year.

Not all NHAs were used for the THE or GHES analyses due to missingness. For example, if an NHA only reported spending for one health care function, the result would be that health spending for that country-year appears to be made up entirely of spending on that one category. So, if a country only reported medical goods spending, it would appear in our analysis that that country spent all of its health spending on medical goods for that year. Because of this limitation, we decided that at least inpatient and outpatient spending data had to be present for a country-year to be included in the analysis. We chose inpatient and outpatient as they make up a significant portion of health spending.

We also made restrictions on country-years included in the total health spending analysis. We decided that only country-years that reported GHES, PPP, OOP, and DAH (unless the country was high-income, in which case we assumed DAH to be zero) would be included for total health spending calculations. As such, a country-year may be represented in the GHES analysis but not the THE analysis if they reported GHES spending but not spending by the other sources.

Table 8. Country-years of NHA data available, by source.

ISO3	Year(s)	Source	ISO3	Year(s)	Source	ISO3	Year(s)	Source
AFG	2008, 2011	Bui et al.	FJI	2011–2014	WHO/GHDx	MNG	2002	Bui et al.
AFG	2012	WHO/GHDx	FRA	1995–2010	Bui et al.	MOZ	2004–2006	Bui et al.
ALB	2003	Bui et al.	FRA	2011–2015	OECD	MWI	2002, 2005–2008	Bui et al.
ARM	2011, 2012	WHO/GHDx	FSM	2005–2008	Bui et al.	MWI	2011	WHO/GHDx
AUS	1995–2010	Bui et al.	GAB	2011	WHO/GHDx	MYS	2013	WHO/GHDx
AUS	2011–2015	OECD	GBR	1995–2010	Bui et al.	NAM	1999–2006	Bui et al.
AUT	1995–2010	Bui et al.	GBR	2011–2015	OECD	NER	2011–2013	WHO/GHDx
AUT	2011–2015	OECD	GEO	2001–2009	Bui et al.	NGA	1998–2005	Bui et al.
BDI	2007	Bui et al.	GEO	2011	WHO/GHDx	NIC	1995–1999	Bui et al.
BDI	2012	WHO/GHDx	GHA	2012	WHO/GHDx	NLD	1995–2010	Bui et al.
BEL	1995–2010	Bui et al.	GRC	1995–2010	Bui et al.	NLD	2011–2015	OECD
BEL	2011–2015	OECD	GRC	2011–2015	OECD	NOR	1995–2010	Bui et al.
BEN	2012	WHO/GHDx	GTM	1995–1997	Bui et al.	NOR	2011–2015	OECD
BFA	2005	Bui et al.	HND	1998	Bui et al.	NPL	2006–2008	Bui et al.
BFA	2011, 2012	WHO/GHDx	HRV	2008–2014	Eurostat	NZL	1995–2010	Bui et al.
BGD	2007	Bui et al.	HTI	2010–2012	WHO/GHDx	NZL	2011–2015	OECD
BGD	2011, 2012	WHO/GHDx	HUN	1995, 1996, 1998–2010	Bui et al.	PER	1996	Bui et al.
BGR	2013	Eurostat	HUN	2011–2015	OECD	PLW	2007	Bui et al.
BOL	1995, 1996	Bui et al.	IDN	2009	Bui et al.	POL	1995–2010	Bui et al.
BRB	2012	WHO/GHDx	IND	2001, 2004	Bui et al.	POL	2011–2015	OECD
BTN	2009	Bui et al.	IND	2013	WHO/GHDx	PRT	1995–2010	Bui et al.
BWA	2007–2009	Bui et al.	IRL	1995–2010	Bui et al.	PRT	2011–2015	OECD
CAN	1995–-2010	Bui et al.	IRL	2011–2015	OECD	PSE	2000–2010	Bui et al.
CAN	2011–2015	OECD	IRN	2002–2008	Bui et al.	QAT	2009, 2010	Bui et al.

ISO3	Year(s)	Source	ISO3	Year(s)	Source	ISO3	Year(s)	Source
CHE	1995–2010	Bui et al.	ISL	1995–2010	Bui et al.	QAT	2011–2013	WHO/GHDx
CHE	2011–2015	OECD	ISL	2011–2015	OECD	ROU	2013	Eurostat
CHL	2003–2010	Bui et al.	ISR	1995–2010	Bui et al.	RWA	2002, 2003, 2006	Bui et al.
CHL	2012–2015	OECD	ISR	2011–2015	OECD	SEN	2005	Bui et al.
CIV	2007, 2008	Bui et al.	ITA	1995–2010	Bui et al.	SLE	2007–2010	Bui et al.
CMR	2011, 2012	WHO/GHDx	ITA	2011–2015	OECD	SLE	2013	WHO/GHDx
COD	2008	Bui et al.	JPN	1995–2010	Bui et al.	SLV	1995	Bui et al.
COD	2011–2013	WHO/GHDx	JPN	2011-–2015	OECD	SUR	2006	Bui et al.
COL	2000–2003	Bui et al.	KEN	2001, 2005, 2009	Bui et al.	SVK	1999–2010	Bui et al.
CPV	2008, 2009	Bui et al.	KGZ	2004, 2006–2009	Bui et al.	SVK	2011–2015	OECD
CPV	2011	WHO/GHDx	KHM	2012–2014	WHO/GHDx	SVN	1997–2010	Bui et al.
CYP	2000–2014	Eurostat	KIR	2007–2009	Bui et al.	SVN	2011–2015	OECD
CZE	1995–2010	Bui et al.	KOR	1990–2010	Bui et al.	SWE	1995–2010	Bui et al.
CZE	2011–2015	OECD	KOR	2011–2015	OECD	SWE	2011–2015	OECD
DEU	1995–2010	Bui et al.	LAO	2009	Bui et al.	SYC	2009	Bui et al.
DEU	2011–2015	OECD	LBR	2009	Bui et al.	SYC	2013	WHO/GHDx
DNK	1995–2010	Bui et al.	LIE	2000–2014	Eurostat	TGO	2008	Bui et al.
DNK	2011-–2015	OECD	LKA	1990–2006	Bui et al.	THA	2002–2008	Bui et al.
DOM	1996	Bui et al.	LKA	2013	WHO/GHDx	TJK	2013	WHO/GHDx
ECU	1995	Bui et al.	LTU	2011–2014	Eurostat	TUR	1995–2005	Bui et al.
EGY	1995, 2001	Bui et al.	LUX	1995–2010	Bui et al.	TWN	2009	Bui et al.
ESP	1995–2010	Bui et al.	LUX	2011–2015	OECD	TZA	2009	Bui et al.
ESP	2011–2015	OECD	LVA	2004–2015	OECD	TZA	2012	WHO/GHDx
EST	1999–2010	Bui et al.	MDA	2012	WHO/GHDx	UGA	1997	Bui et al.
EST	2011–2015	OECD	MDG	2003, 2007	Bui et al.	USA	1995–2010	Bui et al.
ETH	2004, 2007	Bui et al.	MEX	1999–2009	Bui et al.	USA	2011–2015	OECD
ETH	2010	WHO/GHDx	MEX	2011–2014	OECD	VUT	2005, 2007	Bui et al.
FIN	1995–2010	Bui et al.	MLI	1999–2004	Bui et al.	WSM	2002, 2004, 2006	Bui et al.
FIN	2011–2015	OECD	MMR	2002–2007	Bui et al.	ZMB	2002	Bui et al.
FJI	2007–2010	Bui et al.	MNE	2004–2006	Bui et al.			

Spending data were missing for at least one source by type of spending category for every country-year (Table 9). Additionally, some countries reported only high-level categories of spending while others reported only sub-categories of spending. Countries also sometimes provided spending estimates for categories without reporting the breakdown of spending for the sub-categories adding up to the larger category, e.g., a value for HC.4 (ancillary services) was reported but HC4.1 (laboratory services) was not. To ensure that we were including dollars reported in total amounts that were not elsewhere specified while also maximizing data in cases where only sub-categories were reported, we prioritized reported total amounts over sums of subtotal amounts. We did so by using the reported

totals where available and only substituting reported totals for a sum of reported subtotals when a reported total was missing.

Table 9. Country-years of data used in analysis, by spending source and by type category.

	GHES	PPP	OOP	DAH	Total spending
**Inpatient care, curative & rehabilitative**	643	476	495	751	631
**Outpatient care, curative & rehabilitative**	643	476	495	751	631
**Long-term care**	418	229	277	664	429
**Ancillary services**	555	372	420	712	559
**Medical goods**	593	429	474	726	605
**Immunization & early disease detection programs**	483	323	253	742	472
**Governance, health system, and financing administration**	618	432	258	741	617
**Other**	400	218	245	678	431

We included only country-years reporting at minimum inpatient and outpatient values when calculating fractions of each spending type by spending source. We did so to ensure a baseline denominator value. As a result, we excluded country-years that only reported one spending type and therefore would have had an inflated fraction value of 100% for that spending type over a given spending source.

Five country-years reported negative values for “Other” spending in the government health spending category. We replaced these five observations with zero, given the small size of the negative values compared to other categories.

We replaced missing values with zeroes in cases where a high-income country, as defined by the World Bank, reported missing for a DAH spending category. We did so under the assumption that high-income countries do not receive health assistance from abroad. When high-income countries did not report an overall spending total but did report GHES, PPP, and OOP spending, we substituted the total with the sum of GHES, PPP, and OOP values, again under the assumption that DAH was zero rather than missing. We left missing data for non-DAH values in high-income countries or for non-high income countries as missing in our final dataset.

Institute for Health Metrics and Evaluation’s GDP series

The Gross Domestic Product (GDP) series used in our analysis has been constructed from five different data sources. These sources include the Penn World Tables (PWT), the WB World Development Indicators, the United Nations Statistics Division (UNSTAT), the IMF World Economic Outlook report, and Angus Maddison’s research homepage at the University of Groningen Department of Economics. Applying several stages of least-squares and mixed effects regressions, these five series were filled out for 195 countries across 1950–2015, thereby removing any discontinuity or missingness. Following that, the IHME GDP series was constructed by taking an unweighted average of the filled series. Detailed explanation of this methodology can be found in James et al.[74]

Descriptive statistics

Table 10 presents descriptive statistics for variables used in the analyses.

Variable	Mean	Median	Standard deviation	Minimum	Maximum
THE per capita	1,028.78	471.40	1,376.05	8.75	9,237.13
GDP per capita	15,582.54	8,515.59	18,778.95	317.23	12,9207.40
**By source**					
GHES/THE	0.52	0.54	0.22	0.00	0.97
PPP/THE	0.06	0.04	0.08	0.00	0.65
OOP/THE	0.34	0.31	0.19	0.02	0.95
DAH/THE	0.08	0.01	0.14	0.00	0.97
**By type**					
Inpatient care, curative & rehabilitative/THE	0.29	0.29	0.08	0.03	0.60
Outpatient care, curative & rehabilitative/THE	0.30	0.29	0.10	0.02	0.72
Long-term care/THE	0.09	0.08	0.07	0.00	0.29
Ancillary services/THE	0.05	0.05	0.04	0.00	0.42
Medical goods/THE	0.22	0.20	0.11	0.00	0.60
Immunization & early disease detection programs/THE	0.04	0.03	0.04	0.00	0.38
Governance, health system, and financing administration/THE	0.05	0.04	0.05	0.00	0.54
Other/THE	0.02	0.01	0.06	0.00	0.48
Inpatient care, curative & rehabilitative/GHES	0.36	0.36	0.12	0.00	0.85
Outpatient care, curative & rehabilitative/GHES	0.27	0.26	0.10	0.00	0.72
Long-term care/GHES	0.10	0.10	0.08	0.00	0.30
Ancillary services/GHES	0.05	0.04	0.04	0.00	0.19
Medical goods/GHES	0.13	0.12	0.08	0.00	0.44
Immunization & early disease detection programs/GHES	0.08	0.04	0.11	0.00	0.75
Governance, health system, and financing administration/GHES	0.07	0.04	0.10	0.00	0.85
Other/GHES	0.03	0.01	0.07	0.00	0.60

SECTION 3. STATISTICAL MODEL

Using the data on health financing by source and by type, merged with the gross domestic product per capita (GDP per capita), described in the previous sections, two primary analyses were conducted. These analyses, described below, use penalized spline (P-spline) smoothing estimation to estimate total health spending and its components across all years and countries. This methodological decision was based upon the desire to describe the relationship between development (as measured by logged gross domestic product per capita) and health spending across countries and years. Thus, although country fixed effects and panel analyses were conducted in the exploratory analyses, these were not used in the final analysis presented in this study as they would ultimately remove the cross-country-year trends we attempt to describe. Penalized splines are flexible, nonlinear multivariate regressions that allow us to capture the cross-country-year trends of interest in this study.

Furthermore, our analysis excludes four countries from the Global Burden of Disease list of 188 countries (North Korea, Palestine, Taiwan, and Zimbabwe) due to missingness of data from either WHO (regarding health expenditures) and/or the IMF (regarding the government expenditure, deflator, and exchange rates). Palestine and Taiwan were excluded due to lack of health expenditure data, while Zimbabwe did not have complete and reliable deflators or exchange rate series. North Korea was excluded for not having either all-sector government expenditure, health expenditure, or any of the conversion rates.

By source

Total health spending (THE) by source was broken down into four components:

1. Government health spending as source (GHES)
2. Out-of-pocket household health spending (OOP)
3. Prepaid private health schemes (PPP)
4. Development assistance for health (DAH)

These data span 1995 to 2014 and are complete panel datasets for 184 countries providing 3,680 country-years. To estimate the value of THE and the proportion that each of its four components made up at every potential level of development observed in our panel, controlling for year, we modeled each separately using a generalized additive model with an integrated penalized spline (P-spline) smoothing estimation analysis with logged GDP per capita and year as independent continuous variables. Our dataset contains 881 country-years where DAH equaled zero; 96% of these zero values were in high-income countries. It was determined that a log-transformation allowed for a better data fit, but to not lose country-years in our analysis, we first “lemon-squeezed” each component and then constrained the sum of the proportions to equal one using a center log-ratio transformation (CLR).[75–77] To estimate confidence intervals, we took 1,000 bootstrapped samples (clustering over countries), then analyzed each sample and predicted each outcome variable for every value of logged GDP per capita (rounded to a tenth). The following equations walk through an example of how one component, DAH, is transformed and modeled.

Lemon-squeeze transformation (LS)

$$LS(DAH_{c,t}) = \frac{\left((DAH_{c,t}) * (N_{DAH_{c,t}} - 1) + 0.5\right)}{N_{DAH_{c,t}}})$$

Center log-ratio transformation (CLR)

$$CLR\left(LS(DAH_{c,t})\right) = natural - \log(\frac{LS(DAH_{c,t})}{(LS(DAH_{c,t}) * LS(OOP_{c,t}) * LS(PPP_{c,t}) * LS(GHE - S_{c,t}))^{1/4}})$$

Generalized additive model with integrated penalized spline smoothing estimation

$$E(CLR\left(LS(DAH_{c,t})\right) = \alpha + f(\ln GDP\, pc_{c,t}) + f(year)$$

Where *f*() represents the penalized spline smoothing function, *N* represents the number of observations for the particular spending component (DAH in this example), *c* indicates country, and *t* represents time. The P-spline analysis was conducted in R using the gam function within the mgcv library.[78]

To reiterate, once the P-spline models were estimated, we predicted for every observed value of logged GDP per capita in 2014 (rounded to the tenth) while holding year constant for each 1,000 bootstrapped sample. We then collated these predictions and took the mean at each estimate of logged GDP per capita. To generate 95% uncertainty intervals, we also took the 2.5 and 97.5 percentiles of the predictions at each respective value of logged GDP pc.

By type

The by-type analysis nearly mirrors the by source analysis outlined above except instead of four components, the analysis is conducted for eight components of total health spending:

1. Inpatient care, curative and rehabilitative
2. Day & outpatient care, curative and rehabilitative
3. Long-term care
4. Ancillary services
5. Medical goods
6. Immunization & early disease detection
7. Governance, health system, and financing administration
8. Other

Missingness of the health spending by type data is described in Table 9 above. To estimate the value of the proportion that each of these eight components made up at every potential level of development observed in our panel, we modeled each separately using a generalized additive model with an integrated penalized spline (P-spline) smoothing estimation analysis with logged GDP per capita and year as independent variables. It was determined that a log-transformation allowed for a better data fit, but to not lose country-years in our analysis, we first "lemon-squeezed" each component and then constrained the sum of the proportions to equal 1 using a center log-ratio transformation (CLR).[75–77] We then predicted each of our eight outcome variables for every value of logged GDP per capita (rounded to a tenth). The following equations walk through an example of how one component, Ancillary services, is transformed and modeled.

Lemon-squeeze transformation (LS)

$$LS\left(Ancillary_{c,t}\right) = \frac{\left(Ancillary_{c,t}\right) * \left(N_{Ancillary_{c,t}} - 1\right) + 0.5}{N_{Ancillary_{c,t}}})$$

Center log-ratio transformation (CLR)

$$CLR\left(LS\left(Ancillary_{c,t}\right)\right) = natural - \log(LS\left(Ancillary_{c,t}\right) / \left[LS\left(Ancillary_{c,t}\right) * LS\left(Inpatient_{c,t}\right) * LS\left(Outpatient_{c,t}\right) * LS\left(Longterm_{c,t}\right) * LS\left(Medial\ Goods_{c,t}\right) * LS\left(Immunization_{c,t}\right) * LS\left(Governance_{c,t}\right) * LS\left(Other_{c,t}\right)\right]^{1/8})$$

Generalized additive model with integrated penalized spline smoothing estimation

$$E(CLR\left(LS\left(Ancillary_{c,t}\right)\right) = \alpha + f\left(\ln GDP\ pc_{c,t}\right) + f(year)$$

Where *f*() represents the penalized spline smoothing function, *N* represents the number of observations for the particular spending component (Ancillary care in this example), *c* indicates country and *t* represents time.

To reiterate, once the P-spline models were estimated, we predicted for every observed value of logged GDP per capita (rounded to the tenth) while holding year constant.

Robustness analyses

In addition to the analyses presented above, we also conducted two additional penalized spline models to estimate the total health spending and the by-source components of total health spending – government health spending as source, out-of-pocket health spending, prepaid private health spending, and development assistance for health. These two additional models used substitutes for the logged gross domestic product (GDP) per capita. One model used Socio-demographic Index (SDI), while the other used the UN's Human Development Index (HDI).[79] The Socio-demographic Index (SDI) data were extracted from the Institute for Health Metrics and Evaluation's Global Burden of Disease study 2015, and were available for all countries and years included in this study.[80] SDI is a composite of gross domestic product per person, educational attainment per person, and the inverse of total fertility rate. Similar to SDI, HDI is a composite index of education, life expectancy, and per capita income. All other steps in the analysis were completed exactly as described in the "By source" section above.

We also present additional robustness analyses: (1) as previously mentioned, components of health spending by source are imputed; to verify that our estimates are not biased by these imputations we rerun the analysis without these imputed values; (2) hold constant GDP per capita and examine the relationship between time and health spending at the median value of GDP pc; (3) instead of using the penalized spline smoothing analysis, we use a multivariate locally weighted smoothing model (Loess) to validate our estimates; (4) validate the relationship between GDP per capita and health spending using both a sub-sample panel dataset as well as a cross-sectional dataset.

Socio-demographic Index as a predictor

The following equation and figures show the use of Socio-demographic Index (SDI) as a predictor of total health spending and the by-source components of that spending.

Generalized additive model with integrated penalized spline smoothing estimation using SDI. Example of development assistance for health.

$$E(CLR\left(LS(DAH_{c,t})\right) = \alpha + f(SDI_{c,t}) + f(year)$$

Figure 1. Total health spending per capita by Socio-demographic Index, 2014

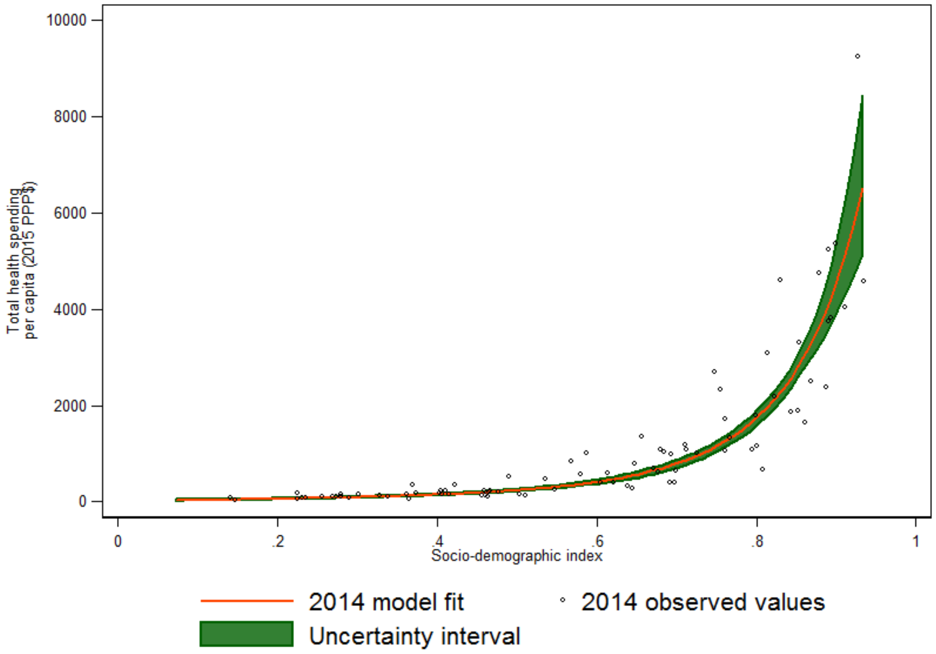

Figure 2. Total health spending per gross domestic product by Socio-demographic Index, 2014

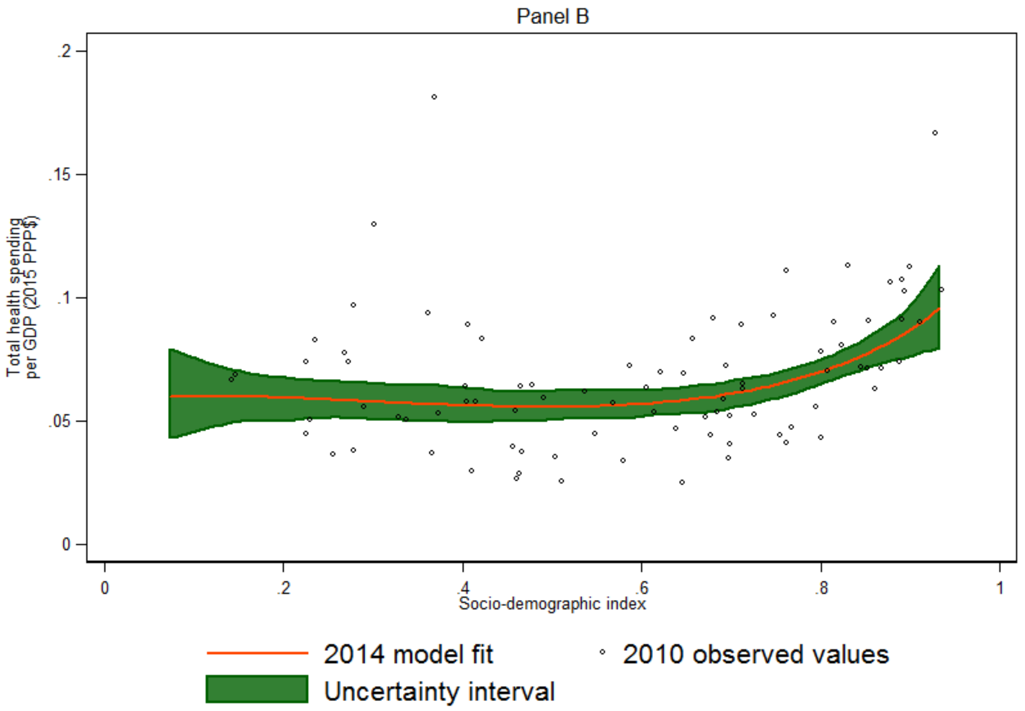

Figure 3. Percent of health care spending by source and Socio-demographic Index (SDI)

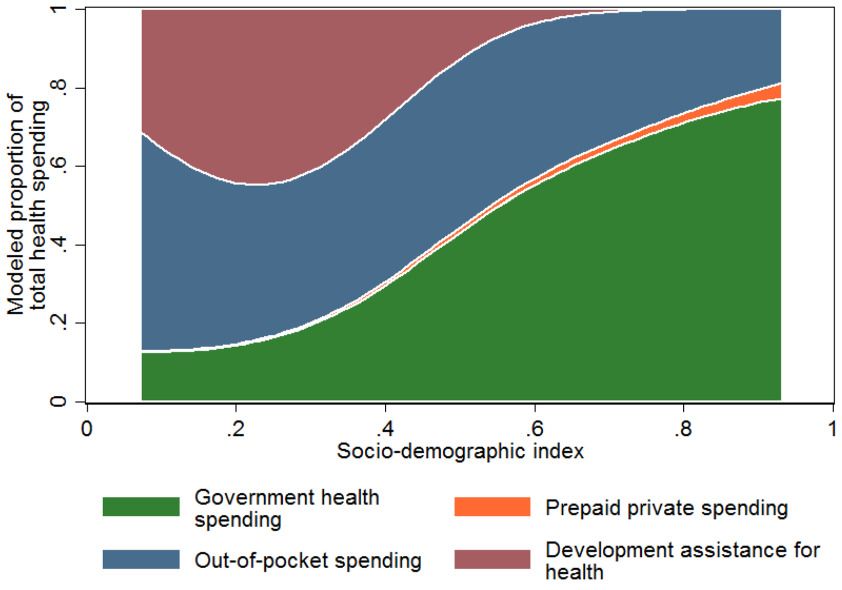

Figure 4. Health spending per capita relative to Socio-demographic Index

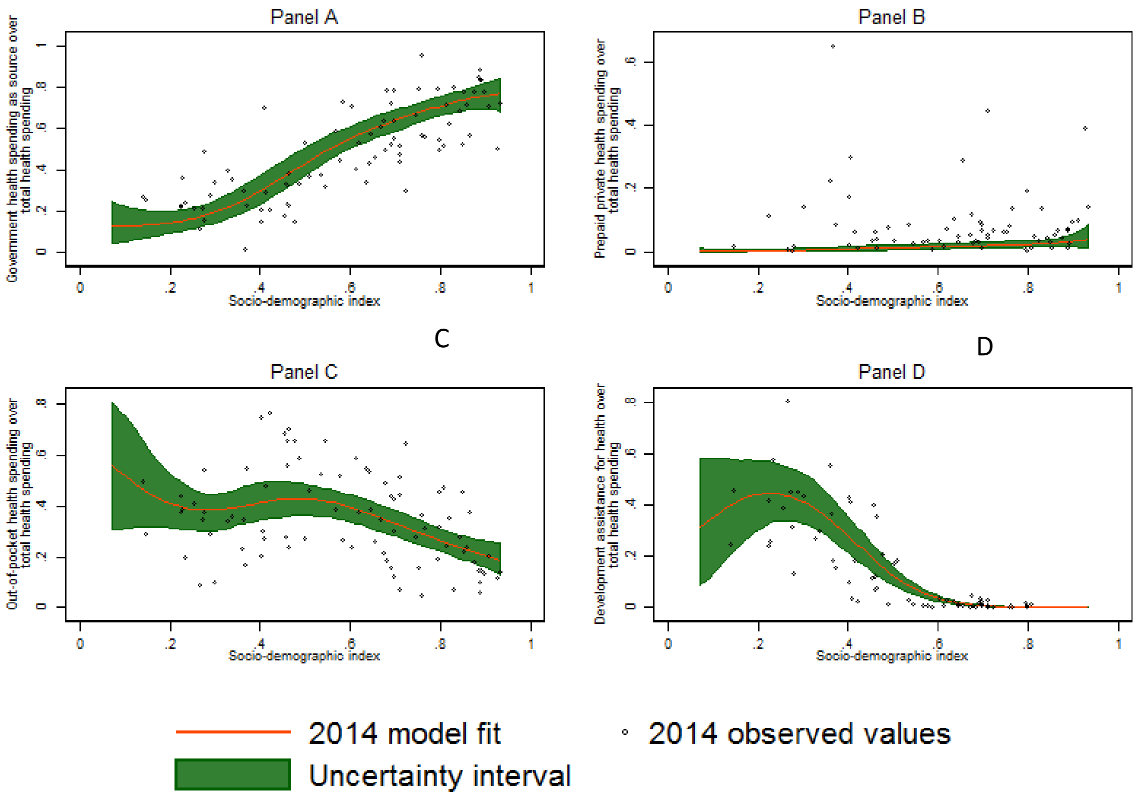

Per capita spending on health relative to Socio-demographic Indices for government (A), pre-paid private (B), and out-of-pocket (C) spending, and development assistance for health (D). All graphs include only countries with a population of at least 30 million.

Human Development Index as a predictor

The following equation and figures show the use of the UN's Human Development Index (HDI) as a predictor of total health spending and the by-source components of that spending.

Generalized additive model with integrated penalized spline smoothing estimation using HDI. Example of development assistance for health.

$$E(CLR\left(LS(DAH_{c,t})\right) = \alpha + f\left(HDI_{c,t}\right) + f(year)$$

Figure 5. Total health spending per capita by Human Development Index, 2014

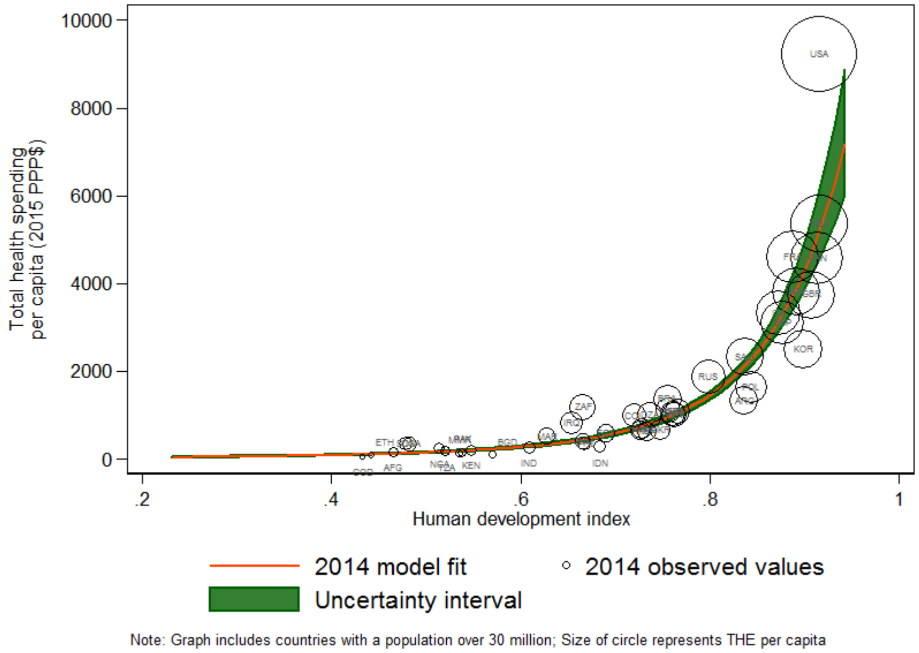

Figure 6. Percent of health care spending by source and Human Development Index

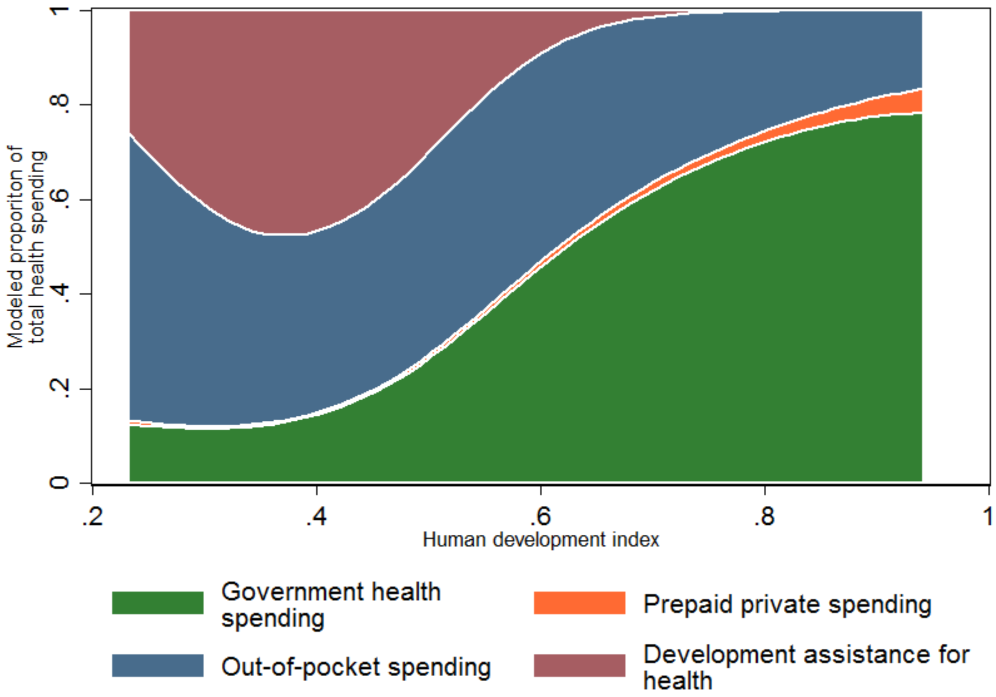

Figure 7. Health spending per capita relative to Human Development Index

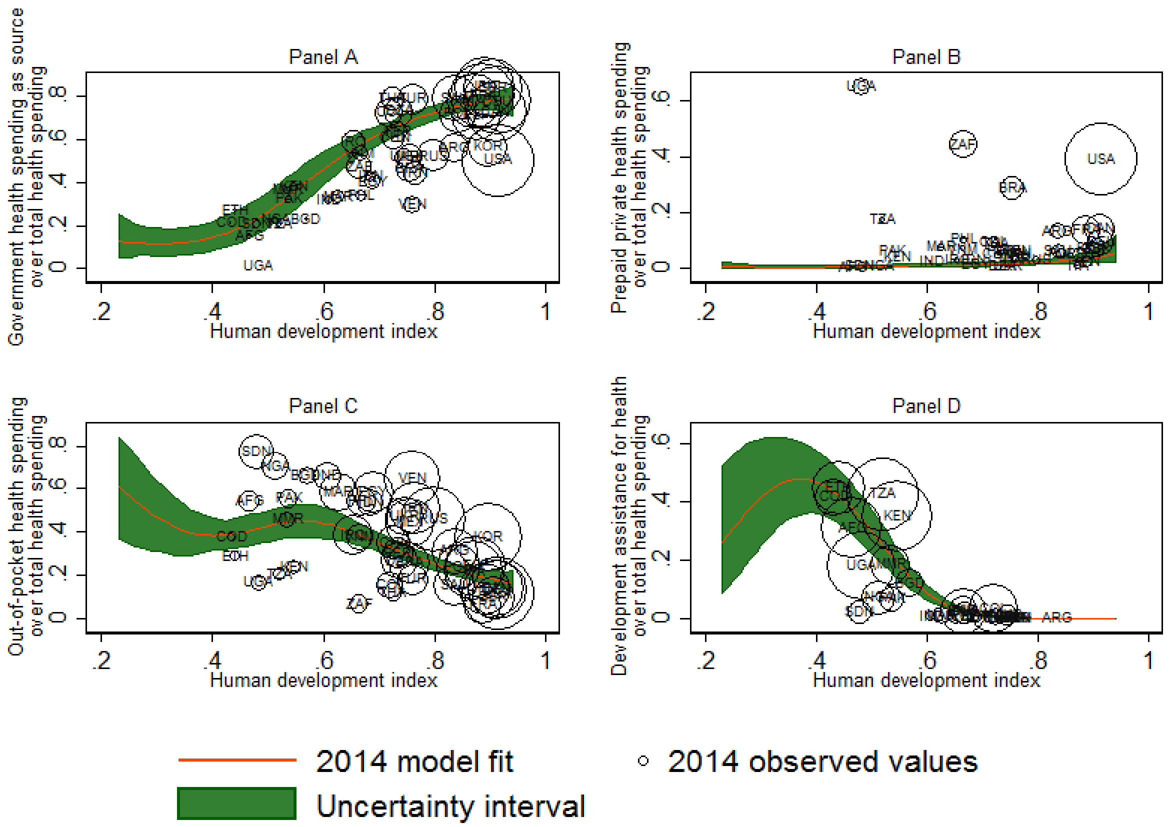

Note: Graph includes countries with a population over 30 million; Size of circle represents respective component per capita

Per capita spending on health relative to Human Development Indices for government (A), pre-paid private (B), and out-of-pocket (C) spending, and development assistance for health (D). Circle size indicates total health spending per capita. All graphs include only countries with a population of at least 30 million.

Penalized spline by source analysis without imputed values

As previously described in the World Health Organization's Global Health Expenditure Observatory data section above, components of health spending by source are imputed. To verify that our final estimates are not biased by these imputations, we rerun the analysis without these imputed value using logged GDP pc and year in one model and SDI and year in a second model. The results from these analyses are presented below.

Figure 8. Percent of health care spending by source and gross domestic product per capita, without imputed data

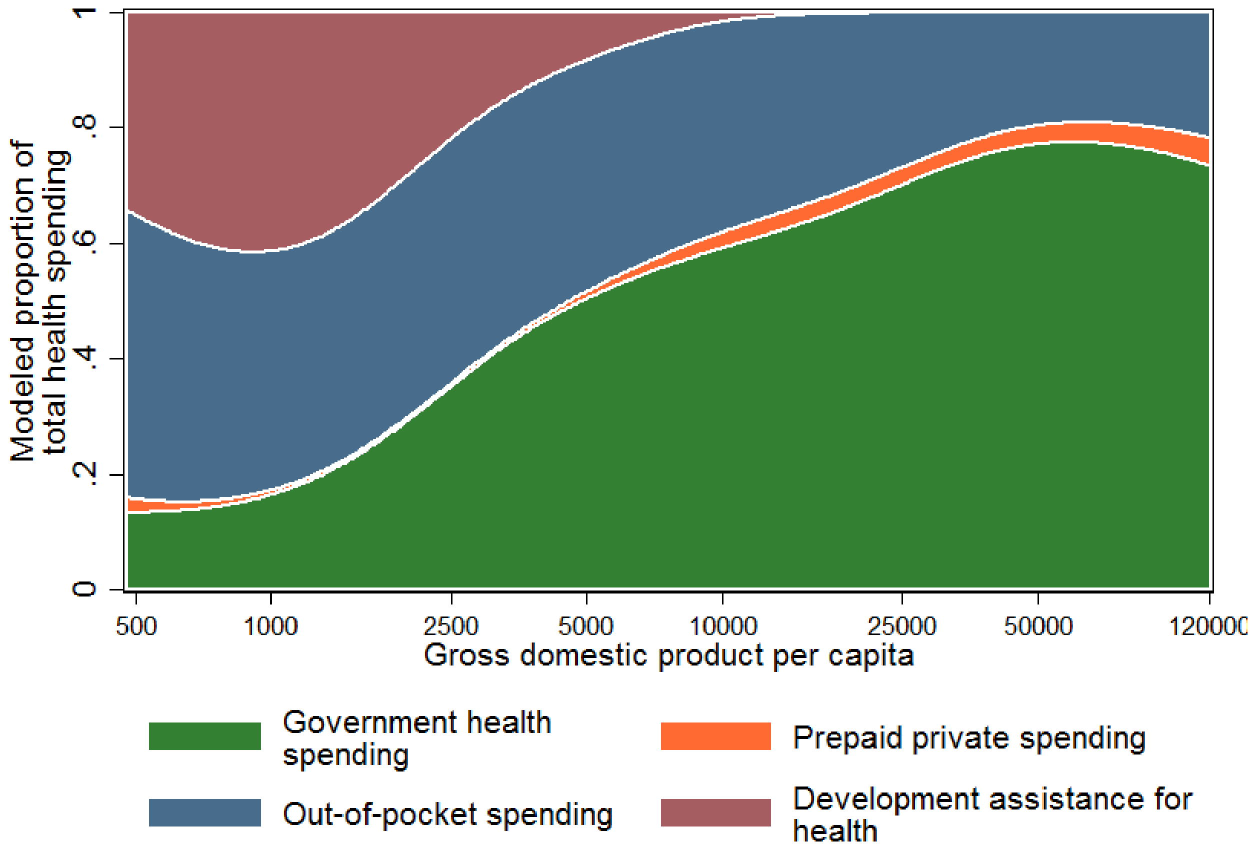

Figure 9. Health spending per capita relative to gross domestic product per capita, without imputed data

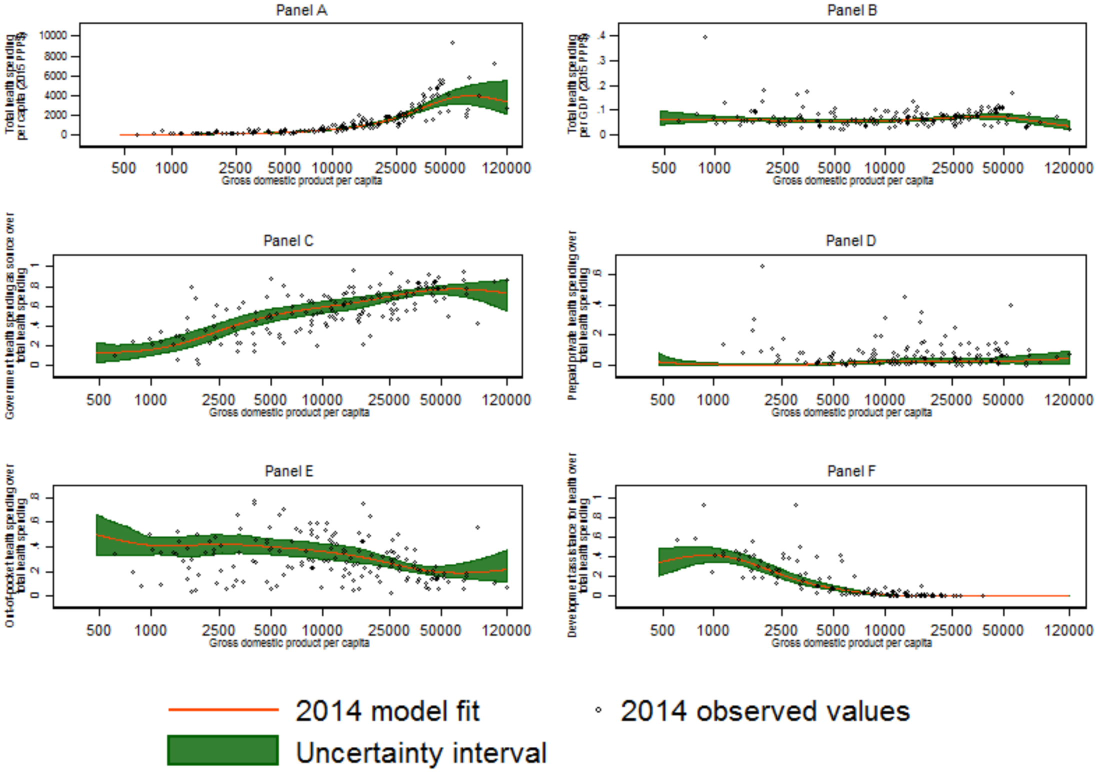

Per capita spending on health relative to gross domestic product per capita for total health spending per capita (A), total health spending per gross domestic product (B), government (C), pre-paid private (D), and out-of-pocket (E) spending, and development assistance for health (F).

Figure 10. Percent of health care spending by source and Socio-demographic Index, without imputed data

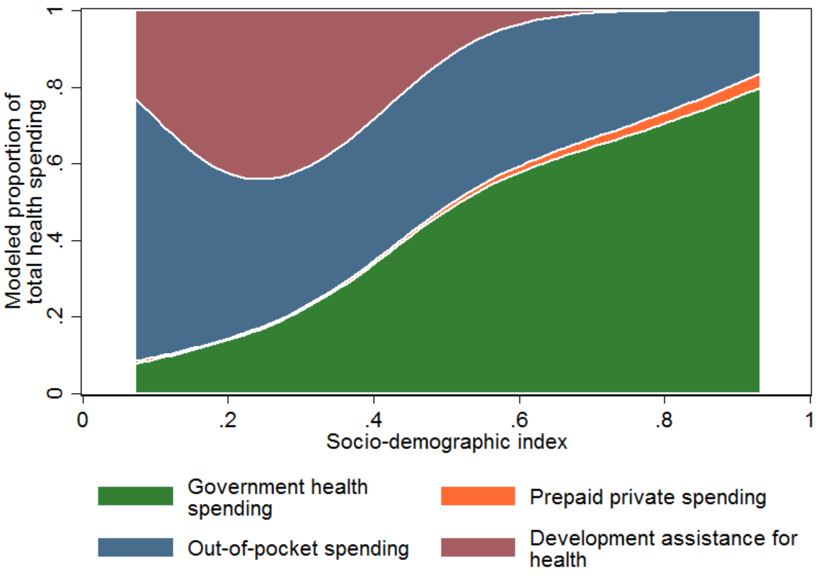

Figure 11. Health spending per capita relative to Socio-demographic Index, without imputed data

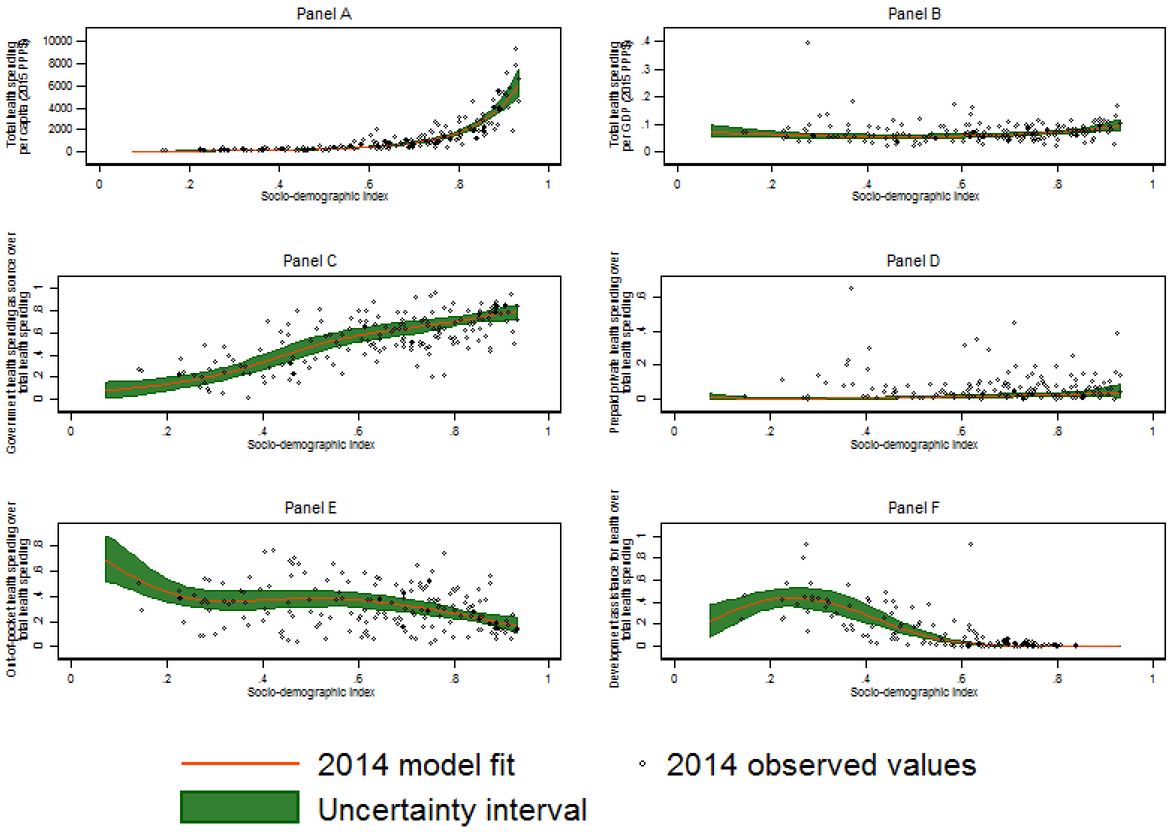

Per capita spending on health relative to Socio-demographic Index for total health spending per capita (A), total health spending per gross domestic product (B), government (C), pre-paid private (D), and out-of-pocket (E) spending, and development assistance for health (F).

Time as a predictor

The main results presented in the paper adjust for GDP per capita and time. Figures 12 and 13 below show the output from these same regressions to see changes in time when holding the GDP per capita constant at the median value ($8,703.93) observed in the data. The figure highlights that over time there is very little variation across the makeup of health spending by source, when holding GDP per capita constant.

Figure 12. Composition of total health spending by source and time

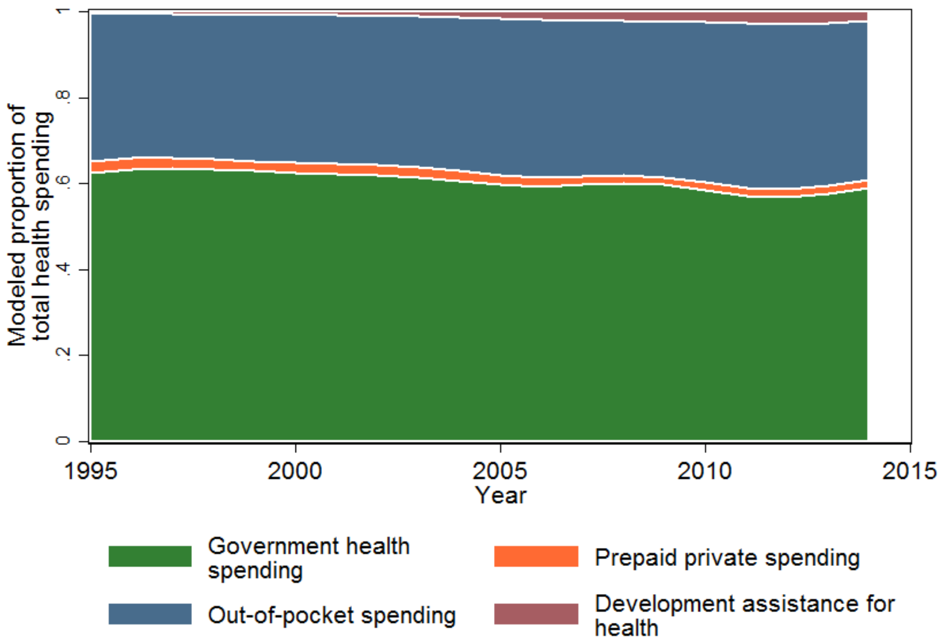

Figure 13. Total health spending per person, per GDP and percent of health care spending by source and time

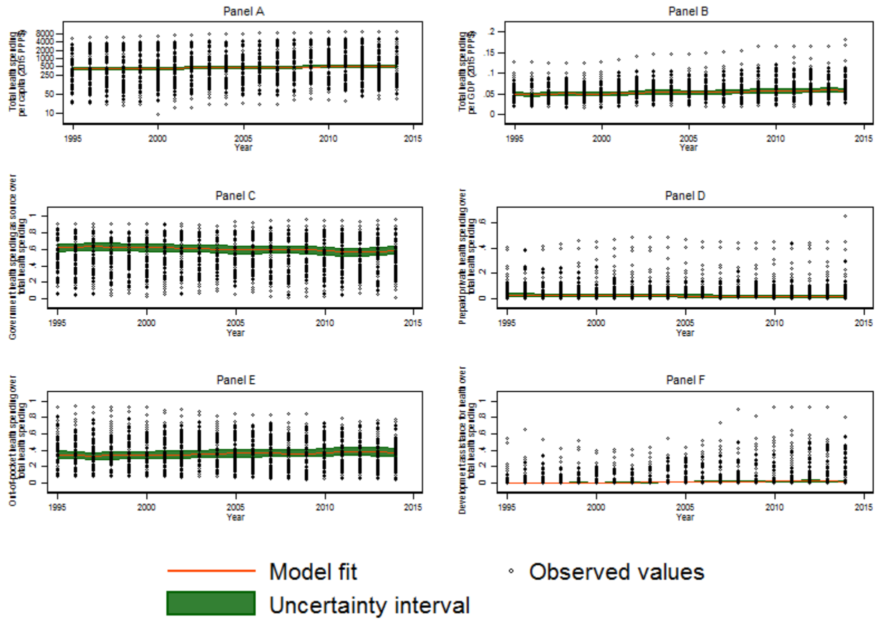

Multivariate locally weighted smoothing analysis

We also conducted the same analysis as described in the main paper and in the by-source section of the appendix, but instead of a penalized spline estimation method, we used a multivariate locally weighted smoothing (Loess) estimation method, with predictors of logged GDP per capita and year in one model and SDI and year in another model. The following figures and Table 11 provide the estimates from this multivariate Loess analysis, which was conducted using R, Loess command.

Figure 14. Total health spending per capita by gross domestic product per capita, 2014

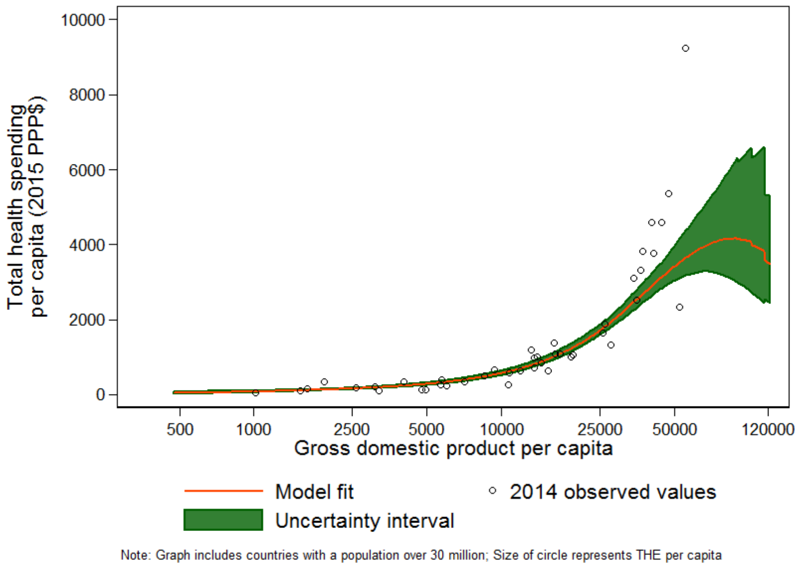

Figure 15. Percent of health care spending by source and gross domestic product per capita

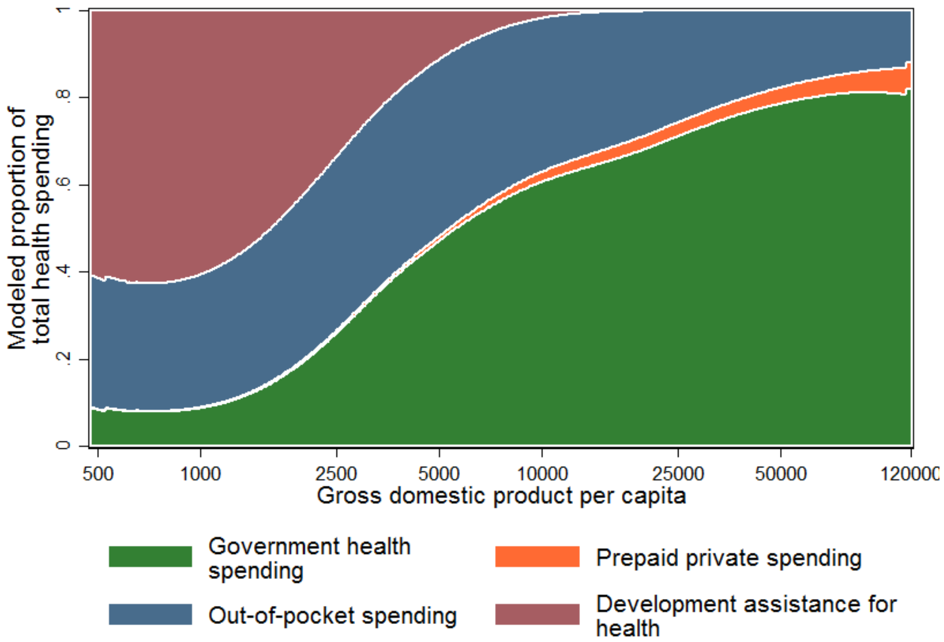

Figure 16. Total health spending per capita by Socio-demographic Index, 2014

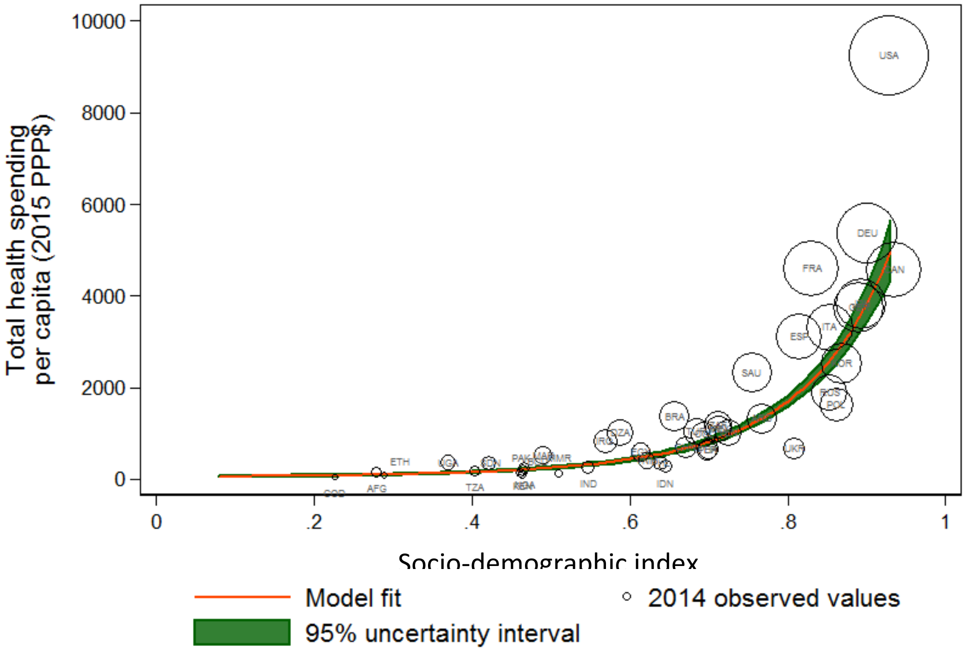

Note: Graph includes countries with a population over 30 million; Size of circle represents THE per capita

Figure 17. Percent of health care spending by source and Socio-demographic Index

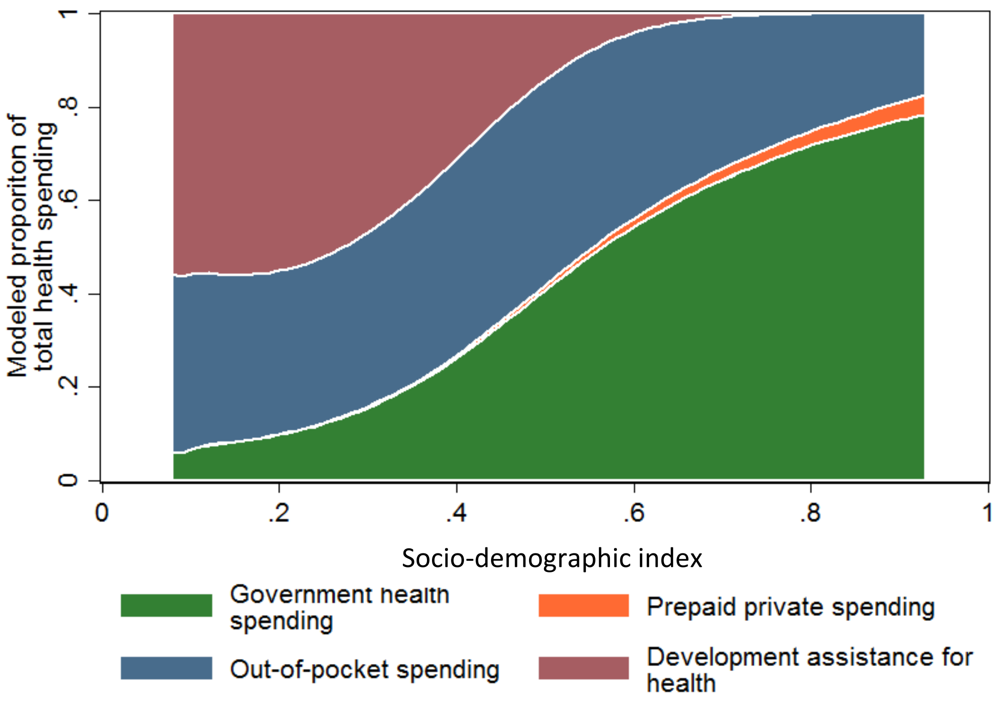

Examine exponential relationship between GDP per capita and health spending

To test if national health spending per capita is exponentially related to GDP per capita, we regressed the natural logarithmic transformed THE per capita on natural logarithmic transformed GDP per capita, using ordinary least squares regression. We did this using the full panel of 20 years and 184 countries and using only the 2014 data (only 184 countries). Both regressions show an exceptionally strong exponential relationship.

Full panel:

Source	SS	df	MS
Model	6105.96993	1	6105.96993
Residual	664.823473	3678	.18075679
Total	6770.79341	3679	1.84038962

Number of obs	= 3680
F(1, 3678)	=33780.03
Prob > F	= 0.0000
R-squared	= 0.9018
Adj R-squared	= 0.9018
Root MSE	= .42516

ln_thepc	Coef.	Std. Err.	t	P>|t|	[95% Conf.	Interval]
ln_gdppc	1.027228	.005589	183.79	0.000	1.01627	1.038186
_cons	-3.095358	.0506146	-61.16	0.000	-3.194593	-2.996122

Cross-section:

Source	SS	df	MS
Model	268.840549	1	268.840549
Residual	36.3640483	182	.199802463
Total	305.204597	183	1.66778469

Number of obs	= 184
F(1, 182)	= 1345.53
Prob > F	= 0.0000
R-squared	= 0.8809
Adj R-squared	= 0.8802
Root MSE	= .44699

ln_thepc	Coef.	Std. Err.	t	P>|t|	[95% Conf.	Interval]
ln_gdppc	.9999202	.0272595	36.68	0.000	.9461348	1.053706
_cons	-2.7445	.2532966	-10.84	0.000	-3.244276	-2.244725

SECTION 4. SUPPLEMENTARY TABLES & FIGURES

Table 11. Estimates of health spending by gross domestic product per capita based on Multivariate Loess Estimation Method

Gross domestic product per capita (2015 PPP$)	Natural log of gross domestic product per capita (2015 PPP$)	Total health spending per capita (2015 PPP$)	Domestic government health spending per total health spending (proportion)	Out-of-pocket spending per total health spending (proportion)	Prepaid private spending per total health spending (proportion)	Development assistance for health per total health spending (proportion)
**476.37**	6.17	39.88	0.09	0.30	0.00	0.61
**481.16**	6.18	40.26	0.09	0.30	0.00	0.61
**486.00**	6.19	40.64	0.09	0.30	0.00	0.61
**490.88**	6.20	41.03	0.08	0.30	0.00	0.61
**495.81**	6.21	41.42	0.08	0.30	0.00	0.61
**500.80**	6.22	41.81	0.08	0.30	0.00	0.61
**505.83**	6.23	42.21	0.08	0.30	0.00	0.62
**510.91**	6.24	42.61	0.08	0.30	0.00	0.62
**516.05**	6.25	43.01	0.08	0.30	0.00	0.62
**521.23**	6.26	43.42	0.08	0.30	0.00	0.62
**526.47**	6.27	42.79	0.09	0.30	0.00	0.61
**531.76**	6.28	43.20	0.09	0.30	0.00	0.61
**537.11**	6.29	43.62	0.09	0.30	0.00	0.61
**542.51**	6.30	44.04	0.09	0.30	0.00	0.61
**547.96**	6.31	44.46	0.08	0.30	0.00	0.61
**553.47**	6.32	44.89	0.08	0.30	0.00	0.62
**559.03**	6.33	45.31	0.08	0.30	0.00	0.62
**564.65**	6.34	45.75	0.08	0.30	0.00	0.62
**570.32**	6.35	46.18	0.08	0.30	0.00	0.62
**576.05**	6.36	46.62	0.08	0.30	0.00	0.62
**581.84**	6.37	47.06	0.08	0.30	0.00	0.62
**587.69**	6.38	47.51	0.08	0.30	0.00	0.62
**593.60**	6.39	47.96	0.08	0.30	0.00	0.62
**599.56**	6.40	48.41	0.08	0.30	0.00	0.62
**605.59**	6.41	48.87	0.08	0.30	0.00	0.62
**611.67**	6.42	49.46	0.08	0.29	0.00	0.63
**617.82**	6.43	49.79	0.08	0.29	0.00	0.62
**624.03**	6.44	50.26	0.08	0.29	0.00	0.62
**630.30**	6.45	50.73	0.08	0.29	0.00	0.62
**636.64**	6.46	51.20	0.08	0.29	0.00	0.63
**643.04**	6.47	51.75	0.08	0.29	0.00	0.62
**649.50**	6.48	52.23	0.08	0.29	0.00	0.62

Gross domestic product per capita (2015 PPP$)	Natural log of gross domestic product per capita (2015 PPP$)	Total health spending per capita (2015 PPP$)	Domestic government health spending per total health spending (proportion)	Out-of-pocket spending per total health spending (proportion)	Prepaid private spending per total health spending (proportion)	Development assistance for health per total health spending (proportion)
**656.03**	6.49	52.72	0.08	0.29	0.00	0.62
**662.62**	6.50	53.20	0.08	0.29	0.00	0.62
**669.28**	6.51	53.69	0.08	0.29	0.00	0.62
**676.00**	6.52	54.19	0.08	0.29	0.00	0.63
**682.80**	6.53	54.69	0.08	0.29	0.00	0.63
**689.66**	6.54	55.19	0.08	0.29	0.00	0.63
**696.59**	6.55	55.69	0.08	0.29	0.00	0.63
**703.59**	6.56	56.20	0.08	0.29	0.00	0.63
**710.66**	6.57	56.72	0.08	0.29	0.00	0.63
**717.81**	6.58	57.23	0.08	0.29	0.00	0.63
**725.02**	6.59	57.75	0.08	0.29	0.00	0.63
**732.31**	6.60	58.28	0.08	0.29	0.00	0.63
**739.67**	6.61	58.81	0.08	0.29	0.00	0.63
**747.10**	6.62	59.34	0.08	0.29	0.00	0.63
**754.61**	6.63	59.88	0.08	0.29	0.00	0.63
**762.19**	6.64	60.42	0.08	0.29	0.00	0.63
**769.85**	6.65	60.96	0.08	0.29	0.00	0.62
**777.59**	6.66	61.51	0.08	0.29	0.00	0.62
**785.40**	6.67	62.06	0.08	0.29	0.00	0.62
**793.30**	6.68	62.61	0.08	0.29	0.00	0.62
**801.27**	6.69	63.17	0.08	0.30	0.00	0.62
**809.32**	6.70	63.74	0.08	0.30	0.00	0.62
**817.46**	6.71	64.31	0.08	0.30	0.00	0.62
**825.67**	6.72	64.88	0.08	0.30	0.00	0.62
**833.97**	6.73	65.45	0.08	0.30	0.00	0.62
**842.35**	6.74	66.03	0.08	0.30	0.00	0.62
**850.82**	6.75	66.62	0.08	0.30	0.00	0.62
**859.37**	6.76	67.21	0.08	0.30	0.00	0.62
**868.01**	6.77	67.80	0.08	0.30	0.00	0.62
**876.73**	6.78	68.40	0.08	0.30	0.00	0.62
**885.54**	6.79	69.00	0.08	0.30	0.00	0.62
**894.44**	6.80	69.60	0.08	0.30	0.00	0.62
**903.43**	6.81	70.21	0.08	0.30	0.00	0.62
**912.51**	6.82	70.83	0.08	0.30	0.00	0.61
**921.68**	6.83	71.44	0.08	0.30	0.00	0.61

Gross domestic product per capita (2015 PPP$)	Natural log of gross domestic product per capita (2015 PPP$)	Total health spending per capita (2015 PPP$)	Domestic government health spending per total health spending (proportion)	Out-of-pocket spending per total health spending (proportion)	Prepaid private spending per total health spending (proportion)	Development assistance for health per total health spending (proportion)
**930.94**	6.84	72.07	0.08	0.30	0.00	0.61
**940.30**	6.85	72.69	0.08	0.30	0.00	0.61
**949.75**	6.86	73.32	0.09	0.30	0.00	0.61
**959.30**	6.87	73.96	0.09	0.30	0.00	0.61
**968.94**	6.88	74.60	0.09	0.30	0.00	0.61
**978.67**	6.89	75.25	0.09	0.30	0.00	0.61
**988.51**	6.90	75.89	0.09	0.30	0.00	0.61
**998.45**	6.91	76.55	0.09	0.31	0.00	0.61
**1,008.48**	6.92	77.21	0.09	0.31	0.00	0.60
**1,018.62**	6.93	77.87	0.09	0.31	0.00	0.60
**1,028.85**	6.94	78.54	0.09	0.31	0.00	0.60
**1,039.19**	6.95	79.21	0.09	0.31	0.00	0.60
**1,049.64**	6.96	79.89	0.09	0.31	0.00	0.60
**1,060.19**	6.97	80.57	0.09	0.31	0.00	0.60
**1,070.84**	6.98	81.26	0.09	0.31	0.00	0.60
**1,081.60**	6.99	81.95	0.09	0.31	0.00	0.59
**1,092.47**	7.00	82.64	0.09	0.31	0.00	0.59
**1,103.45**	7.01	83.34	0.09	0.31	0.00	0.59
**1,114.54**	7.02	84.05	0.10	0.31	0.00	0.59
**1,125.74**	7.03	84.76	0.10	0.31	0.00	0.59
**1,137.06**	7.04	85.48	0.10	0.32	0.00	0.59
**1,148.49**	7.05	86.20	0.10	0.32	0.00	0.58
**1,160.03**	7.06	86.92	0.10	0.32	0.00	0.58
**1,171.69**	7.07	87.65	0.10	0.32	0.00	0.58
**1,183.46**	7.08	88.39	0.10	0.32	0.00	0.58
**1,195.36**	7.09	89.13	0.10	0.32	0.00	0.58
**1,207.37**	7.10	89.87	0.10	0.32	0.00	0.57
**1,219.50**	7.11	90.62	0.10	0.32	0.00	0.57
**1,231.76**	7.12	91.38	0.11	0.32	0.00	0.57
**1,244.14**	7.13	92.14	0.11	0.32	0.00	0.57
**1,256.64**	7.14	92.91	0.11	0.32	0.00	0.56
**1,269.27**	7.15	93.68	0.11	0.33	0.00	0.56
**1,282.03**	7.16	94.46	0.11	0.33	0.00	0.56
**1,294.91**	7.17	95.24	0.11	0.33	0.00	0.56
**1,307.93**	7.18	96.03	0.11	0.33	0.00	0.56

Gross domestic product per capita (2015 PPP$)	Natural log of gross domestic product per capita (2015 PPP$)	Total health spending per capita (2015 PPP$)	Domestic government health spending per total health spending (proportion)	Out-of-pocket spending per total health spending (proportion)	Prepaid private spending per total health spending (proportion)	Development assistance for health per total health spending (proportion)
**1,321.07**	7.19	96.82	0.11	0.33	0.00	0.55
**1,334.35**	7.20	97.62	0.11	0.33	0.00	0.55
**1,347.76**	7.21	98.42	0.12	0.33	0.00	0.55
**1,361.31**	7.22	99.23	0.12	0.33	0.00	0.55
**1,374.99**	7.23	100.04	0.12	0.33	0.00	0.54
**1,388.81**	7.24	100.86	0.12	0.34	0.00	0.54
**1,402.76**	7.25	101.69	0.12	0.34	0.00	0.54
**1,416.86**	7.26	102.52	0.12	0.34	0.00	0.54
**1,431.10**	7.27	103.36	0.12	0.34	0.00	0.53
**1,445.48**	7.28	104.20	0.13	0.34	0.00	0.53
**1,460.01**	7.29	105.05	0.13	0.34	0.00	0.53
**1,474.68**	7.30	105.90	0.13	0.34	0.00	0.52
**1,489.51**	7.31	106.76	0.13	0.34	0.00	0.52
**1,504.48**	7.32	107.63	0.13	0.35	0.00	0.52
**1,519.60**	7.33	108.50	0.13	0.35	0.00	0.52
**1,534.87**	7.34	109.37	0.14	0.35	0.00	0.51
**1,550.29**	7.35	110.25	0.14	0.35	0.00	0.51
**1,565.87**	7.36	111.14	0.14	0.35	0.00	0.51
**1,581.61**	7.37	112.03	0.14	0.35	0.00	0.50
**1,597.51**	7.38	112.93	0.14	0.35	0.00	0.50
**1,613.56**	7.39	113.83	0.15	0.35	0.00	0.50
**1,629.78**	7.40	114.73	0.15	0.35	0.00	0.49
**1,646.16**	7.41	115.64	0.15	0.36	0.00	0.49
**1,662.70**	7.42	116.56	0.15	0.36	0.00	0.49
**1,679.41**	7.43	117.47	0.15	0.36	0.00	0.48
**1,696.29**	7.44	118.40	0.16	0.36	0.00	0.48
**1,713.34**	7.45	119.32	0.16	0.36	0.00	0.48
**1,730.56**	7.46	120.25	0.16	0.36	0.00	0.47
**1,747.95**	7.47	121.19	0.16	0.36	0.00	0.47
**1,765.52**	7.48	122.13	0.17	0.36	0.00	0.47
**1,783.26**	7.49	123.07	0.17	0.37	0.00	0.46
**1,801.18**	7.50	124.02	0.17	0.37	0.00	0.46
**1,819.29**	7.51	124.97	0.17	0.37	0.00	0.46
**1,837.57**	7.52	125.93	0.17	0.37	0.00	0.45
**1,856.04**	7.53	126.89	0.18	0.37	0.00	0.45

Gross domestic product per capita (2015 PPP$)	Natural log of gross domestic product per capita (2015 PPP$)	Total health spending per capita (2015 PPP$)	Domestic government health spending per total health spending (proportion)	Out-of-pocket spending per total health spending (proportion)	Prepaid private spending per total health spending (proportion)	Development assistance for health per total health spending (proportion)
**1,874.69**	7.54	127.86	0.18	0.37	0.00	0.44
**1,893.53**	7.55	128.83	0.18	0.37	0.00	0.44
**1,912.56**	7.56	129.81	0.18	0.37	0.00	0.44
**1,931.78**	7.57	130.79	0.19	0.37	0.00	0.43
**1,951.20**	7.58	131.77	0.19	0.38	0.00	0.43
**1,970.81**	7.59	132.76	0.19	0.38	0.00	0.43
**1,990.62**	7.60	133.76	0.19	0.38	0.00	0.42
**2,010.62**	7.61	134.76	0.20	0.38	0.01	0.42
**2,030.83**	7.62	135.77	0.20	0.38	0.01	0.41
**2,051.24**	7.63	136.79	0.20	0.38	0.01	0.41
**2,071.85**	7.64	137.81	0.21	0.38	0.01	0.41
**2,092.68**	7.65	138.84	0.21	0.38	0.01	0.40
**2,113.71**	7.66	139.87	0.21	0.38	0.01	0.40
**2,134.95**	7.67	140.92	0.21	0.39	0.01	0.40
**2,156.41**	7.68	141.97	0.22	0.39	0.01	0.39
**2,178.08**	7.69	143.02	0.22	0.39	0.01	0.39
**2,199.97**	7.70	144.09	0.22	0.39	0.01	0.38
**2,222.08**	7.71	145.16	0.22	0.39	0.01	0.38
**2,244.41**	7.72	146.25	0.23	0.39	0.01	0.38
**2,266.97**	7.73	147.34	0.23	0.39	0.01	0.37
**2,289.75**	7.74	148.44	0.23	0.39	0.01	0.37
**2,312.77**	7.75	149.55	0.24	0.39	0.01	0.36
**2,336.01**	7.76	150.67	0.24	0.39	0.01	0.36
**2,359.49**	7.77	151.81	0.24	0.39	0.01	0.36
**2,383.20**	7.78	152.95	0.25	0.40	0.01	0.35
**2,407.15**	7.79	154.10	0.25	0.40	0.01	0.35
**2,431.34**	7.80	155.26	0.25	0.40	0.01	0.35
**2,455.78**	7.81	156.44	0.26	0.40	0.01	0.34
**2,480.46**	7.82	157.62	0.26	0.40	0.01	0.34
**2,505.39**	7.83	158.82	0.26	0.40	0.01	0.33
**2,530.57**	7.84	160.03	0.26	0.40	0.01	0.33
**2,556.00**	7.85	161.25	0.27	0.40	0.01	0.33
**2,581.69**	7.86	162.48	0.27	0.40	0.01	0.32
**2,607.64**	7.87	163.73	0.27	0.40	0.01	0.32
**2,633.84**	7.88	164.99	0.28	0.40	0.01	0.31

Gross domestic product per capita (2015 PPP$)	Natural log of gross domestic product per capita (2015 PPP$)	Total health spending per capita (2015 PPP$)	Domestic government health spending per total health spending (proportion)	Out-of-pocket spending per total health spending (proportion)	Prepaid private spending per total health spending (proportion)	Development assistance for health per total health spending (proportion)
**2,660.31**	7.89	166.27	0.28	0.40	0.01	0.31
**2,687.05**	7.90	167.55	0.28	0.40	0.01	0.31
**2,714.06**	7.91	168.86	0.29	0.40	0.01	0.30
**2,741.33**	7.92	170.17	0.29	0.40	0.01	0.30
**2,768.88**	7.93	171.50	0.29	0.41	0.01	0.29
**2,796.71**	7.94	172.85	0.30	0.41	0.01	0.29
**2,824.82**	7.95	174.21	0.30	0.41	0.01	0.29
**2,853.21**	7.96	175.58	0.30	0.41	0.01	0.28
**2,881.88**	7.97	176.97	0.31	0.41	0.01	0.28
**2,910.85**	7.98	178.38	0.31	0.41	0.01	0.27
**2,940.10**	7.99	179.80	0.31	0.41	0.01	0.27
**2,969.65**	8.00	181.24	0.32	0.41	0.01	0.27
**2,999.50**	8.01	182.69	0.32	0.41	0.01	0.26
**3,029.64**	8.02	184.17	0.32	0.41	0.01	0.26
**3,060.09**	8.03	185.65	0.33	0.41	0.01	0.26
**3,090.84**	8.04	187.16	0.33	0.41	0.01	0.25
**3,121.91**	8.05	188.68	0.33	0.41	0.01	0.25
**3,153.28**	8.06	190.22	0.34	0.41	0.01	0.24
**3,184.97**	8.07	191.78	0.34	0.41	0.01	0.24
**3,216.98**	8.08	193.35	0.34	0.41	0.01	0.24
**3,249.31**	8.09	194.94	0.35	0.41	0.01	0.23
**3,281.97**	8.10	196.55	0.35	0.41	0.01	0.23
**3,314.96**	8.11	198.17	0.35	0.41	0.01	0.23
**3,348.27**	8.12	199.81	0.36	0.41	0.01	0.22
**3,381.92**	8.13	201.47	0.36	0.41	0.01	0.22
**3,415.91**	8.14	203.15	0.36	0.41	0.01	0.22
**3,450.24**	8.15	204.84	0.37	0.41	0.01	0.21
**3,484.92**	8.16	206.55	0.37	0.41	0.01	0.21
**3,519.94**	8.17	208.28	0.37	0.41	0.01	0.21
**3,555.32**	8.18	210.03	0.38	0.41	0.01	0.20
**3,591.05**	8.19	211.79	0.38	0.41	0.01	0.20
**3,627.14**	8.20	213.58	0.38	0.41	0.01	0.20
**3,663.59**	8.21	215.39	0.39	0.41	0.01	0.19
**3,700.41**	8.22	217.21	0.39	0.41	0.01	0.19
**3,737.60**	8.23	219.06	0.39	0.41	0.01	0.19

Gross domestic product per capita (2015 PPP$)	Natural log of gross domestic product per capita (2015 PPP$)	Total health spending per capita (2015 PPP$)	Domestic government health spending per total health spending (proportion)	Out-of-pocket spending per total health spending (proportion)	Prepaid private spending per total health spending (proportion)	Development assistance for health per total health spending (proportion)
**3,775.16**	8.24	220.92	0.40	0.41	0.01	0.18
**3,813.11**	8.25	222.81	0.40	0.41	0.01	0.18
**3,851.43**	8.26	224.71	0.40	0.41	0.01	0.18
**3,890.14**	8.27	226.64	0.40	0.41	0.01	0.18
**3,929.23**	8.28	228.59	0.41	0.41	0.01	0.17
**3,968.72**	8.29	230.56	0.41	0.41	0.01	0.17
**4,008.61**	8.30	232.56	0.41	0.41	0.01	0.17
**4,048.90**	8.31	234.57	0.42	0.41	0.01	0.16
**4,089.59**	8.32	236.61	0.42	0.41	0.01	0.16
**4,130.69**	8.33	238.67	0.42	0.41	0.01	0.16
**4,172.20**	8.34	240.76	0.42	0.41	0.01	0.16
**4,214.13**	8.35	242.86	0.43	0.41	0.01	0.15
**4,256.49**	8.36	244.99	0.43	0.41	0.01	0.15
**4,299.26**	8.37	247.15	0.43	0.41	0.01	0.15
**4,342.47**	8.38	249.33	0.44	0.41	0.01	0.15
**4,386.12**	8.39	251.54	0.44	0.41	0.01	0.14
**4,430.20**	8.40	253.77	0.44	0.41	0.01	0.14
**4,474.72**	8.41	256.02	0.44	0.41	0.01	0.14
**4,519.69**	8.42	258.31	0.45	0.41	0.01	0.13
**4,565.12**	8.43	260.62	0.45	0.41	0.01	0.13
**4,611.00**	8.44	262.95	0.45	0.41	0.01	0.13
**4,657.34**	8.45	265.32	0.45	0.41	0.01	0.13
**4,704.15**	8.46	267.71	0.46	0.41	0.01	0.12
**4,751.42**	8.47	270.13	0.46	0.41	0.01	0.12
**4,799.17**	8.48	272.58	0.46	0.41	0.01	0.12
**4,847.41**	8.49	275.05	0.47	0.41	0.01	0.12
**4,896.13**	8.50	277.56	0.47	0.41	0.01	0.12
**4,945.33**	8.51	280.10	0.47	0.41	0.01	0.11
**4,995.03**	8.52	282.67	0.47	0.41	0.01	0.11
**5,045.23**	8.53	285.27	0.48	0.40	0.01	0.11
**5,095.94**	8.54	287.91	0.48	0.40	0.01	0.11
**5,147.15**	8.55	290.58	0.48	0.40	0.01	0.10
**5,198.88**	8.56	293.28	0.48	0.40	0.01	0.10
**5,251.13**	8.57	296.02	0.49	0.40	0.01	0.10
**5,303.91**	8.58	298.80	0.49	0.40	0.01	0.10

Gross domestic product per capita (2015 PPP$)	Natural log of gross domestic product per capita (2015 PPP$)	Total health spending per capita (2015 PPP$)	Domestic government health spending per total health spending (proportion)	Out-of-pocket spending per total health spending (proportion)	Prepaid private spending per total health spending (proportion)	Development assistance for health per total health spending (proportion)
**5,357.21**	8.59	301.61	0.49	0.40	0.01	0.10
**5,411.05**	8.60	304.46	0.49	0.40	0.01	0.09
**5,465.44**	8.61	307.35	0.50	0.40	0.01	0.09
**5,520.37**	8.62	310.27	0.50	0.40	0.01	0.09
**5,575.85**	8.63	313.23	0.50	0.40	0.01	0.09
**5,631.88**	8.64	316.24	0.50	0.40	0.01	0.09
**5,688.49**	8.65	319.28	0.51	0.40	0.01	0.08
**5,745.66**	8.66	322.36	0.51	0.40	0.01	0.08
**5,803.40**	8.67	325.48	0.51	0.40	0.01	0.08
**5,861.73**	8.68	328.65	0.51	0.40	0.01	0.08
**5,920.64**	8.69	331.86	0.51	0.40	0.01	0.08
**5,980.14**	8.70	335.11	0.52	0.40	0.01	0.08
**6,040.24**	8.71	338.41	0.52	0.39	0.01	0.07
**6,100.95**	8.72	341.75	0.52	0.39	0.01	0.07
**6,162.26**	8.73	345.14	0.52	0.39	0.01	0.07
**6,224.19**	8.74	348.57	0.53	0.39	0.01	0.07
**6,286.75**	8.75	352.05	0.53	0.39	0.01	0.07
**6,349.93**	8.76	355.57	0.53	0.39	0.01	0.07
**6,413.75**	8.77	359.14	0.53	0.39	0.01	0.06
**6,478.21**	8.78	362.76	0.53	0.39	0.01	0.06
**6,543.32**	8.79	366.43	0.54	0.39	0.01	0.06
**6,609.08**	8.80	370.14	0.54	0.39	0.01	0.06
**6,675.50**	8.81	373.90	0.54	0.39	0.02	0.06
**6,742.59**	8.82	377.71	0.54	0.39	0.02	0.06
**6,810.35**	8.83	381.57	0.54	0.39	0.02	0.05
**6,878.80**	8.84	385.47	0.55	0.38	0.02	0.05
**6,947.93**	8.85	389.43	0.55	0.38	0.02	0.05
**7,017.76**	8.86	393.44	0.55	0.38	0.02	0.05
**7,088.29**	8.87	397.49	0.55	0.38	0.02	0.05
**7,159.53**	8.88	401.60	0.55	0.38	0.02	0.05
**7,231.48**	8.89	405.75	0.56	0.38	0.02	0.05
**7,304.16**	8.90	409.96	0.56	0.38	0.02	0.05
**7,377.57**	8.91	414.21	0.56	0.38	0.02	0.04
**7,451.71**	8.92	418.52	0.56	0.38	0.02	0.04
**7,526.60**	8.93	422.87	0.56	0.38	0.02	0.04

Gross domestic product per capita (2015 PPP$)	Natural log of gross domestic product per capita (2015 PPP$)	Total health spending per capita (2015 PPP$)	Domestic government health spending per total health spending (proportion)	Out-of-pocket spending per total health spending (proportion)	Prepaid private spending per total health spending (proportion)	Development assistance for health per total health spending (proportion)
**7,602.25**	8.94	427.28	0.56	0.38	0.02	0.04
**7,678.65**	8.95	431.74	0.57	0.38	0.02	0.04
**7,755.82**	8.96	436.25	0.57	0.37	0.02	0.04
**7,833.77**	8.97	440.81	0.57	0.37	0.02	0.04
**7,912.50**	8.98	445.42	0.57	0.37	0.02	0.04
**7,992.02**	8.99	450.09	0.57	0.37	0.02	0.04
**8,072.34**	9.00	454.82	0.58	0.37	0.02	0.03
**8,153.47**	9.01	459.59	0.58	0.37	0.02	0.03
**8,235.42**	9.02	464.43	0.58	0.37	0.02	0.03
**8,318.18**	9.03	469.33	0.58	0.37	0.02	0.03
**8,401.78**	9.04	474.28	0.58	0.37	0.02	0.03
**8,486.22**	9.05	479.29	0.58	0.37	0.02	0.03
**8,571.51**	9.06	484.37	0.59	0.37	0.02	0.03
**8,657.66**	9.07	489.51	0.59	0.36	0.02	0.03
**8,744.67**	9.08	494.72	0.59	0.36	0.02	0.03
**8,832.55**	9.09	499.99	0.59	0.36	0.02	0.03
**8,921.32**	9.10	505.34	0.59	0.36	0.02	0.03
**9,010.98**	9.11	510.75	0.59	0.36	0.02	0.02
**9,101.54**	9.12	516.23	0.59	0.36	0.02	0.02
**9,193.02**	9.13	521.79	0.60	0.36	0.02	0.02
**9,285.41**	9.14	527.42	0.60	0.36	0.02	0.02
**9,378.73**	9.15	533.13	0.60	0.36	0.02	0.02
**9,472.98**	9.16	538.92	0.60	0.36	0.02	0.02
**9,568.19**	9.17	544.80	0.60	0.36	0.02	0.02
**9,664.35**	9.18	550.75	0.60	0.36	0.02	0.02
**9,761.48**	9.19	556.80	0.60	0.35	0.02	0.02
**9,859.58**	9.20	562.93	0.61	0.35	0.02	0.02
**9,958.67**	9.21	569.15	0.61	0.35	0.02	0.02
**10,058.76**	9.22	575.47	0.61	0.35	0.02	0.02
**10,159.85**	9.23	581.87	0.61	0.35	0.02	0.02
**10,261.96**	9.24	588.36	0.61	0.35	0.02	0.02
**10,365.09**	9.25	594.95	0.61	0.35	0.02	0.01
**10,469.27**	9.26	601.64	0.61	0.35	0.02	0.01
**10,574.48**	9.27	608.42	0.61	0.35	0.02	0.01
**10,680.76**	9.28	615.30	0.62	0.35	0.02	0.01

Gross domestic product per capita (2015 PPP$)	Natural log of gross domestic product per capita (2015 PPP$)	Total health spending per capita (2015 PPP$)	Domestic government health spending per total health spending (proportion)	Out-of-pocket spending per total health spending (proportion)	Prepaid private spending per total health spending (proportion)	Development assistance for health per total health spending (proportion)
**10,788.10**	9.29	622.27	0.62	0.35	0.02	0.01
**10,896.53**	9.30	629.34	0.62	0.35	0.02	0.01
**11,006.04**	9.31	636.51	0.62	0.34	0.02	0.01
**11,116.65**	9.32	643.79	0.62	0.34	0.03	0.01
**11,228.37**	9.33	651.16	0.62	0.34	0.03	0.01
**11,341.22**	9.34	658.64	0.62	0.34	0.03	0.01
**11,455.20**	9.35	666.23	0.62	0.34	0.03	0.01
**11,570.33**	9.36	673.92	0.62	0.34	0.03	0.01
**11,686.61**	9.37	681.72	0.63	0.34	0.03	0.01
**11,804.06**	9.38	689.64	0.63	0.34	0.03	0.01
**11,922.70**	9.39	697.66	0.63	0.34	0.03	0.01
**12,042.52**	9.40	705.80	0.63	0.34	0.03	0.01
**12,163.55**	9.41	714.06	0.63	0.34	0.03	0.01
**12,285.80**	9.42	722.43	0.63	0.34	0.03	0.01
**12,409.27**	9.43	730.91	0.63	0.33	0.03	0.01
**12,533.99**	9.44	739.52	0.63	0.33	0.03	0.01
**12,659.96**	9.45	748.24	0.63	0.33	0.03	0.01
**12,787.19**	9.46	757.08	0.63	0.33	0.03	0.01
**12,915.70**	9.47	766.05	0.64	0.33	0.03	0.01
**13,045.51**	9.48	775.13	0.64	0.33	0.03	0.01
**13,176.62**	9.49	784.34	0.64	0.33	0.03	0.01
**13,309.05**	9.50	793.68	0.64	0.33	0.03	0.01
**13,442.80**	9.51	803.15	0.64	0.33	0.03	0.01
**13,577.91**	9.52	812.74	0.64	0.33	0.03	0.00
**13,714.37**	9.53	822.47	0.64	0.33	0.03	0.00
**13,852.20**	9.54	832.33	0.64	0.32	0.03	0.00
**13,991.42**	9.55	842.32	0.64	0.32	0.03	0.00
**14,132.03**	9.56	852.45	0.64	0.32	0.03	0.00
**14,274.06**	9.57	862.72	0.65	0.32	0.03	0.00
**14,417.52**	9.58	873.12	0.65	0.32	0.03	0.00
**14,562.42**	9.59	883.66	0.65	0.32	0.03	0.00
**14,708.77**	9.60	894.34	0.65	0.32	0.03	0.00
**14,856.60**	9.61	905.16	0.65	0.32	0.03	0.00
**15,005.91**	9.62	916.13	0.65	0.32	0.03	0.00
**15,156.72**	9.63	927.25	0.65	0.32	0.03	0.00

Gross domestic product per capita (2015 PPP$)	Natural log of gross domestic product per capita (2015 PPP$)	Total health spending per capita (2015 PPP$)	Domestic government health spending per total health spending (proportion)	Out-of-pocket spending per total health spending (proportion)	Prepaid private spending per total health spending (proportion)	Development assistance for health per total health spending (proportion)
**15,309.05**	9.64	938.53	0.65	0.32	0.03	0.00
**15,462.91**	9.65	949.96	0.65	0.31	0.03	0.00
**15,618.31**	9.66	961.55	0.65	0.31	0.03	0.00
**15,775.28**	9.67	973.30	0.66	0.31	0.03	0.00
**15,933.82**	9.68	985.22	0.66	0.31	0.03	0.00
**16,093.96**	9.69	997.30	0.66	0.31	0.03	0.00
**16,255.71**	9.70	1,009.55	0.66	0.31	0.03	0.00
**16,419.08**	9.71	1,021.97	0.66	0.31	0.03	0.00
**16,584.09**	9.72	1,034.56	0.66	0.31	0.03	0.00
**16,750.77**	9.73	1,047.32	0.66	0.31	0.03	0.00
**16,919.11**	9.74	1,060.26	0.66	0.31	0.03	0.00
**17,089.15**	9.75	1,073.37	0.66	0.30	0.03	0.00
**17,260.90**	9.76	1,086.66	0.67	0.30	0.03	0.00
**17,434.38**	9.77	1,100.13	0.67	0.30	0.03	0.00
**17,609.60**	9.78	1,113.77	0.67	0.30	0.03	0.00
**17,786.57**	9.79	1,127.60	0.67	0.30	0.03	0.00
**17,965.33**	9.80	1,141.60	0.67	0.30	0.03	0.00
**18,145.89**	9.81	1,155.79	0.67	0.30	0.03	0.00
**18,328.26**	9.82	1,170.16	0.67	0.30	0.03	0.00
**18,512.46**	9.83	1,184.72	0.67	0.30	0.03	0.00
**18,698.51**	9.84	1,199.46	0.67	0.29	0.03	0.00
**18,886.44**	9.85	1,214.38	0.68	0.29	0.03	0.00
**19,076.25**	9.86	1,229.49	0.68	0.29	0.03	0.00
**19,267.97**	9.87	1,244.79	0.68	0.29	0.03	0.00
**19,461.61**	9.88	1,260.26	0.68	0.29	0.03	0.00
**19,657.21**	9.89	1,275.93	0.68	0.29	0.03	0.00
**19,854.76**	9.90	1,291.79	0.68	0.29	0.03	0.00
**20,054.31**	9.91	1,307.83	0.68	0.29	0.03	0.00
**20,255.86**	9.92	1,324.07	0.68	0.28	0.03	0.00
**20,459.43**	9.93	1,340.50	0.68	0.28	0.03	0.00
**20,665.05**	9.94	1,357.13	0.69	0.28	0.03	0.00
**20,872.74**	9.95	1,373.96	0.69	0.28	0.03	0.00
**21,082.51**	9.96	1,390.99	0.69	0.28	0.03	0.00
**21,294.40**	9.97	1,408.22	0.69	0.28	0.03	0.00
**21,508.41**	9.98	1,425.64	0.69	0.28	0.03	0.00

Gross domestic product per capita (2015 PPP$)	Natural log of gross domestic product per capita (2015 PPP$)	Total health spending per capita (2015 PPP$)	Domestic government health spending per total health spending (proportion)	Out-of-pocket spending per total health spending (proportion)	Prepaid private spending per total health spending (proportion)	Development assistance for health per total health spending (proportion)
**21,724.57**	9.99	1,443.27	0.69	0.27	0.03	0.00
**21,942.91**	10.00	1,461.11	0.69	0.27	0.03	0.00
**22,163.44**	10.01	1,479.15	0.70	0.27	0.03	0.00
**22,386.18**	10.02	1,497.41	0.70	0.27	0.03	0.00
**22,611.17**	10.03	1,515.89	0.70	0.27	0.03	0.00
**22,838.41**	10.04	1,534.60	0.70	0.27	0.03	0.00
**23,067.94**	10.05	1,553.53	0.70	0.27	0.03	0.00
**23,299.78**	10.06	1,572.70	0.70	0.27	0.03	0.00
**23,533.95**	10.07	1,592.09	0.70	0.26	0.03	0.00
**23,770.47**	10.08	1,611.72	0.70	0.26	0.03	0.00
**24,009.36**	10.09	1,631.58	0.71	0.26	0.03	0.00
**24,250.66**	10.10	1,651.68	0.71	0.26	0.03	0.00
**24,494.38**	10.11	1,672.00	0.71	0.26	0.03	0.00
**24,740.56**	10.12	1,692.55	0.71	0.26	0.03	0.00
**24,989.21**	10.13	1,713.32	0.71	0.26	0.03	0.00
**25,240.35**	10.14	1,734.32	0.71	0.25	0.03	0.00
**25,494.02**	10.15	1,755.53	0.71	0.25	0.03	0.00
**25,750.24**	10.16	1,776.97	0.72	0.25	0.03	0.00
**26,009.03**	10.17	1,798.63	0.72	0.25	0.03	0.00
**26,270.43**	10.18	1,820.52	0.72	0.25	0.03	0.00
**26,534.45**	10.19	1,842.63	0.72	0.25	0.03	0.00
**26,801.13**	10.20	1,864.97	0.72	0.25	0.03	0.00
**27,070.48**	10.21	1,887.53	0.72	0.25	0.03	0.00
**27,342.54**	10.22	1,910.32	0.72	0.24	0.03	0.00
**27,617.34**	10.23	1,933.32	0.72	0.24	0.03	0.00
**27,894.90**	10.24	1,956.54	0.73	0.24	0.03	0.00
**28,175.25**	10.25	1,979.96	0.73	0.24	0.03	0.00
**28,458.42**	10.26	2,003.59	0.73	0.24	0.03	0.00
**28,744.43**	10.27	2,027.42	0.73	0.24	0.03	0.00
**29,033.31**	10.28	2,051.45	0.73	0.24	0.03	0.00
**29,325.10**	10.29	2,075.66	0.73	0.23	0.03	0.00
**29,619.83**	10.30	2,100.06	0.73	0.23	0.03	0.00
**29,917.51**	10.31	2,124.62	0.73	0.23	0.03	0.00
**30,218.19**	10.32	2,149.36	0.74	0.23	0.03	0.00
**30,521.88**	10.33	2,174.24	0.74	0.23	0.03	0.00

Gross domestic product per capita (2015 PPP$)	Natural log of gross domestic product per capita (2015 PPP$)	Total health spending per capita (2015 PPP$)	Domestic government health spending per total health spending (proportion)	Out-of-pocket spending per total health spending (proportion)	Prepaid private spending per total health spending (proportion)	Development assistance for health per total health spending (proportion)
**30,828.63**	10.34	2,199.28	0.74	0.23	0.03	0.00
**31,138.47**	10.35	2,224.46	0.74	0.23	0.03	0.00
**31,451.41**	10.36	2,249.78	0.74	0.23	0.03	0.00
**31,767.51**	10.37	2,275.22	0.74	0.22	0.03	0.00
**32,086.77**	10.38	2,300.77	0.74	0.22	0.03	0.00
**32,409.25**	10.39	2,326.42	0.74	0.22	0.03	0.00
**32,734.97**	10.40	2,352.17	0.75	0.22	0.03	0.00
**33,063.96**	10.41	2,378.00	0.75	0.22	0.03	0.00
**33,396.26**	10.42	2,403.90	0.75	0.22	0.03	0.00
**33,731.90**	10.43	2,429.86	0.75	0.22	0.03	0.00
**34,070.91**	10.44	2,455.87	0.75	0.22	0.03	0.00
**34,413.33**	10.45	2,481.92	0.75	0.21	0.03	0.00
**34,759.19**	10.46	2,507.99	0.75	0.21	0.03	0.00
**35,108.52**	10.47	2,534.08	0.75	0.21	0.03	0.00
**35,461.37**	10.48	2,560.18	0.75	0.21	0.03	0.00
**35,817.76**	10.49	2,586.28	0.76	0.21	0.03	0.00
**36,177.74**	10.50	2,612.37	0.76	0.21	0.03	0.00
**36,541.33**	10.51	2,638.44	0.76	0.21	0.03	0.00
**36,908.57**	10.52	2,664.47	0.76	0.21	0.03	0.00
**37,279.51**	10.53	2,690.48	0.76	0.21	0.03	0.00
**37,654.18**	10.54	2,716.45	0.76	0.20	0.03	0.00
**38,032.61**	10.55	2,742.39	0.76	0.20	0.03	0.00
**38,414.84**	10.56	2,768.30	0.76	0.20	0.03	0.00
**38,800.92**	10.57	2,794.15	0.76	0.20	0.03	0.00
**39,190.88**	10.58	2,819.94	0.77	0.20	0.03	0.00
**39,584.75**	10.59	2,845.68	0.77	0.20	0.03	0.00
**39,982.58**	10.60	2,871.35	0.77	0.20	0.03	0.00
**40,384.41**	10.61	2,896.95	0.77	0.20	0.03	0.00
**40,790.29**	10.62	2,922.48	0.77	0.20	0.03	0.00
**41,200.23**	10.63	2,947.92	0.77	0.19	0.03	0.00
**41,614.30**	10.64	2,973.27	0.77	0.19	0.04	0.00
**42,032.54**	10.65	2,998.53	0.77	0.19	0.04	0.00
**42,454.97**	10.66	3,023.70	0.77	0.19	0.04	0.00
**42,881.65**	10.67	3,048.78	0.77	0.19	0.04	0.00
**43,312.61**	10.68	3,073.77	0.77	0.19	0.04	0.00

Gross domestic product per capita (2015 PPP$)	Natural log of gross domestic product per capita (2015 PPP$)	Total health spending per capita (2015 PPP$)	Domestic government health spending per total health spending (proportion)	Out-of-pocket spending per total health spending (proportion)	Prepaid private spending per total health spending (proportion)	Development assistance for health per total health spending (proportion)
**43,747.91**	10.69	3,098.68	0.78	0.19	0.04	0.00
**44,187.59**	10.70	3,123.49	0.78	0.19	0.04	0.00
**44,631.68**	10.71	3,148.20	0.78	0.19	0.04	0.00
**45,080.23**	10.72	3,172.81	0.78	0.19	0.04	0.00
**45,533.30**	10.73	3,197.29	0.78	0.18	0.04	0.00
**45,990.92**	10.74	3,221.66	0.78	0.18	0.04	0.00
**46,453.13**	10.75	3,245.90	0.78	0.18	0.04	0.00
**46,920.00**	10.76	3,270.00	0.78	0.18	0.04	0.00
**47,391.55**	10.77	3,293.96	0.78	0.18	0.04	0.00
**47,867.84**	10.78	3,317.77	0.78	0.18	0.04	0.00
**48,348.92**	10.79	3,341.41	0.78	0.18	0.04	0.00
**48,834.84**	10.80	3,364.89	0.78	0.18	0.04	0.00
**49,325.64**	10.81	3,388.20	0.79	0.18	0.04	0.00
**49,821.37**	10.82	3,411.32	0.79	0.18	0.04	0.00
**50,322.08**	10.83	3,434.25	0.79	0.18	0.04	0.00
**50,827.82**	10.84	3,456.98	0.79	0.17	0.04	0.00
**51,338.65**	10.85	3,479.51	0.79	0.17	0.04	0.00
**51,854.61**	10.86	3,501.81	0.79	0.17	0.04	0.00
**52,375.76**	10.87	3,523.90	0.79	0.17	0.04	0.00
**52,902.15**	10.88	3,545.75	0.79	0.17	0.04	0.00
**53,433.82**	10.89	3,567.36	0.79	0.17	0.04	0.00
**53,970.84**	10.90	3,588.73	0.79	0.17	0.04	0.00
**54,513.26**	10.91	3,609.84	0.79	0.17	0.04	0.00
**55,061.13**	10.92	3,630.68	0.79	0.17	0.04	0.00
**55,614.50**	10.93	3,651.25	0.79	0.17	0.04	0.00
**56,173.43**	10.94	3,671.54	0.79	0.17	0.04	0.00
**56,737.98**	10.95	3,691.54	0.80	0.17	0.04	0.00
**57,308.21**	10.96	3,711.24	0.80	0.16	0.04	0.00
**57,884.17**	10.97	3,730.64	0.80	0.16	0.04	0.00
**58,465.91**	10.98	3,749.72	0.80	0.16	0.04	0.00
**59,053.51**	10.99	3,768.48	0.80	0.16	0.04	0.00
**59,647.00**	11.00	3,786.91	0.80	0.16	0.04	0.00
**60,246.47**	11.01	3,805.00	0.80	0.16	0.04	0.00
**60,851.95**	11.02	3,822.75	0.80	0.16	0.04	0.00
**61,463.53**	11.03	3,840.14	0.80	0.16	0.04	0.00

Gross domestic product per capita (2015 PPP$)	Natural log of gross domestic product per capita (2015 PPP$)	Total health spending per capita (2015 PPP$)	Domestic government health spending per total health spending (proportion)	Out-of-pocket spending per total health spending (proportion)	Prepaid private spending per total health spending (proportion)	Development assistance for health per total health spending (proportion)
**62,081.25**	11.04	3,857.17	0.80	0.16	0.04	0.00
**62,705.17**	11.05	3,873.83	0.80	0.16	0.04	0.00
**63,335.37**	11.06	3,890.11	0.80	0.16	0.04	0.00
**63,971.90**	11.07	3,906.01	0.80	0.16	0.04	0.00
**64,614.83**	11.08	3,921.51	0.80	0.16	0.04	0.00
**65,264.22**	11.09	3,936.62	0.80	0.16	0.04	0.00
**65,920.13**	11.10	3,951.31	0.80	0.15	0.04	0.00
**66,582.64**	11.11	3,965.59	0.80	0.15	0.04	0.00
**67,251.81**	11.12	3,979.44	0.80	0.15	0.04	0.00
**67,927.70**	11.13	3,992.87	0.81	0.15	0.04	0.00
**68,610.38**	11.14	4,005.86	0.81	0.15	0.04	0.00
**69,299.93**	11.15	4,018.40	0.81	0.15	0.04	0.00
**69,996.41**	11.16	4,030.49	0.81	0.15	0.04	0.00
**70,699.88**	11.17	4,042.13	0.81	0.15	0.04	0.00
**71,410.43**	11.18	4,053.30	0.81	0.15	0.04	0.00
**72,128.12**	11.19	4,064.00	0.81	0.15	0.04	0.00
**72,853.02**	11.20	4,074.22	0.81	0.15	0.04	0.00
**73,585.20**	11.21	4,083.96	0.81	0.15	0.04	0.00
**74,324.74**	11.22	4,093.20	0.81	0.15	0.04	0.00
**75,071.72**	11.23	4,101.96	0.81	0.15	0.04	0.00
**75,826.20**	11.24	4,110.21	0.81	0.15	0.04	0.00
**76,588.27**	11.25	4,117.95	0.81	0.15	0.04	0.00
**77,357.99**	11.26	4,125.18	0.81	0.15	0.04	0.00
**78,135.45**	11.27	4,131.90	0.81	0.15	0.04	0.00
**78,920.73**	11.28	4,138.09	0.81	0.14	0.04	0.00
**79,713.90**	11.29	4,143.75	0.81	0.14	0.04	0.00
**80,515.03**	11.30	4,148.88	0.81	0.14	0.05	0.00
**81,324.23**	11.31	4,153.47	0.81	0.14	0.05	0.00
**82,141.55**	11.32	4,157.53	0.81	0.14	0.05	0.00
**82,967.09**	11.33	4,147.18	0.81	0.14	0.05	0.00
**83,800.91**	11.34	4,149.96	0.81	0.14	0.05	0.00
**84,643.13**	11.35	4,152.19	0.81	0.14	0.05	0.00
**85,493.80**	11.36	4,153.86	0.81	0.14	0.05	0.00
**86,353.03**	11.37	4,154.98	0.81	0.14	0.05	0.00
**87,220.90**	11.38	4,155.53	0.81	0.14	0.05	0.00

Gross domestic product per capita (2015 PPP$)	Natural log of gross domestic product per capita (2015 PPP$)	Total health spending per capita (2015 PPP$)	Domestic government health spending per total health spending (proportion)	Out-of-pocket spending per total health spending (proportion)	Prepaid private spending per total health spending (proportion)	Development assistance for health per total health spending (proportion)
**88,097.48**	11.39	4,155.52	0.81	0.14	0.05	0.00
**88,982.88**	11.40	4,154.95	0.81	0.14	0.05	0.00
**89,877.16**	11.41	4,153.80	0.81	0.14	0.05	0.00
**90,780.45**	11.42	4,134.29	0.81	0.14	0.05	0.00
**91,692.80**	11.43	4,131.86	0.81	0.14	0.05	0.00
**92,614.34**	11.44	4,128.87	0.81	0.14	0.05	0.00
**93,545.13**	11.45	4,125.29	0.81	0.14	0.05	0.00
**94,485.27**	11.46	4,121.15	0.81	0.14	0.05	0.00
**95434.86**	11.47	4,116.42	0.81	0.14	0.05	0.00
**96,394.00**	11.48	4,111.12	0.81	0.14	0.05	0.00
**97,362.77**	11.49	4,105.25	0.81	0.14	0.05	0.00
**98,341.28**	11.50	4,098.79	0.81	0.14	0.05	0.00
**99,329.63**	11.51	4,091.76	0.81	0.14	0.05	0.00
**100,327.90**	11.52	4,084.15	0.81	0.14	0.05	0.00
**101,336.20**	11.53	4,075.97	0.81	0.13	0.05	0.00
**102,354.70**	11.54	4,067.22	0.81	0.13	0.05	0.00
**103,383.40**	11.55	3,972.47	0.81	0.13	0.06	0.00
**104,422.40**	11.56	3,961.45	0.81	0.13	0.06	0.00
**105,471.80**	11.57	3,949.88	0.81	0.13	0.06	0.00
**106,531.80**	11.58	3,937.75	0.81	0.13	0.06	0.00
**107,602.50**	11.59	3,925.06	0.81	0.13	0.06	0.00
**108,683.90**	11.60	3,911.83	0.81	0.13	0.06	0.00
**109,776.20**	11.61	3,898.04	0.81	0.13	0.06	0.00
**110,879.50**	11.62	3,883.71	0.81	0.13	0.06	0.00
**111,993.80**	11.63	3,868.84	0.81	0.13	0.06	0.00
**113,119.40**	11.64	3,853.43	0.81	0.13	0.06	0.00
**114,256.30**	11.65	3,837.49	0.81	0.13	0.06	0.00
**115,404.60**	11.66	3,821.02	0.81	0.13	0.06	0.00
**116,564.40**	11.67	3,571.78	0.82	0.12	0.06	0.00
**117,735.90**	11.68	3,551.98	0.82	0.12	0.06	0.00
**118,919.20**	11.69	3,531.72	0.82	0.12	0.06	0.00
**120,114.30**	11.70	3,511.00	0.82	0.12	0.06	0.00
**121,321.50**	11.71	3,489.84	0.82	0.12	0.06	0.00

Table 12. Estimates of health spending by Socio-demographic Index based on Multivariate Loess Estimation Method

Socio-demographic Index	Total health spending per capita (2015 purchasing-power adjusted $)	Domestic government health spending per total health spending (proportion)	Out-of-pocket spending per total health spending (proportion)	Prepaid private spending per total health spending (proportion)	Development assistance for health per total health spending (proportion)
**0.08**	57.38	0.06	0.38	0.00	0.56
**0.09**	58.45	0.06	0.38	0.00	0.56
**0.1**	60.31	0.07	0.38	0.00	0.56
**0.11**	61.35	0.07	0.37	0.00	0.56
**0.12**	62.83	0.08	0.37	0.00	0.55
**0.13**	64.17	0.08	0.36	0.00	0.56
**0.14**	65.52	0.08	0.36	0.00	0.56
**0.15**	67.04	0.08	0.36	0.00	0.56
**0.16**	68.64	0.08	0.35	0.00	0.56
**0.17**	70.34	0.09	0.35	0.00	0.56
**0.18**	72.14	0.09	0.35	0.00	0.56
**0.19**	74.05	0.09	0.35	0.00	0.55
**0.2**	76.07	0.10	0.35	0.00	0.55
**0.21**	78.21	0.10	0.35	0.00	0.55
**0.22**	80.47	0.11	0.35	0.00	0.54
**0.23**	82.86	0.11	0.35	0.00	0.54
**0.24**	85.40	0.12	0.35	0.00	0.53
**0.25**	88.08	0.12	0.36	0.00	0.52
**0.26**	90.92	0.13	0.36	0.00	0.51
**0.27**	93.93	0.13	0.36	0.00	0.50
**0.28**	97.12	0.14	0.36	0.00	0.49
**0.29**	100.50	0.15	0.37	0.00	0.48
**0.3**	104.08	0.16	0.37	0.00	0.47
**0.31**	107.88	0.16	0.38	0.00	0.46
**0.32**	111.90	0.17	0.38	0.00	0.44
**0.33**	116.17	0.18	0.39	0.00	0.43
**0.34**	120.71	0.19	0.39	0.00	0.41
**0.35**	125.52	0.20	0.40	0.00	0.40
**0.36**	130.63	0.21	0.40	0.00	0.38

**Socio-demographic Index**	Total health spending per capita (2015 purchasing-power adjusted $)	Domestic government health spending per total health spending (proportion)	Out-of-pocket spending per total health spending (proportion)	Prepaid private spending per total health spending (proportion)	Development assistance for health per total health spending (proportion)
**0.37**	136.05	0.23	0.41	0.00	0.36
**0.38**	141.82	0.24	0.41	0.01	0.35
**0.39**	147.96	0.25	0.42	0.01	0.33
**0.4**	154.49	0.26	0.42	0.01	0.31
**0.41**	161.43	0.28	0.42	0.01	0.29
**0.42**	168.83	0.29	0.43	0.01	0.28
**0.43**	176.71	0.30	0.43	0.01	0.26
**0.44**	185.11	0.32	0.43	0.01	0.24
**0.45**	194.07	0.33	0.44	0.01	0.22
**0.46**	203.63	0.35	0.44	0.01	0.20
**0.47**	213.84	0.36	0.44	0.01	0.19
**0.48**	224.75	0.38	0.44	0.01	0.17
**0.49**	236.40	0.39	0.44	0.01	0.16
**0.5**	248.86	0.41	0.44	0.01	0.14
**0.51**	262.20	0.42	0.44	0.01	0.13
**0.52**	276.48	0.44	0.44	0.01	0.11
**0.53**	291.77	0.45	0.43	0.01	0.10
**0.54**	308.17	0.47	0.43	0.01	0.09
**0.55**	325.76	0.48	0.43	0.01	0.08
**0.56**	344.63	0.49	0.42	0.01	0.07
**0.57**	364.90	0.51	0.42	0.02	0.06
**0.58**	386.68	0.52	0.41	0.02	0.05
**0.59**	410.11	0.53	0.40	0.02	0.05
**0.6**	435.31	0.54	0.40	0.02	0.04
**0.61**	462.44	0.56	0.39	0.02	0.04
**0.62**	491.67	0.57	0.38	0.02	0.03
**0.63**	523.18	0.58	0.38	0.02	0.03
**0.64**	557.17	0.59	0.37	0.02	0.02
**0.65**	593.87	0.60	0.36	0.02	0.02
**0.66**	633.50	0.61	0.35	0.02	0.02

**Socio-demographic Index**	Total health spending per capita (2015 purchasing-power adjusted $)	Domestic government health spending per total health spending (proportion)	Out-of-pocket spending per total health spending (proportion)	Prepaid private spending per total health spending (proportion)	Development assistance for health per total health spending (proportion)
**0.67**	676.35	0.62	0.35	0.02	0.01
**0.68**	722.69	0.63	0.34	0.02	0.01
**0.69**	772.85	0.64	0.33	0.02	0.01
**0.7**	827.19	0.64	0.32	0.02	0.01
**0.71**	886.07	0.65	0.32	0.02	0.01
**0.72**	949.95	0.66	0.31	0.02	0.01
**0.73**	1,019.28	0.67	0.30	0.03	0.00
**0.74**	1,094.58	0.68	0.29	0.03	0.00
**0.75**	1,176.42	0.68	0.29	0.03	0.00
**0.76**	1,265.44	0.69	0.28	0.03	0.00
**0.77**	1,362.34	0.70	0.27	0.03	0.00
**0.78**	1,467.88	0.70	0.26	0.03	0.00
**0.79**	1,582.93	0.71	0.26	0.03	0.00
**0.8**	1,708.42	0.72	0.25	0.03	0.00
**0.81**	1,845.41	0.72	0.24	0.03	0.00
**0.82**	1,995.05	0.73	0.24	0.03	0.00
**0.83**	2,158.64	0.74	0.23	0.03	0.00
**0.84**	2,337.60	0.74	0.23	0.03	0.00
**0.85**	2,533.52	0.75	0.22	0.03	0.00
**0.86**	2,748.18	0.75	0.21	0.03	0.00
**0.87**	2,983.53	0.76	0.21	0.04	0.00
**0.88**	3,241.76	0.76	0.20	0.04	0.00
**0.89**	3,525.31	0.77	0.20	0.04	0.00
**0.9**	3,836.89	0.77	0.19	0.04	0.00
**0.91**	4,179.53	0.78	0.19	0.04	0.00
**0.92**	4,556.60	0.78	0.18	0.04	0.00
**0.93**	4,976.82	0.78	0.17	0.04	0.00

Table 13. Modeled per capita spending by type of care, 2014

Gross domestic product per capita decile	Modeled per capita spending on inpatient care, curative and rehabilitative (2015 PPP $)	Modeled per capita spending on outpatient care, curative and rehabilitative (2015 PPP $)	Modeled per capita spending on long-term care (2015 PPP $)	Modeled per capita spending on ancillary services (2015 PPP $)	Modeled per capita spending on medical goods (2015 PPP $)	Modeled per capita spending on immunization & early disease detection (2015 PPP $)	Modeled per capita spending on governance, health system, and financing admin. (2015 PPP $)	Modeled per capita spending on other types of care (2015 PPP $)
0	56	62	3	7	44	10	12	4
1	71	77	5	9	57	11	13	5
2	96	102	8	14	78	14	16	6
3	105	111	11	16	85	14	15	6
4	120	126	17	19	95	15	16	6
5	150	159	30	24	113	19	18	7
6	163	175	47	27	114	21	19	7
7	307	334	128	49	194	39	34	14
8	457	506	265	72	256	61	49	22
9	1,069	1,212	839	165	523	148	116	55
10	1,019	1,183	1,046	153	429	149	112	58

Table 14. Classifications of countries by World Bank income groups

High-income	Upper-middle-income	Lower-middle-income	Low-income
**Andorra**	Albania	Armenia	Afghanistan
**Antigua and Barbuda**	Algeria	Bhutan	Bangladesh
**Australia**	Angola	Bolivia	Benin
**Austria**	Argentina	Cameroon	Burkina Faso
**Bahrain**	Azerbaijan	Cape Verde	Burundi
**Barbados**	Belarus	Congo	Cambodia
**Belgium**	Belize	Cote d'Ivoire	Central African Republic
**Brunei**	Bosnia and Herzegovina	Djibouti	Chad
**Canada**	Botswana	Egypt	Comoros
**Chile**	Brazil	El Salvador	Democratic Republic of the Congo
**Croatia**	Bulgaria	Federated States of Micronesia	Eritrea
**Cyprus**	China	Georgia	Ethiopia
**Czech Republic**	Colombia	Ghana	Guinea
**Denmark**	Costa Rica	Guatemala	Guinea-Bissau
**Equatorial Guinea**	Cuba	Guyana	Haiti
**Estonia**	Dominica	Honduras	Kenya
**Finland**	Dominican Republic	India	Kyrgyzstan
**France**	Ecuador	Indonesia	Liberia
**Germany**	Fiji	Kiribati	Madagascar
**Greece**	Gabon	Laos	Malawi
**Iceland**	Grenada	Lesotho	Mali
**Ireland**	Hungary	Mauritania	Mozambique
**Israel**	Iran	Moldova	Myanmar
**Italy**	Iraq	Mongolia	Nepal
**Japan**	Jamaica	Morocco	Niger
**Kuwait**	Jordan	Nicaragua	Rwanda
**Latvia**	Kazakhstan	Nigeria	Sierra Leone
**Lithuania**	Lebanon	Pakistan	Somalia
**Luxembourg**	Libya	Papua New Guinea	South Sudan
**Malta**	Macedonia	Paraguay	Tajikistan
**Netherlands**	Malaysia	Philippines	Tanzania
**New Zealand**	Maldives	Samoa	The Gambia
**Norway**	Marshall Islands	Sao Tome and Principe	Togo
**Oman**	Mauritius	Senegal	Uganda
**Poland**	Mexico	Solomon Islands	
**Portugal**	Montenegro	Sri Lanka	
**Qatar**	Namibia	Sudan	
**Russia**	Panama	Swaziland	

High-income	Upper-middle-income	Lower-middle-income	Low-income
**Saudi Arabia**	Peru	Syria	
**Singapore**	Romania	Timor-Leste	
**Slovakia**	Saint Lucia	Ukraine	
**Slovenia**	Vincent and the Grenadines	Uzbekistan	
**South Korea**	Serbia	Vanuatu	
**Spain**	Seychelles	Vietnam	
**Sweden**	South Africa	Yemen	
**Switzerland**	Suriname	Zambia	
**The Bahamas**	Thailand		
**Trinidad and Tobago**	Tonga		
**United Arab Emirates**	Tunisia		
**United Kingdom**	Turkey		
**United States**	Turkmenistan		
**Uruguay**	Venezuela		

Table 15. Classifications of countries by Global Burden of Disease geographical regions

High-income	Central Europe, Eastern Europe, and Central Asia	Sub-Saharan Africa	North Africa and Middle East	South Asia	Southeast Asia, East Asia, and Oceania	Latin America and Caribbean
**Andorra**	Albania	Angola	Afghanistan	Bangla desh	Cambodia	Antigua and Barbuda
**Argentina**	Armenia	Benin	Algeria	Bhutan	China	Barbados
**Australia**	Azerbaijan	Botswana	Bahrain	India	Federated States of Micronesia	Belize
**Austria**	Belarus	Burkina Faso	Egypt	Nepal	Fiji	Bolivia
**Belgium**	Bosnia and Herzegovina	Burundi	Iran	Pakista n	Indonesia	Brazil
**Brunei**	Bulgaria	Cameroon	Iraq		Kiribati	Colombia
**Canada**	Croatia	Cape Verde	Jordan		Laos	Costa Rica
**Chile**	Czech Republic	Central African Republic	Kuwait		Malaysia	Cuba
**Cyprus**	Estonia	Chad	Lebanon		Maldives	Dominica
**Denmark**	Georgia	Comoros	Libya		Marshall Islands	Dominican Republic
**Finland**	Hungary	Congo	Morocco		Mauritius	Ecuador
**France**	Kazakhstan	Cote d'Ivoire	Oman		Myanmar	El Salvador
**Germany**	Kyrgyzstan	Democratic Republic of the Congo	Qatar		Papua New Guinea	Grenada
**Greece**	Latvia	Djibouti	Saudi Arabia		Philippines	Guatemala
**Iceland**	Lithuania	Equatorial Guinea	Sudan		Samoa	Guyana
**Ireland**	Macedonia	Eritrea	Syria		Seychelles	Haiti

High-income	Central Europe, Eastern Europe, and Central Asia	Sub-Saharan Africa	North Africa and Middle East	South Asia	Southeast Asia, East Asia, and Oceania	Latin America and Caribbean
**Israel**	Moldova	Ethiopia	Tunisia		Solomon Islands	Honduras
**Italy**	Mongolia	Gabon	Turkey		Sri Lanka	Jamaica
**Japan**	Montenegro	Ghana	United Arab Emirates		Thailand	Mexico
**Luxembourg**	Poland	Guinea	Yemen		Timor-Leste	Nicaragua
**Malta**	Romania	Guinea-Bissau			Tonga	Panama
**Netherlands**	Russia	Kenya			Vanuatu	Paraguay
**New Zealand**	Serbia	Lesotho			Vietnam	Peru
**Norway**	Slovakia	Liberia				Saint Lucia
**Portugal**	Slovenia	Madagascar				Saint Vincent and the Grenadines
**Singapore**	Tajikistan	Malawi				Suriname
**South Korea**	Turkmenistan	Mali				The Bahamas
**Spain**	Ukraine	Mauritania				Trinidad and Tobago
**Sweden**	Uzbekistan	Mozambique				Venezuela
**Switzerland**		Namibia				
**United Kingdom**		Niger				
**United States**		Nigeria				
**Uruguay**		Rwanda				
		Sao Tome and Principe				
		Senegal				
		Sierra Leone				
		Somalia				
		South Africa				
		South Sudan				
		Swaziland				
		Tanzania				
		The Gambia				
		Togo				
		Uganda				
		Zambia				

Figure 18. Comparison of total health spending estimates from the IHME database to total health estimates from NHAs

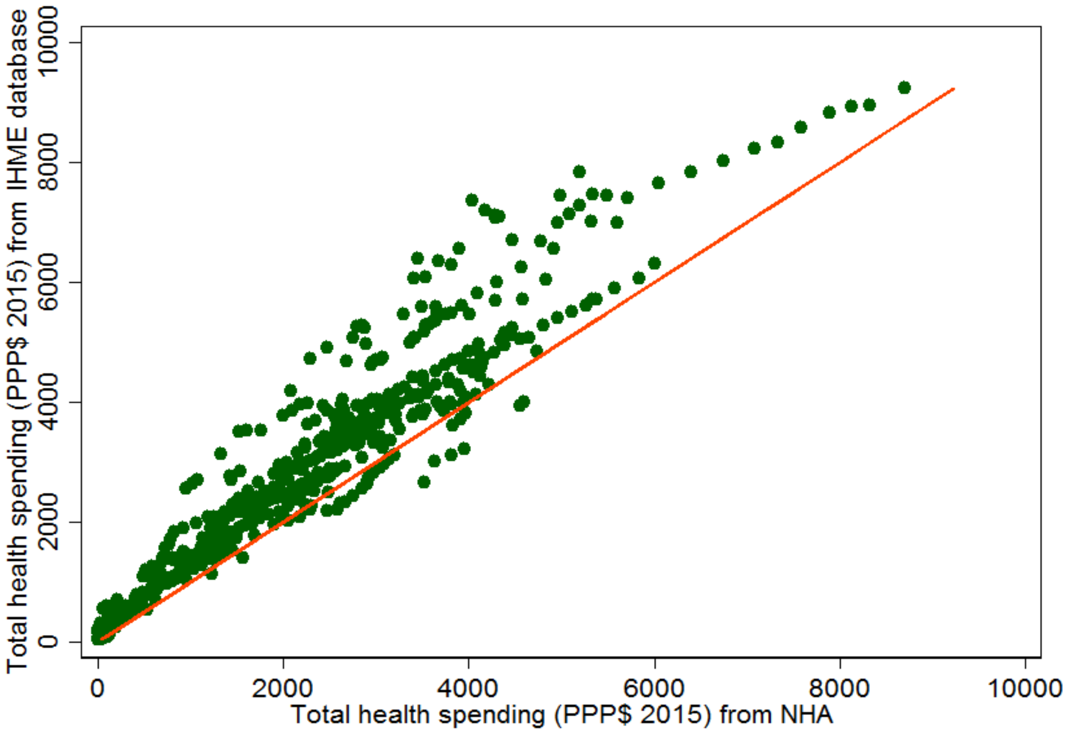

Figure 19. Comparison of government health spending estimates from the IHME database to government health estimates from NHAs

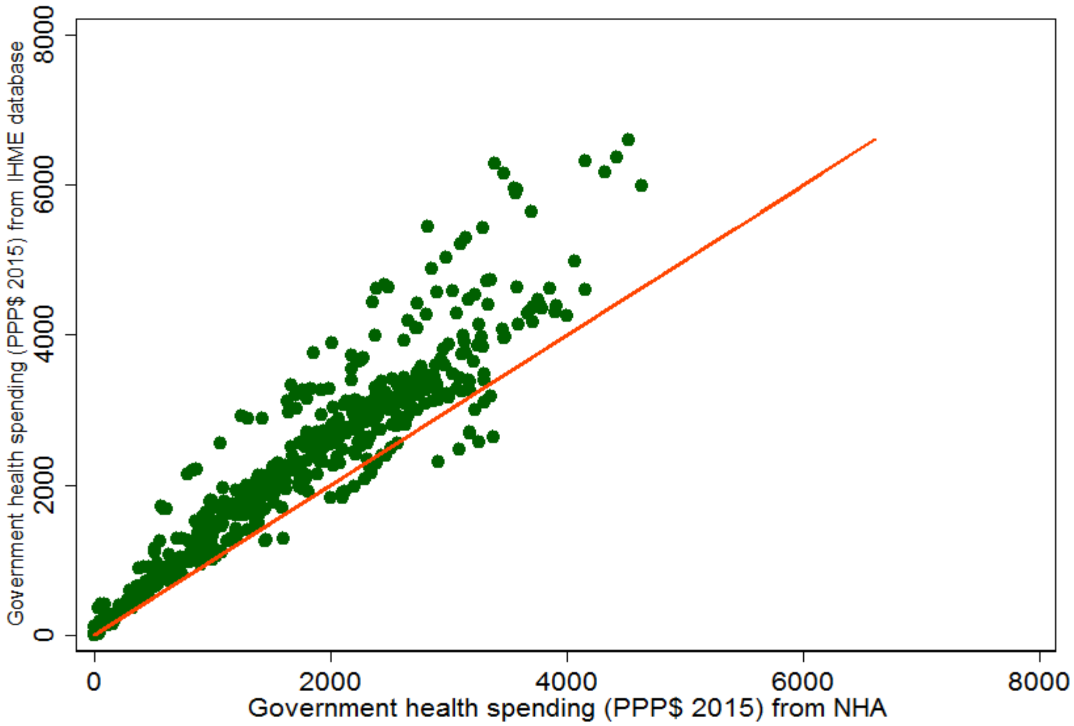

Notes: Differences between total and government health spending, in PPP $2015, differ somewhat between NHA and IHME database data. Notably, NHA health spending data generally report lower spending amounts than the IHME database, a discrepancy that is somewhat expected as not all "by type" spending categories are reported, thus leading to missed dollars. One country-year, Zambia 2002, had NHA data that was originally two orders of magnitude larger than the IHME database data, but when divided by 100 matched the IHME data closely.

Figure 20. Change in total health spending per capita, 1995–2010

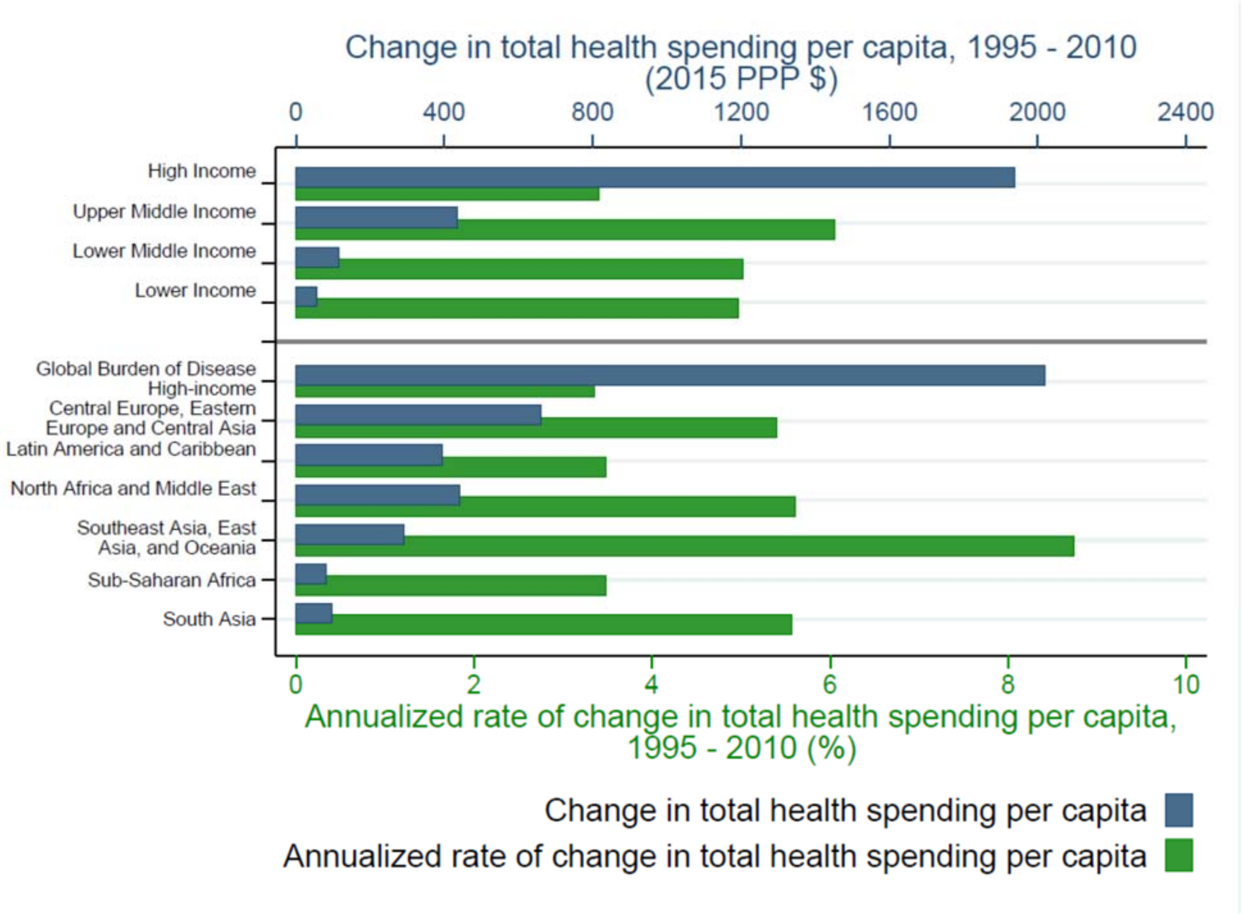

Figure 21. Change in total health spending per capita, 2010–2014

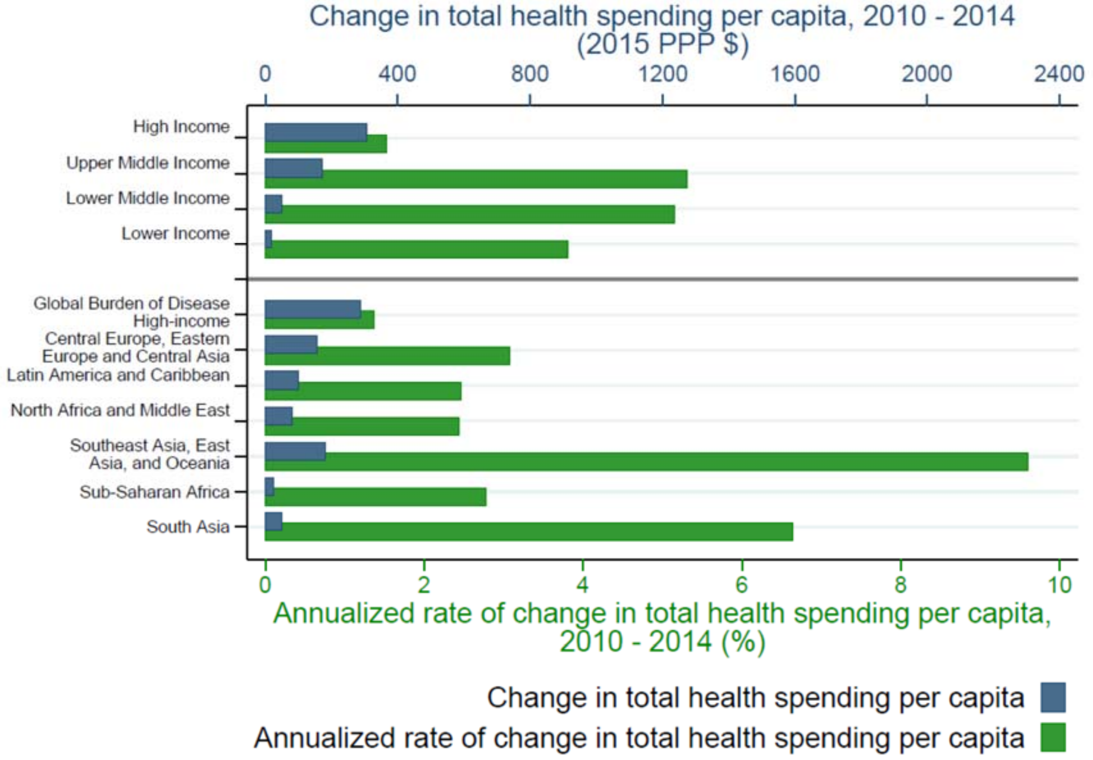

Figure 22. Change in total health spending per capita, 1995–2014

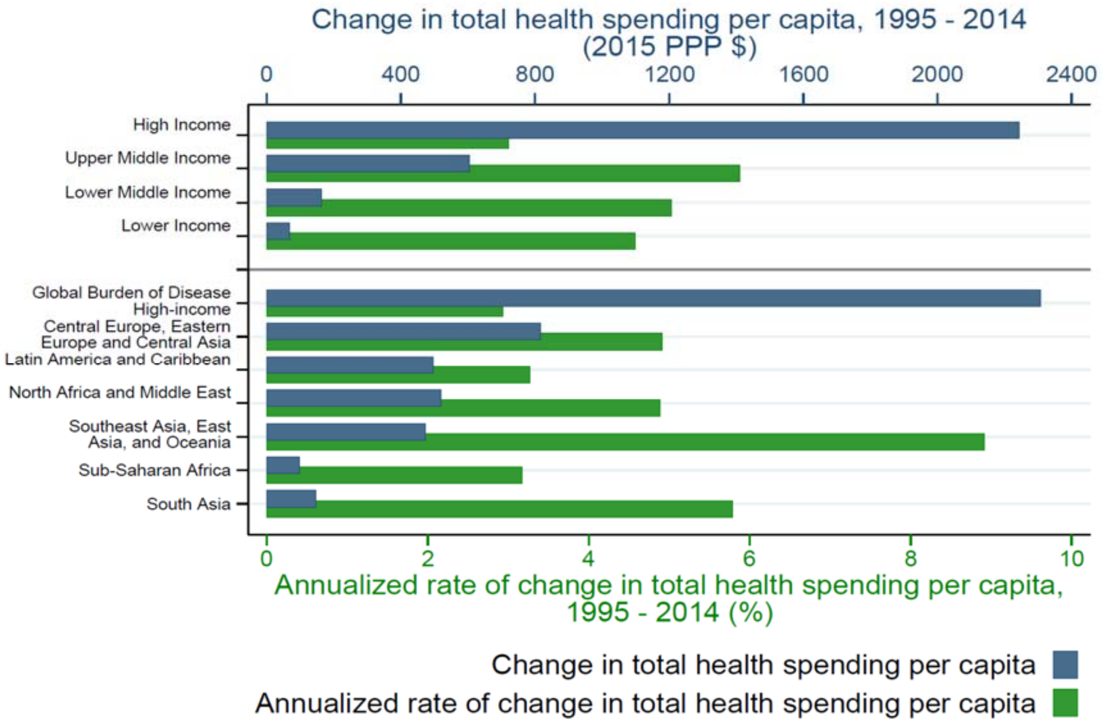

Figure 23. Health spending per capita by source and GDP per capita for all countries

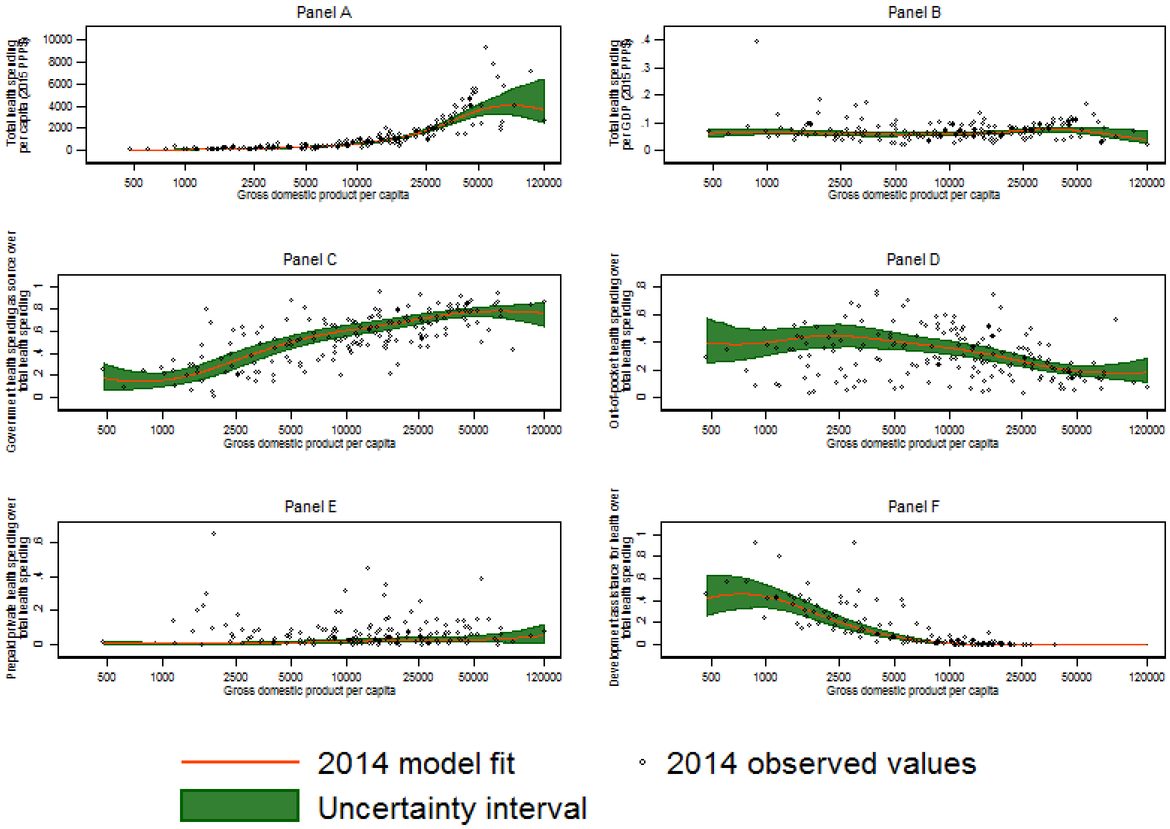

Figure 24. Observed government and total health spending relative to modeled spending for all countries

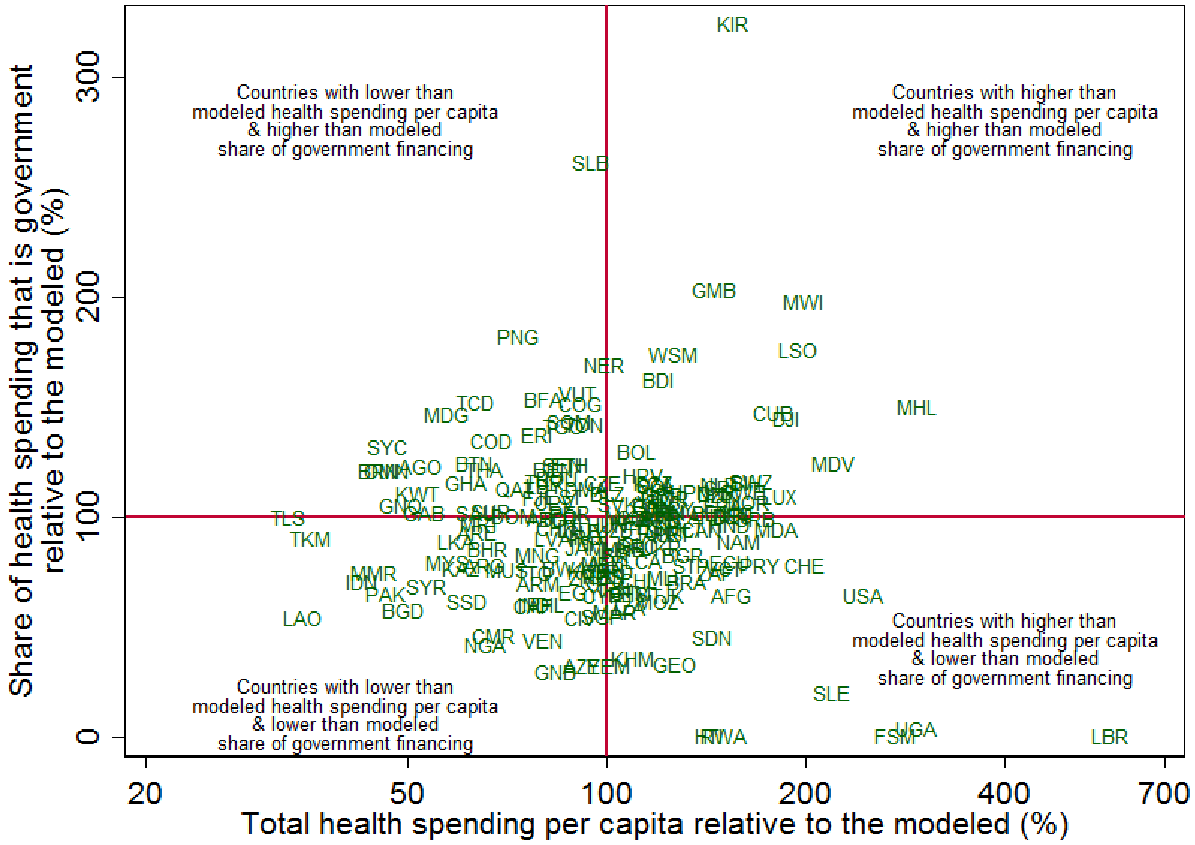

Author List

Corresponding author: Joseph Dieleman

Joseph Dieleman; Madeline Campbell; Abigail Chapin; Erika Eldrenkamp; Victoria Y Fan; Annie Haakenstad; Jennifer Kates; Yingying Liu; Taylor Matyasz; Angela Micah; Alex Reynolds; Nafis Sadat; Matthew T Schneider; Reed Sorensen; Tim Evans; David Evans; Christoph Kurowski; Ajay Tandon; Kaja M Abbas; Semaw Ferede Abera; Aliasghar Ahmad Kiadaliri; Kedir Yimam Ahmed; Muktar Beshir Ahmed; Khurshid Alam; Reza Alizadeh-Navaei; Ala'a Alkerwi; Erfan Amini; Walid Ammar; Stephen Marc Amrock; Carl Abelardo T Antonio; Tesfay Mehari Atey; Leticia Avila-Burgos; Ashish Awasthi; Aleksandra Barac; Oscar Alberto Bernal; Addisu Shunu Beyene; Tariku Jibat Beyene; Charles Birungi; Habtamu Mellie Bizuayehu; Nicholas J. K. Breitborde; Lucero Cahuana-Hurtado; Ruben Estanislao Castro; Ferran Catalia-Lopez; Koustuv Dalal; Pieter de Jager; Samath D. Dharmaratne; Manisha Dubey; Carla Sofia e Sa Farinha; Andre Faro; Andrea B Feigl; Florian Fischer; Joseph Robert Anderson Fitchett; Nataliya Foigt; Ababi Zergaw Giref; Rahul Gupta; Samer Hamidi; Hilda L Harb; Simon I. Hay; Delia Hendrie; Masako Horino; Mikk JÃ¼risson; Mihajlo B Jakovljevic; Mehdi Javanbakht; Denny John; Jost B Jonas; Seyed M. Karimi; Young-Ho Khang; Jagdish Khubchandani; Yun Jin Kim; Jonas M. Kinge; Kristopher J. Krohn; Hassan Magdy Abd El Razek; Mohammed Magdy Abd El Razek; Azeem Majeed; Reza Malekzadeh; Felix Masiye; Toni Meier; Atte Meretoja; Ted R Miller; Erkin M Mirrakhimov; Shafiu Mohammed; Vinay Nangia; Stefano Olgiati; Abdalla Sidahmed Osman; Mayowa O Owolabi; Tejas Patel; Angel J Paternina Caicedo; David M Pereira; Julian Perelman; Suzanne Polinder; Anwar Rafay; Vafa Rahimi-Movaghar; Rajesh Kumar Rai; Usha Ram; Chhabi Lal Ranabhat; Hirbo Shore Roba; Joseph Salama; Miloje Savic; Sadaf G Sepanlou; Mark G. Shrime; Roberto Tchio Talongwa; Braden J Te Ao; Fabrizio Tediosi; Azeb Gebresilassie Tesema; Alan J Thomson; Ruoyan Tobe-Gai; Roman Topor-Madry; Eduardo A Undurraga; Tommi Vasankari; Francesco S. Violante; Andrea Werdecker; Tissa Wijeratne; Gelin Xu; Naohiro Yonemoto; Mustafa Z Younis; Chuanhua Yu; Zoubida Zaidi; Maysaa El Sayed Zaki; Christopher J L Murray;

Affiliation List

Institute for Health Metrics and Evaluation, University of Washington, Seattle, WA, USA; ((Prof J Dieleman PhD, M Campbell BS, A Chapin BA, E Eldrenkamp BA, A Haakenstad MA, T Matyasz MS, A Micah PhD, N Sadat MA, M T Schneider MPH, R Sorensen MPH, Y Liu MS, A Reynolds BA, Prof S I Hay DSc, K J Krohn BA, J Salama, Prof C J L Murray DPhil); University of Hawaii at Manoa, Honolulu, HI, USA (V Y Fan ScD); François-Xavier Bagnoud Center for Health and Human Rights (V Y Fan ScD), Department of Global Health and Population, TH Chan School of Public Health (A B Feigl ScD), Harvard University, Boston, MA, USA (J R A Fitchett MD); Center for Global Development, Washington, DC, USA (V Y Fan ScD); Kaiser Family Foundation, Washington, DC, USA (J Kates PhD); World Bank, Washington, DC, USA (T Evans DPhil, D Evans PhD, C Kurowski MD, A Tandon PhD); Virginia Tech, Blacksburg, VA, USA (Prof K M Abbas PhD); School of Public Health, College of Health Sciences (S F Abera MSc), Mekelle University, Mekelle, Ethiopia (T M Atey MS, A G Tesema MPH); Food Security and Institute for Biological Chemistry and Nutrition, University of Hohenheim, Stuttgart, Germany (S F Abera MSc); Department of Clinical Sciences Lund, Orthopedics, Clinical Epidemiology Unit, Lund University, Lund, Sweden (A Ahmad Kiadaliri PhD); Debre Markos University, Debre Markos, Ethiopia (K Y Ahmed MPH); College of Health Sciences, Department of Epidemiology, ICT and e-Learning Coordinator, Jimma University, Jimma, Ethiopia (M B Ahmed MPH); Murdoch Childrens Research Institute (K Alam PhD), The University of

Melbourne, Melbourne, VIC, Australia (K Alam PhD); ., The University of Sydney, Sydney, NSW, Australia (K Alam PhD); Gastrointestinal Cancer Research Center, Mazandaran University of Medical Sciences, Sari, Iran (R Alizadeh-Navaei PhD); Luxembourg Institute of Health (LIH), Strassen, Luxembourg (A Alkerwi PhD); Uro-Oncology Research Center (E Amini MD), Endocrinology and Metabolism Research Institute (E Amini MD) Digestive Diseases Research Institute (Prof R Malekzadeh MD, S G Sepanlou PhD), Sina Trauma and Surgery Research Center (Prof V Rahimi-Movaghar MD), Tehran University of Medical Sciences, Tehran, Iran; Ministry of Public Health, Beirut, Lebanon (W Ammar PhD, H L Harb MPH); Oregon Health & Science University, Portland, OR, USA (S M Amrock MD); Department of Health Policy and Administration, College of Public Health, University of the Philippines Manila, Manila, Philippines (C A T Antonio MD); National Institute of Public Health, Cuernavaca, Mexico (L Avila-Burgos PhD, L Cahuana-Hurtado PhD); Sanjay Gandhi Postgraduate Institute of Medical Sciences, Lucknow, India (A Awasthi MSc); Faculty of Medicine, University of Belgrade, Belgrade, Serbia (A Barac PhD); University Andes, Bogota, Colombia (O A Bernal PhD); College of Health and Medical Science (A S Beyene MPH), College of Health and Medical Sciences (H S Roba MPH), Haramaya University, Harar, Ethiopia; Addis Ababa University, Addis Ababa, Ethiopia (T J Beyene MS, A Z Giref PhD); Wageningen University, Wageningen, Netherlands (T J Beyene MS); University College London, London, UK (C Birungi MS); Debre Markos University, Debre Markos town, Ethiopia (H M Bizuayehu MPH); Ohio State University, Columbus, OH, USA (Prof N J K Breitborde PhD); Universidad Diego Portales, Santiago, Chile (R E Castro PhD); Department of Medicine, University of Valencia/INCLIVA Health Research Institute and CIBERSAM, Valencia, Spain (F Catalá-López PhD); Clinical Epidemiology Program, Ottawa Hospital Research Institute, Ottawa, ON, Canada (F Catalá-López PhD); Centre for Injury Prevention and Safety Promotion, School of Health and Medical Sciences, Orebro University, Orebro, Sweden (Prof K Dalal PhD); School of Public Health, University of the Witwatersrand, Johannesburg, South Africa (P de Jager FCPHM(SA)); National Health Laboratory Service, National Institute for Occupational Health, Johannesburg, South Africa (P de Jager FCPHM(SA)); Department of Community Medicine, Faculty of Medicine, University of Peradeniya, Peradeniya, Sri Lanka (S D Dharmaratne MD); International Institute for Population Sciences, Mumbai, India (M Dubey MPhil, Prof U Ram PhD); DGS Directorate General of Health, Lisboa, Portugal (C S E S Farinha MSc); Universidade Aberta, Lisboa, Portugal (C S E S Farinha MSc); Federal University of Sergipe, Aracaju, Brazil (Prof A Faro PhD); School of Public Health, Bielefeld University, Bielefeld, Germany (F Fischer PhD); Institute of Gerontology, Academy of Medical Science, Kyiv, Ukraine (N Foigt PhD); West Virginia Bureau for Public Health, Charleston, WV, USA (R Gupta MD); Hamdan Bin Mohammed Smart University, Dubai, United Arab Emirates (S Hamidi DrPH); Oxford Big Data Institute, Li Ka Shing Centre for Health Information and Discovery, University of Oxford, Oxford, UK (Prof S I Hay DSc); Centre for Population Health Research (D Hendrie MA), Curtin University, Bentley, WA, Australia; Department of Health and Human Services, Nevada Division of Public and Behavioral Health, Carson City, NV, USA (M Horino MPH); Faculty of Medical Sciences, University of Kragujevac, Kragujevac, Serbia (Prof M B Jakovljevic PhD); University of Aberdeen, Aberdeen, UK (M Javanbakht PhD); International Center for Research on Women, New Delhi, India (D John MPH); Department of Ophthalmology, Medical Faculty Mannheim, Ruprecht-Karls-University Heidelberg, Mannheim, Germany (Prof J B Jonas MD); Institute of Family Medicine and Public Health, University of Tartu, Tartu, Estonia (M Jürisson MD); University of Washington Tacoma, Tacoma, WA, USA (S M Karimi PhD); College of Medicine, Seoul National University, Seoul, South Korea (Prof Y Khang MD); Ball State University, Muncie, IN, USA (J Khubchandani PhD); Southern University College, Skudai, Malaysia (Y J Kim PhD); Norwegian Institute of Public Health, Oslo, Norway (J M Kinge PhD, M Savic PhD); Mansoura Faculty of Medicine, Mansoura, Egypt (H Magdy

Abd El Razek MBBCH); Aswan University Hospital, Aswan Faculty of Medicine, Aswan, Egypt (M Magdy Abd El Razek MBBCh); Imperial College London, London, UK (Prof A Majeed MD); University of Zambia, Lusaka, Zambia (F Masiye); Martin Luther University Halle-Wittenberg, Halle (Saale), Germany (T Meier PhD); Department of Medicine (A Meretoja PhD), University of Melbourne, Footscray, VIC, Australia (Prof T Wijeratne MD); Department of Neurology, Helsinki University Hospital, Helsinki, Finland (A Meretoja PhD); Pacific Institute for Research & Evaluation, Calverton, MD, USA (T R Miller PhD); Centre for Population Health (T R Miller PhD), Curtin University, Perth, WA, Australia; Kyrgyz State Medical Academy, Bishkek, Kyrgyzstan (Prof E M Mirrakhimov PhD); National Center of Cardiology and Internal Disease, Bishkek, Kyrgyzstan (Prof E M Mirrakhimov PhD); Health Systems and Policy Research Unit, Ahmadu Bello University, Zaria, Nigeria (S Mohammed PhD); Institute of Public Health, Heidelberg University, Heidelberg, Germany (S Mohammed PhD); Suraj Eye Institute, Nagpur, India (V Nangia MD); State University of Bergamo, Bergamo, Italy (S Olgiati PhD); Public Health Institute Sudan, Khartoum, Sudan (A S Osman); Department of Medicine, Ibadan, Nigeria (M O Owolabi MD); Blossom Specialist Medical Center, Ibadan, Nigeria (M O Owolabi MD); Mount Sinai Health System, New York, NY, USA (T Patel MD); Universidad de Cartagena, Cartagena, Colombia (A J Paternina Caicedo MD); Public Health Dynamics Laboratory, University of Pittsburgh, Pittsburgh, PA, USA (A J Paternina Caicedo MD); REQUIMTE/LAQV, Laboratorio de Farmacognosia, Departamento de QuÃ-mica, Faculdade de FarmÃ¡cia, Universidade do Porto, Porto, Portugal (Prof D M Pereira PhD); National School of Public Health, Lisbon, Portugal (Prof J Perelman PhD); Department of Public Health, Erasmus MC, University Medical Center Rotterdam, Rotterdam, Netherlands (S Polinder PhD); Contech International Health Consultants, Lahore, Pakistan (A Rafay MS); Contech School of Public Health, Lahore, Pakistan (A Rafay MS); Society for Health and Demographic Surveillance, Suri, India (R K Rai MPH); Institute for Poverty Alleviation and International Development (C L Ranabhat PhD), Wonju College of Medicine (C L Ranabhat PhD), Yonsei University, Wonju, South Korea; Harvard Medical School, Boston, MA, USA (M G Shrime MD); Ministry of Health, MINSANTE, Yaounde, Cameroon (R T Talongwa MD); Auckland University of Technology, Auckland, new zealand (B J Te Ao MPH); Swiss Tropical and Public Health Institute, University of Basel, Basel, Switzerland (F Tediosi PhD); Adaptive Knowledge Management, Victoria, BC, Canada (A J Thomson PhD); National Center for Child Health and Development, Tokyo, Japan (R Tobe-Gai PhD); Institute of Public Health, Faculty of Health Sciences, Jagiellonian University Medical College, Krakow, Poland (R Topor-Madry PhD); Faculty of Health Sciences, Wroclaw Medical University, Wroclaw, Poland (R Topor-Madry PhD); Brandeis University, Waltham, MA, USA (E A Undurraga PhD); UKK Institute for Health Promotion Research, Tampere, Finland (Prof T Vasankari PhD); University of Bologna, Bologna, Italy (Prof F S Violante MD); Competence Center Mortality-Follow-Up of the German National Cohort, Federal Institute for Population Research, Wiesbaden, Germany (A Werdecker PhD); Western Health, Footscray, VIC, Australia (Prof T Wijeratne MD); Department of Neurology, Jinling Hospital, Nanjing University School of Medicine, Nanjing, China (Prof G Xu PhD); Department of Biostatistics, School of Public Health, Kyoto University, Kyoto, Japan (N Yonemoto MPH); Jackson State University, Jackson, MS, USA (Prof M Z Younis DrPH); Department of Epidemiology and Biostatistics, School of Public Health, Wuhan University, Wuhan, China (Prof C Yu PhD), Global Health Institute, Wuhan University, Wuhan, China (Prof C Yu PhD); University Hospital, Setif, Algeria (Prof Z Zaidi PhD); Faculty of Medicine, Mansoura University, Mansoura, Egypt (Prof M E Zaki PhD)

REFERENCES

1 Dieleman JL, Schneider MT, Haakenstad A, *et al.* Development assistance for health: past trends, associations, and the future of international financial flows for health. *The Lancet* 2016; **387**: 2536–44.

2 OECD. International Development Statistics (IDS) online databases. https://www.oecd.org/development/stats/idsonline.htm (accessed Jan 7, 2017).

3 European Commission. Annual reports - international cooperation and development. Int. Coop. Dev. /europeaid/annual-reports_en (accessed Jan 26, 2017).

4 UNAIDS. PCB Archive. http://www.unaids.org/en/aboutunaids/unaidsprogrammecoordinatingboard/pcbmeetingarchive (accessed Jan 26, 2017).

5 UNICEF. UNICEF integrated budget, 2014-2017. 2013. https://www.unicef.org/about/execboard/files/2013-ABL4-UNICEF_integrated_budget-11Jul2013.pdf.

6 UNICEF. United Nations Children's Fund financial report and audited financial statements. New York: United Nations.

7 UNICEF. Annual Report 2015. UNICEF. https://www.unicef.org/publications/index_92018.html (accessed Jan 30, 2017).

8 United Nations Population Fund. Annual Report 2015. http://www.unfpa.org/annual-report (accessed Jan 30, 2017).

9 Pan American Health Organization, World Health Organization. Executive Committee Session. 2016; published online Dec 20. http://www.paho.org/hq/index.php?option=com_content&view=category&id=1258&layout=blog&Itemid=1160&lang=en (accessed Jan 30, 2017).

10 World Health Organization. Financial report and audited financial statement, 2014. World Health Organization, 2015 http://apps.who.int/gb/ebwha/pdf_files/WHA68/A68_38-en.pdf.

11 The World Bank. Projects & Operations. http://projects.worldbank.org/ (accessed Jan 30, 2017).

12 Paris M. Project database 1990-2016 obtained through personal correspondence. 2016; published online Sept 29.

13 Asian Development Bank. Online project database. https://www.adb.org/projects (accessed Jan 30, 2017).

14 African Development Bank. Compendium of statistics on bank group operations. Tunis, Tunisia: Statistics Department, African Development Bank.

15 African Development Bank. Online project database. https://www.afdb.org/en/projects-and-operations/project-portfolio/ (accessed Jan 30, 2017).

16 Inter-American Development Bank. Projects database. http://www.iadb.org/en/projects/projects,1229.html (accessed Jan 30, 2017).

17 Neret M. health disbursement data. 2016; published online Dec 16.

18 Gavi, the Vaccine Alliance. Disbursements and commitments. http://www.gavi.org/results/disbursements/ (accessed Feb 27, 2017).

19 Gavi, the Vaccine Alliance. Pneumococcal AMC. http://www.gavi.org/funding/pneumococcal-amc/ (accessed Jan 30, 2017).

20 Gavi, the Vaccine Alliance. Financial reports. http://www.gavi.org/funding/financial-reports/ (accessed Feb 27, 2017).

21 Gavi, the Vaccine Alliance. Cash Received database. http://www.gavi.org/funding/donor-contributions-pledges/cash-receipts/ (accessed Feb 27, 2017).

22 The Global Fund to fight AIDS, Tuberculosis and Malaria. Grants in detail and Disbursements. http://www.theglobalfund.org/en/portfolio/ (accessed Jan 30, 2017).

23 The Global Fund to fight AIDS, Tuberculosis and Malaria. GFATM annual reports. 2017; published online Jan 30. http://www.theglobalfund.org/en/archive/annualreports/ (accessed Jan 30, 2017).

24 The Global Fund to fight AIDS, Tuberculosis and Malaria. GFATM pledges & contributions report. http://www.theglobalfund.org/en/ (accessed Jan 30, 2017).

25 GuideStar USA. Income tax filings. http://www2.guidestar.org/Home.aspx (accessed Jan 30, 2017).

26 United States Agency for International Development. VolAg report: report of voluntary agencies engaged in overseas relief and development. https://www.usaid.gov/pvo/volag-report (accessed Jan 30, 2017).

27 Thomson Reuters. Red book expanded database. New York: Thomson Reuters, 2009.

28 World Health Organization. WHO | Essential medicines. WHO. http://www.who.int/topics/essential_medicines/en/ (accessed Feb 15, 2017).

29 Bill & Melinda Gates Foundation. Audited Financial statements. Seattle, WA: Bill & Melinda Gates Foundation http://www.gatesfoundation.org/Who-We-Are/General-Information/Financials (accessed Jan 30, 2017).

30 Chan S. Foundation Awards and Payments. 2016; published online Aug 16.

31 Foundation Center. Grants database. Found. Cent. http://foundationcenter.org (accessed Jan 30, 2017).

32 Australian Government. Department of Foreign Affairs and Trade. Aid budget and statistical information. Dep. Foreign Aff. Trade. http://dfat.gov.au/aid/aid-budgets-statistics/Pages/default.aspx (accessed Feb 27, 2017).

33 Department of Foreign Affairs and Trade. Annual reports. Dep. Foreign Aff. Trade. http://dfat.gov.au/about-us/publications/corporate/annual-reports/pages/annual-reports.aspx (accessed Feb 15, 2017).

34 Austria Federal Ministry of Finance. Federal budget. https://www.bmf.gv.at/budget/das-budget/das-budget.html (accessed Jan 30, 2017).

35 Belgium House of Representatives. Project budgets. http://www.lachambre.be/kvvcr/showpage.cfm?section=/pri/budget&language=fr&rightmenu=right_pri&story=2013-budget.xml (accessed Jan 30, 2017).

36 Government of Canada. Planning and performance. GAC. http://international.gc.ca/gac-amc/publications/plans/index.aspx?lang=eng#rpp (accessed Jan 30, 2017).

37 Danish Ministry of Foreign Affairs. Foreign affairs budget. http://www.oes-cs.dk/bevillingslove/ (accessed Jan 30, 2017).

38 European Commission. General Budget - budget on-line. http://eur-lex.europa.eu/budget/www/index-en.htm (accessed Jan 30, 2017).

39 Ministry of Finance Finland. State budget bills [in Finnish]. http://budjetti.vm.fi/indox/index.jsp (accessed Jan 31, 2017).

40 Legifrance. Republique Francaise. Budget and financial documents. https://www.legifrance.gouv.fr/initRechTexte.do (accessed Jan 31, 2017).

41 Ministry of Foreign Affairs and International Development. Politique francaise en faveur du developpement. France: Ministry of Foreign Affairs and International Development, 2016 http://www.performance-publique.budget.gouv.fr/sites/performance_publique/files/farandole/ressources/2016/pap/pdf/DPT/DPT2016_politique_developpement.pdf.

42 German Federal Ministry of Economic Cooperation and Development. Plan of the federal budget. http://www.bmz.de/en/ministry/budget/index.html (accessed Jan 31, 2017).

43 Greece Standing Committee on Economic Affairs. The state budget and budgets for certain special funds and services, 2013 and 2014 [in Greek]. 2014. http://www.hellenicparliament.gr/UserFiles/7b24652e-78eb-4807-9d68-e9a5d4576eff/Proyp2014-prak.pdf.

44 Department of Finance, Government of Ireland. The Budget. http://www.budget.gov.ie/Budgets/2016/2016.aspx (accessed Jan 31, 2017).

45 Italian Agency for Development Cooperation. The new Italian Agency for Development Cooperation. http://pubdocs.worldbank.org/en/202961461601788644/Italian-Agency-for-Cooperation-presentation-March-2016.pdf.

46 Ministry of Finance Japan. Budget. http://www.mof.go.jp/english/budget/budget/index.html (accessed Jan 31, 2017).

47 Korea Official Development Assistance. Comprehensive implementation plan for international development cooperation. http://www.odakorea.go.kr/hz.blltn.PolicySlPL.do?blltn_div=oda (accessed Jan 31, 2017).

48 Ministry of Finance Luxembourg. State Budget[in French]. http://www.igf.etat.lu/.

49 New Zealand Treasury. Vote budget data - budgets of the New Zealand Government. 2017; published online Jan 31. http://www.treasury.govt.nz/budget (accessed Jan 31, 2017).

50 Ministry of Foreign Affairs Norway. Email correspondences. April 18, 2011, February 13, 2012, and August 14, 2013.

51 Royal Norwegian Ministry of Finance. National Budget. http://www.statsbudsjettet.no/Statsbudsjettet-2016/English/ (accessed Jan 31, 2017).

52 Ministry of Finance Portugal. State budget report. http://www.dgo.pt/Paginas/default.aspx (accessed Jan 31, 2017).

53 Ministry of Foreign Affairs and Cooperation Spain. Annual plan of cooperation (PACI). http://www.aecid.es/EN/cultura/Paginas/Publicaciones/Coop_Espanola/PACI/PACI.aspx (accessed Jan 31, 2017).

54 Ministry of Foreign Affairs Sweden. International aid budget. http://www.regeringen.se/ (accessed Jan 31, 2017).

55 Her Majesty's Treasury United Kingdom. Budget. https://www.gov.uk/government/publications/budget-2016-documents (accessed Jan 31, 2017).

56 UK - Department for International Development. IATI Dashboard. http://dashboard.iatistandard.org/publisher/dfid.html (accessed Feb 22, 2017).

57 Executive Office of the President of the United States. Budget of the United States Government. https://www.gpo.gov/fdsys/browse/collection.action?collectionCode=BUDGET&browsePath=Fiscal+Year+2014&isCollapsed=true&leafLevelBrowse=false&isDocumentResults=true&ycord=0 (accessed Jan 31, 2017).

58 US Foreign Assistance Dashboard. Foreign assistance by category, health-planned stage. http://beta.foreignassistance.gov/ (accessed Jan 31, 2017).

59 World Health Organization. Proposed programme budget. http://www.who.int/about/finances-accountability/budget/en/ (accessed Jan 31, 2017).

60 Joint United Nations Programme on HIV/AIDS. Financial report and audited financial statement. http://www.unaids.org/en/aboutunaids/unaidsprogrammecoordinatingboard/pcbmeetingarchive/ (accessed Jan 31, 2017).

61 Joint United Nations Programme on HIV/AIDS. Unified budget and workplan. http://www.unaids.org/en/aboutunaids/unaidsprogrammecoordinatingboard/pcbmeetingarchive/ (accessed Jan 31, 2017).

62 United Nations Children's Fund. Financial Report and Audited Financial Statement - Executive Board documents. https://www.unicef.org/about/execboard/index_25993.html (accessed Jan 31, 2017).

63 Sabbah L. Health expenditure data - UNICEF. 2015; published online Sept 29.

64 UNFPA. Report on contributions by member states and others to UNFPA and revenue projections. https://executiveboard.unfpa.org/ (accessed Jan 31, 2017).

65 UNFPA. Statistical and Financial review - Audited Financial report. https://executiveboard.unfpa.org/ (accessed Jan 31, 2017).

66 Smithson M, Verkuilen J. A better lemon squeezer? Maximum-likelihood regression with beta-distributed dependent variables. *Psychol Methods* 2006; **11**: 54–71.

67 Aid data. Open aid data: sectors. http://www.openaiddata.org/sectors/all/ (accessed Feb 21, 2017).

68 Honaker J, King G. What to do about missing values in time-series cross-section data. *Am J Polit Sci*; **54**: 561–81.

69 Honaker J, King G, Blackwell M. Amelia II: A program for missing data. *J Stat Softw*; **45**: 1–47.

70 Bui AL, Lavado RL, Johnson EK, *et al.* National health accounts data from 1996 to 2010: a systematic review. *Bull WORLD Health Organ* 2015; : 566–76.

71 OECD, Eurostat, WHO. A system of health accounts. 2011 doi: 10.1787/9789264116016-en.

72 OECD. A System of Health Accounts. Version 1.0. Organization for Economic Co-Operation and Development, 2000 http://www.who.int/health-accounts/documentation/EnglishSHA.pdf?ua=1.

73 Eurostat. Healthcare expenditure statistics - methodology - Statistics Explained. 2017. http://ec.europa.eu/eurostat/statistics-explained/index.php/Healthcare_expenditure_statistics_-_methodology#Main_concepts_and_definitions (accessed Feb 25, 2017).

74 James SL, Gubbins P, Murray CJ, Gakidou E. Developing a comprehensive time series of GDP per capita for 210 countries from 1950 to 2015. *Popul Health Metr* 2012; **10**: 12.

75 Smithson M, Verkuilen J. A better lemon squeezer? maximum-likelihood regression with beta-distributed dependent variables. *Psychol Methods* 2006; **11**: 54–71.

76 Pawlowsky-Glahn V, Buccianti A. Compositional data analysis: theory and applications. John Wiley & Sons, 2011.

77 Aitchison J. The statistical analysis of compositional data. *J R Stat Soc Ser B Methodol* 1982; **44**: 139–77.

78 Wood SN. Fast stable restricted maximum likelihood and marginal likelihood estimation of semiparametric generalized linear models. *J R Stat Soc Ser B Stat Methodol* 2011; **73**: 3–36.

79 UNDP. Human Development Data (1980-2015). http://hdr.undp.org/en/data (accessed Jan 5, 2017).

80 Wang H, Naghavi M, Allen C, *et al.* Global, regional, and national life expectancy, all-cause mortality, and cause-specific mortality for 249 causes of death, 1980–2015: a systematic analysis for the Global Burden of Disease Study 2015. *The Lancet* 2016; **388**: 1459–544.
